# How public trust and healthcare quality relate to blood donation behavior: Cross-cultural evidence

**DOI:** 10.1177/13591053231175809

**Published:** 2023-06-05

**Authors:** Caroline Graf, Bianca Suanet, Pamala Wiepking, Eva-Maria Merz

**Affiliations:** 1 Vrije Universiteit Amsterdam, The Netherlands; 2 Department of Donor Medicine Research, Sanquin Research, The Netherlands; 3 Indiana University–Purdue University Indianapolis (IUPUI), USA

**Keywords:** blood donation, cross-cultural evidence, healthcare quality, public trust

## Abstract

Blood donors are indispensable for enabling a myriad of medical procedures and treatments. We examined how public trust in the healthcare system and healthcare quality relate to individuals’ likelihood of donating blood, using survey data from representative samples of 28 European countries (*N* = 27,868). Our preregistered analyses revealed that country-level public trust, but not healthcare quality, predicted individual propensity to donate blood. Notably, public trust decreased over time in many countries, while healthcare quality increased. Our results highlight the role of subjective *perceptions* of the healthcare system, rather than the objective state of healthcare, for blood donation behavior in Europe.

## Introduction

Ensuring a continuous and sufficient supply of blood is crucial for all societies, as blood plays an essential part in many medical procedures (e.g. surgeries, cancer treatment) and for producing life-saving drugs. Since blood cannot (yet) be artificially produced or stockpiled, blood collectors rely on willing donors to give their blood. These blood donations take place in a medical setting and are embedded within the larger healthcare system, that is, donations are collected by health professionals who also monitor donor health, and blood banks coordinate the supply and demand of blood with hospitals. The specific medical context in which individuals donate blood may vary across regions and countries, because countries organize blood collection differently (Healy, 2000). Countries also differ in their healthcare systems and overall quality of healthcare (Fullman et al., 2018). Indeed, the manner in which these specific (organizational) environments affect individuals’ decisions to donate blood has become an important topic of research (Gorleer et al., 2020; Healy, 2000) and accordingly the importance of the geographical *context* in which the donation takes place has been recognized (Cimaroli et al., 2012; Masser et al., 2020; Piersma et al., 2021). But blood collecting institutions do not only vary in their organization of blood collection, they also vary with respect to the *cultural* setting in which they operate. The beliefs, values, and social norms prevalent in a given cultural setting have been shown to play a key role in shaping the social behavior of individuals (Henrich et al., 2010; Hofstede, 2001; Inglehart, 2000). Yet, despite this prevalent role of cultural factors in influencing behavior in other domains, there have thus far been only few studies examining how these factors affect human substance donation (but there are exceptions, e.g. Merz et al., 2016; de Kort et al., 2010; Gillum and Masters, 2010; Tison et al., 2007). The current study contributes to this growing literature by examining how characteristics of the healthcare system, including cultural attitudes toward the system, relate to blood donation behavior.

We focus on two characteristics of the healthcare system specifically: People’s *perception* of the healthcare system (i.e. public trust in the healthcare system) and more objective measures of the *performance* of the health care system (i.e. quality of healthcare). Firstly, we expect higher *healthcare quality* to be associated with higher propensity of individuals to donate blood. Previous research has highlighted several pathways through which healthcare quality may influence blood donation behavior. For example, people in well functioning healthcare systems should have less fear of medical errors and hospitals, which remain common deterrents to donate blood (Boulware et al., 2002; Klinkenberg et al., 2021; Masser et al., 2011). Similarly, individuals with access to high quality healthcare should perceive healthcare services in general, and those pertaining to blood donation in particular, as more effective. Donors have in turn been shown to be more motivated to give blood when they perceive the blood bank to operate efficiently (Giles et al., 2004; Masser et al., 2008). Although these theoretical links point toward a connection between healthcare quality and blood donation behavior, a direct link has (to our knowledge) not been established. Nonetheless, indirect evidence for such a relationship exists. In countries with higher life expectancy (which is indicative of better healthcare quality), people perceive the procedure of blood transfusions to be safer (Merz et al., 2016). In turn, perceived blood transfusion safety predicts blood donation willingness (Huis In 't Veld et al., 2019). Accordingly, our first hypothesis is the following:

- H1: Higher country-level *quality of healthcare* is associated with higher individual-level propensity to donate blood.

When people live in a country with higher *public trust* in the healthcare system, this should also be positively related to people’s donation behavior. By a similar logic as the one sketched above for healthcare quality, higher public trust should be related to lower levels of fear and higher perceived efficacy of healthcare institutions, including those responsible for collecting blood. In fact, trust has been found to be an important motivating factor for blood donors, but is typically studied at the individual level. That is, individual-level generalized trust (Merz et al., 2017; Boenigk et al., 2015) as well as individual-level trust in blood collection agencies (Chen, 2017) have been positively associated with blood donation behavior. Here we focus on *public* trust in the healthcare system measured at the country-level. Public trust in healthcare reflects more than an individual’s experience with the healthcare system, for example by interacting with individual physicians. Instead, it is a product of how the healthcare system is portrayed in the media and how experiences are debated in the public discourse, in addition to “objective” characteristics of the system and individuals’ experiences in it (Gille et al., 2017). While the measure of public trust employed in the current study is also based on (aggregated) individual-level trust, we contribute a novel cross-cultural perspective. Culture can have a significant influence on how trust is perceived and developed, by shaping people’s expectations about the trustworthiness of others and their willingness to trust in different situations. Cultural factors such as individualism, power distance or tightness–looseness all have implications for trust processes (Fulmer and Gelfand, 2012). For example, people in collectivistic cultures tend to have lower generalized trust than people in individualistic cultures (Bohnet et al., 2010; Realo et al., 2008). Moreover, people from different countries are exposed to different media portrayals and information campaigns. Hence, our measure captures more than the individual-level trust measures that are typically examined within a single population, where all people experience a similar social discourse and cultural ecosystem. Importantly, public trust in the healthcare system also plays a key role in the effective functioning of healthcare systems, as well as for individual-level health outcomes (Abbott and Freeth, 2008; Cuevas et al., 2019; Gille et al., 2015; Gilson, 2003; Pilgrim et al., 2010). For example, medical mistrust has been found to be an important deterrent for rural women’s ability and willingness to obtain healthcare (Statz and Evers, 2020), and has played a key role in vaccine hesitancy during the ongoing COVID-19 pandemic (Vergara et al., 2021). It remains to be seen whether public trust in the healthcare system is also related to blood donation behavior. Hence, we formulate our second hypothesis:

- H2: Higher country-level *trust in the healthcare system* is associated with higher individual-level propensity to donate blood.

## Materials and methods

### Participants and procedure

We employed comparative data from the 2014 wave of the Eurobarometer (European Commission, 2018), a repeated cross-sectional survey conducted among representative samples from European Union member states. In 2014, the survey was performed in 28 countries. A multi-stage random (probability) sampling design was employed and approximately 1000 face-to-face interviews were completed in each of the countries. Respondents were residents in the respective country, had sufficient command of the national language(s), and were 15 years or older. Study protocols received ethical approval by the European Commission and all participants provided informed consent. A detailed description of all variables, including the wording of survey items and measurement scales, is presented in Supplemental Table S1. All categorical variables, including the dependent variable, are dummy-coded.

### Dependent variable

#### Blood donation

The dependent variable in our analyses is blood donation during one’s lifetime (yes/no). Participants in the Eurobarometer were asked whether they have ever donated substances of human origin, including blood.

### Independent variables

#### Public trust in the healthcare system

We measured country-level public trust in the healthcare system by employing survey data from the 1999 and 2008 rounds of the European Values Survey (EVS, 2021). The EVS asked respondents from all European countries about their confidence in different institutions, including the healthcare system. The term *confidence* is sometimes used when the target of trust is an institution instead of an individual (this is also termed institutional trust or system trust; PytlikZillig and Kimbrough, 2016). In this study we use the term *public trust*. In order to calculate country-level public trust for each of the European countries, we aggregated individual responses to the question: “How much confidence do you have in the healthcare system?” at the country-level. The original EVS item was on a 4-point scale (none at all; not very much; quite a lot; a great deal), which we coded as 1; 2; 3; 4; Hence the range of the country-level indicator was from 1 to 4. Data from the EVS on trust in the healthcare system is available for all 28 EU countries included in our main analyses for the years 1999 and 2008 (except Cyprus for 1999).

#### Quality of healthcare

We took a two-fold approach to operationalizing country-level quality of healthcare. That is, we included (a) the healthcare access and quality index (HAQ; Global Burden of Diseases Collaborative; Fullman et al., 2018) as a measure of how well a country manages to prevent amenable deaths and (b) a measure capturing healthcare expenditures relative to a country’s gross domestic product (GDP; World Health Organization Global Health Expenditure database; World Health Organization [WHO], 2015). More specifically, the HAQ index is a globally comparable combined index reflecting the extent of amenable mortality (i.e. death rate from 32 causes of death that should not occur if medical care is effective, e.g. tuberculosis or diphtheria) across 195 countries and territories. The index ranges from 0 to 100, where 0 (100) reflects the lowest (highest) observed levels of disease prevention across countries from 1990 to 2015. The HAQ index is available for all European countries included in our sample for the years 1990, 1995, 2000, 2005, 2010, and 2015. The WHO indicator for healthcare expenditures per GDP is a measure of total healthcare expenditure (i.e. both public and private spending on preventive and curative health services) relative to a country’s GDP. The theoretical range of this indicator is from 0% to 100% of GDP. Healthcare expenditure is available for all countries in our sample for the years 1995–2014.

#### Demographics

We controlled for individual socio-demographic factors, which previous studies had shown to be related to propensity to donate (see Piersma et al. (2017) for a recent review on determinants of blood donation). We included (a) age (as our dependent variable is dependent on years lived), (b) gender (male/female), (c) education (age when stopped full-time education), (d) partner status (living with partner: yes/no), (e) employment status (employed: yes/no), (f) parental status (operationalized as children in household: yes/no), and (g) type of community (three levels: large town; small/middle-sized town; rural area).

### Matching country-level indicators by year

In order to account for indicator variation over time, we exploited that all three country-level variables of public trust and healthcare quality were available for multiple time points. Specifically, we matched indicator values to respondents from the Eurobarometer by presumed year of blood donation (following a similar matching approach to account for temporal variation as Spadaro et al., 2022). As we did not know when exactly respondents donated blood, we took as an approximation the average time point within the age range that respondents are eligible to donate (in most European countries the permitted age range for blood donation is 18–65 years). The estimated donation time point_r_ was calculated in the following manner:

donation time point_r_

$$\left\{ \begin{array}{ll} 2014-\left({\mathrm{age}}_{r}-18\right)/2 & \mathrm{if}\;{\mathrm{age}}_{r}=<65\\ 2014-(65-18)/2-\left({\mathrm{age}}_{r}-65\right) & \mathrm{if}\;{\mathrm{age}}_{r}>65 \end{array}\right.$$

Here *donation time point_r_* is the assumed time point of donation of respondent r; 2014 is the time of the survey; age_r_ is the age of respondent r at that time; 18 is the minimum and 65 is the maximum age for blood donation in most European countries. As an example, a respondent who is 22 years old at the time of the study has a *donation time point* of 2012; a respondent who is 48 years old has a *donation time point* of 1999; and a respondent who is 80 years old has a *donation time point* of 1975.5.

### Statistical analyses

#### Exclusions

Respondents younger than 18 years were excluded since most EU countries do not permit people under 18 to donate blood.

#### Multilevel modeling

We employed multilevel logistic mixed models for all main analyses, where individuals were nested in countries. The models included individual-level sociodemographic variables and country-level variables (i.e. healthcare quality and public trust) as fixed effects, as well as allowing the intercept to vary by country and by indicator survey wave. The random intercepts were crossed random effects (i.e. each participant was nested within a country and one or multiple survey waves; wave and country were not nested). The estimation technique is maximum likelihood. All tests are two-sided. Missing values were handled by list-wise deletion.

We first tested for the significance of the random components (country and survey wave) of the baseline model by performing log-likelihood-ratio tests of models with and without constrained variance. Then we added the individual-level socio-demographic variables to the model, and finally ran models with the country-level predictors. The limited number of countries in our sample (*k* = 28) constrained the number of country-level predictors that could be included in the model simultaneously (Bryan and Jenkins, 2016). Hence we implemented two separate models for (a) public trust and (b) healthcare quality (including HAQ index and healthcare expenditures). Prediction intervals are based on fixed coefficients and observation-level errors. All analyses were run with R version 4.0.2 (R Core Team, 2020) using the packages lme4 (modeling), merTools (predictions), tidyverse (data transformation; plotting) and rnaturalearth (maps) (Bates et al., 2007; Knowles and Frederick, 2016; South, 2017; Wickham et al., 2019).

## Results

### Sample descriptives and country-level indicators over time

Overall, 38.4% (*n* = 10,195) of all respondents had ever donated blood. Mean levels of blood donation at the country-level ranged from 22.8% of respondents from Portugal having donated blood to 52.9% of respondents from France having donated blood (see Supplemental Figure S1). Table 1 gives an overview of descriptive statistics of our sample. Supplemental Table S2 provides indicator values for each country. The final dataset comprised 26,532 participants, which excluded 786 participants (2.8%) who did not reply to the dependent variable of donating blood and 550 participants (2.0%) under the age of 18 who were not eligible to donate at the time of the survey. There was no missing data in the country-level independent variables, and minimal missing data in the covariates (see Table 1).

**Table 1.** Descriptive statistics for the dependent and independent variables.

Level	Variable	Range	% yes	Mean	Std.	N
Individual-level	Blood donation	0; 1	38%	-	-	26,532
	Gender (female = 1)	0; 1	56%	-	-	26,532
	Age (years)	18–99	-	51.29	17.81	26,532
	Education	0–89	-	19.24	5.53	26,075
	Living with partner	0; 1	65%	-	-	26,476
	Employed	0; 1	49%	-	-	26,532
	Children in household	0; 1	19%	-	-	26,527
	Type of community					
	Large town (ref.)	0; 1	27%	-	-	26,518
	Mid-sized town	0; 1	42%	-	-	26,518
	Rural area	0; 1	31%	-	-	26,518
Country-level	Trust in healthcare system	1.84–3.23	-	2.61	0.31	26,532
	HAQ index	58.3–90.5	-	75.56	6.93	26,532
	Healthcare expenditures	3.21–11.97	-	7.52	1.64	26,532

Sources: Eurobarometer (European Commission, 2018); European Values Survey (EVS, 2021); Healthcare access and quality index (Global Burden of Diseases Collaborative; Fullman et al., 2018); World Health Organization Global Health Expenditure database (WHO, 2015).

**Figure 1.**
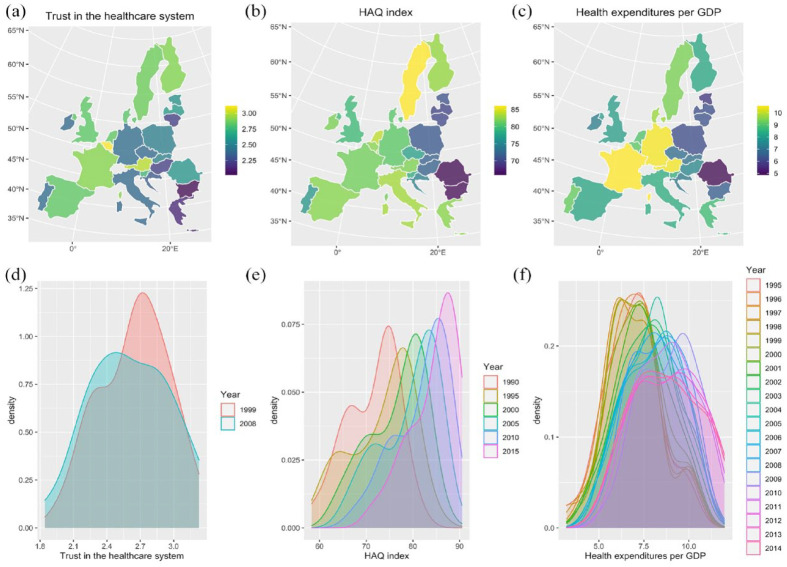
Distribution of country-level indicators across countries and over time. First row: Maps displaying (a) trust in the healthcare system, (b) healthcare quality as measured by the HAQ index and (c) healthcare quality as measured by healthcare expenditures per GDP. Second row: Density plots displaying (d) trust in the healthcare system, (e) healthcare quality as measured by the HAQ index and (f) healthcare quality as measured by healthcare expenditures per GDP.

**Figure 2.**
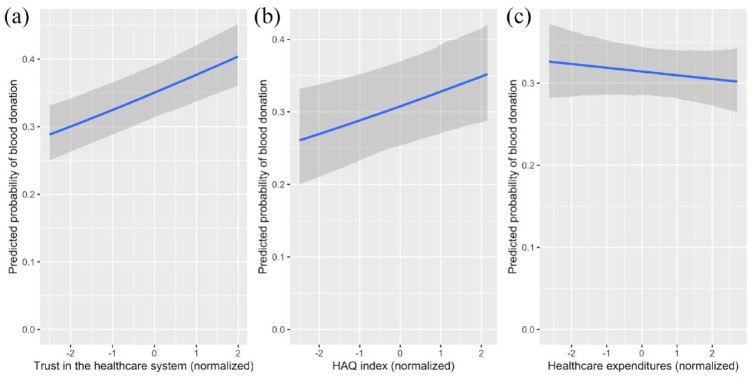
Predictive margins of donating blood for a representative European woman (i.e. mean aged, living with a partner without children in a small town, employed, and having completed full-time education at age 19) as a function of (a) trust in the healthcare system, (b) healthcare quality as measured by the HAQ index and (c) healthcare quality as measured by healthcare expenditures per GDP. Prediction bands indicate 80% prediction intervals.

We found significant variation in all three country-level indicators across countries (see Figure 2; trust in the healthcare system: X^2^(27) = 7434, *p* < 0.001; HAQ index: X^2^(27) = 429.51, *p* < 0.001; healthcare expenditures: X^2^(27) = 78.71, *p* < 0.001). Notably, levels of trust in the healthcare system and healthcare quality were relatively high across all countries, which is not surprising given that most European countries are industrialized countries. However, there was also some geographical variation in public trust, such that trust appeared to be higher in Western and Northern European countries compared to Eastern and Southern European countries (see Figure 1(a)). Both healthcare quality indicators also exhibited a divide between Eastern and Western Europe, such that healthcare quality tended to be lower in Eastern than in Western European countries (see Figure 1(b) and (c)). The appendix provides further maps depicting indicator variation across countries for different time points (Supplemental Figures S2–S4).

Not only was there variation of country-level indicators across countries, but indicators also varied across time. More specifically, public trust overall *decreased* over time (Figure 1(d)), whereas healthcare quality both in terms of the HAQ index and healthcare expenditures *increased* (Figure 1(e) and (f)). Indeed, the positive correlations between survey time point and the two healthcare quality indicators were strong (HAQ index: Pearson’s *r* = 0.99, *p* < 0.001; healthcare expenditures: Pearson’s *r* = 0.97, *p* < 0.001). When examining trends over time at the country-level, we find that trust in the healthcare system decreased between 1999 and 2008 in many countries (i.e. there is a decrease in public trust in 17 out of 28 countries, see Supplemental Figure S5). HAQ index continuously increases over time for all countries (except for the Baltic states Lithuania, Estonia, and Latvia between 1990 and 1995; Supplemental Figure S6). For the annual data on healthcare expenditures per GDP there is greater fluctuation across years (see Supplemental Figure S7), but overall all countries exhibit mild to strong increases in healthcare expenditures across time (see Supplemental Figure S8).

### Main results

As hypothesized, we found a positive main effect of trust in the healthcare system on blood donation propensity (*b* = 0.114, *p* < 0.001; Model 2 in Supplemental Table S3). However, neither measure of healthcare quality was statistically significantly associated with blood donation behavior (HAQ: *b* = 0.101, *p* = 0.07; healthcare expenditures: *b* = −0.020, *p* = 0.628; Model 3 in Supplemental Table S3). A visualization of the model results is presented in Figure 2, which plots the predictive margins of donating blood for a representative European woman against different levels of public trust (Figure 2(a)), HAQ index (Figure 2(b)), and healthcare expenditures (Figure 2(c)). As illustrated in Figure 2(a), the results indicate that respondents from countries where trust in the healthcare system is high are more likely to have donated blood. The association between blood donation behavior and healthcare quality measured by the HAQ index is also positive (Figure 2(b)), albeit with higher uncertainty around the estimate (as also indicated by a lack of statistical significance in the model). Lastly, the likelihood of having donated was not higher for individuals residing in countries with higher healthcare expenditures per GDP (Figure 2(c)) compared to those living in countries with lower healthcare expenditures.

Model 1 further revealed that several individual-level socio-demographic variables are associated with blood donation behavior, largely in line with previous studies (e.g. Piersma et al., 2017). In particular, older, male, employed and more educated individuals, and those living with a partner were more likely to have donated blood during their lifetime (see Supplemental Table S3 for an overview).

### Additional results

All models included random intercepts for country, which a log-likelihood test comparing the intercept-only model with and without random intercepts confirmed to be justified (χ^2^(1) = 501.79, *p* < 0.001). Moreover, intercepts were allowed to vary by survey wave, which also proved to be justified by log-likelihood tests (wave_trust_: χ^2^(1) = 144.6, *p* < 0.001; wave_HAQ_: χ^2^(1) = 214.33, *p* < 0.001; wave_expenditures_: χ^2^(1) = 183.07, *p* < 0.001). Including survey wave random effects is particularly important for two reasons: Firstly, our dependent variable is dependent on time (i.e. we measure blood donation *during the lifetime*, which results in older individuals being more likely to have donated blood) and secondly, the country-level indicators vary systematically over time (i.e. public trust decreased, but healthcare quality increased over time). Hence, not accounting for survey wave could lead to spurious effects driven by older individuals being more likely to have donated blood, but having donated during a time when public trust was higher and healthcare quality was lower compared to the time when younger individuals donated. In accordance with this reasoning, an additional model without survey wave random effects (see Supplemental Table S3, Model 5) showed that public trust is positively associated with donating blood, while the HAQ index and healthcare expenditures are negatively associated with donation behavior. These negative effects of HAQ index and healthcare expenditures can be explained by systematic temporal variation. Indeed, this interpretation is also in line with the descriptive results, which illustrate that country-level public trust, HAQ index and healthcare expenditures are all positively related to country-level mean levels of blood donation (Supplemental Figure S9). Note that further robustness checks can be found in the appendix.

## Discussion

In this paper we investigated the relationship between blood donation, public trust in the healthcare system and healthcare quality. As predicted, we found a positive association between public trust in the healthcare system and blood donation: the higher trust is in a country, the higher an individual’s probability of having donated blood. However, neither measure of objective healthcare quality was statistically significantly related to blood donation behavior. While the three country-level indicators of public trust and healthcare quality exhibited similar spatial distributions across countries, we found an interesting pattern in the distribution of indicators over time. That is, trust in the healthcare system *decreased* between 1999 and 2008, whereas healthcare quality *increased* between 1990 and 2015 for many countries.

Our findings shed new light on how perceptions and objective characteristics of the healthcare system relates to blood donation behavior, but several open questions remain for future research to explore. Even though we found that a relatively simple measure of trust in the healthcare system predicts blood donation behavior, a more fine-grained measure of public trust would aid in further understanding the results (see e.g. Ghoshal et al., 2022). In particular, our measure of trust potentially captures different related concepts, such as trust in the healthcare system as a whole, trust in health institutions (e.g. hospitals), as well as trust in healthcare professionals (e.g. physicians; van der Schee et al., 2007). Our results cannot distinguish between these different targets of trust. Related to this issue, independent blood banks may not be perceived as part of the healthcare system in all countries, since European countries vary in their blood collection systems and in some countries blood banks operate relatively independently (Healy, 2000). Moreover, our temporal indicator matching was a relatively crude strategy involving multiple assumptions. We followed previous studies in matching indicators by year to capture temporal variation (e.g. Acemoglu et al., 2021; Spadaro et al., 2022), but our matching procedure was limited by uncertainty around estimating individuals’ donation time point. Despite this uncertainty, we found it to be important to account for temporal variation in trust and healthcare quality when conceiving the study (see preregistration), and temporal matching presented the best solution given the available data. Some exploratory analyses suggest that the temporal variation in trust may play an important role for the association between public trust and blood donation behavior (see Supplemental Table S7 in the Appendix), thus we encourage future studies to collect more comprehensive datasets to investigate these temporal patterns further (e.g. by using longitudinal cross-country data on individuals’ blood donations in the previous year). Lastly, our study is correlational and hence cannot establish the causal relationship between characteristics of the healthcare system and blood donation behavior.

Furthermore, our results highlight the importance of subjective factors, such as public trust, opposed to objective measures of healthcare quality, for blood donation behavior. This is in line with research emphasizing the key role of affective attitudes and beliefs for donating substances of human origin, such as blood (Conner et al., 2013; Farley and Stasson, 2003; Morgan et al., 2008). This uncoupling between the objective level of the healthcare quality and subjective trust in the healthcare system presents an intriguing puzzle in itself, which would benefit from further research. Relatedly, it remains an interesting topic for future studies to establish which factors influence public trust if not the objective performance of the healthcare system, and what their respective contribution is (e.g. to what extent debates in the public discourse affect public trust; which cultural factors influence trust). To this end it is crucial to understand where cultural differences exist, and how these relate to (health) behaviors such as blood donation. Not only does cross-cultural evidence inform psychological research by testing the generalizability of findings that typically stem from American or Western European samples (see Henrich et al., 2010), but a better understanding of these mechanisms would also aid in designing effective interventions for building and sustaining public trust in the healthcare system. Since we found cross-cultural variation in trust, which in turn predicted blood donation behavior, one promising strategy to boost blood donations could be to invest more in fostering trust in the healthcare system, for example by engaging in earnest messaging that signals dependability and trustworthiness. Moreover, our cross-cultural findings offer clues toward understanding under which circumstances interventions produce their intended beneficial outcomes. For example, an information campaign by the government to encourage positive health behaviors may only be successful if there exists a sufficient amount of trust in institutions in the population. For any intervention, special attention should be given to communities with particularly low trust in the healthcare system, such as those with members of minority or religious groups, who are more likely to have experienced discrimination and to suffer from lack of representation within the healthcare system (Musa et al., 2009; Renzaho et al., 2013).

More broadly, our findings have important implications in light of what has been called a “Global Trust Crisis” (Algan et al., 2017). This crisis describes the concerning observation that trust in institutions, including trust in government and in science, has seen a decline across several decades (Citrin and Stoker, 2018; Gauchat, 2012; Hardin and Offe, 1999; Mechanic, 1996; Perry, 2021). Here we find that *trust in the healthcare system* also decreases between 1999 and 2008 for many countries in our sample. This is particularly surprising given the continuous increase in healthcare quality over time in virtually all European countries. Although medicine has historically been one of the most trusted institutions (Mechanic, 1996), the decrease of trust in the healthcare system observed here is also reflected in other domains, for instance in significant vaccine hesitancy during the current COVID-19 pandemic (Dror et al., 2020). This erosion of institutional trust has been warned to result in a decline in effectiveness of government and democratic stability (Citrin and Stoker, 2018). Indeed, a decline in trust in institutions is related to various negative societal outcomes, including lower political participation (Putnam, 2000) and higher unemployment rates (Algan et al., 2017). The current study adds to these consequences a potential threat to vital blood collection efforts. According to the most recent evidence, blood donation rates in Europe have decreased since 2014 when our data was collected (WHO, 2021), which highlights that threats to the blood supply remain a pressing challenge. Without a sufficient supply of blood, heart surgery patients, victims of serious trauma, and chemotherapy patients will not be able to receive the medical care they urgently need. Hence, building and maintaining trust in the healthcare system should be a priority for governments and organizations in order to ensure a continuous supply of blood. More generally, it is important to recognize that (changes in) people’s perception of the healthcare system, including public trust, can have significant ramifications on different kinds of behaviors that take place in these medical settings, such as blood donation.

## Supplemental Material

sj-csv-1-hpq-10.1177_13591053231175809 – Supplemental material for How public trust and healthcare quality relate to blood donation behavior: Cross-cultural evidence Click here for additional data file. Supplemental material, sj-csv-1-hpq-10.1177_13591053231175809 for How public trust and healthcare quality relate to blood donation behavior: Cross-cultural evidence by Caroline Graf, Bianca Suanet, Pamala Wiepking and Eva-Maria Merz in Journal of Health Psychology

sj-docx-2-hpq-10.1177_13591053231175809 – Supplemental material for How public trust and healthcare quality relate to blood donation behavior: Cross-cultural evidence Click here for additional data file. Supplemental material, sj-docx-2-hpq-10.1177_13591053231175809 for How public trust and healthcare quality relate to blood donation behavior: Cross-cultural evidence by Caroline Graf, Bianca Suanet, Pamala Wiepking and Eva-Maria Merz in Journal of Health Psychology

sj-pdf-3-hpq-10.1177_13591053231175809 – Supplemental material for How public trust and healthcare quality relate to blood donation behavior: Cross-cultural evidence Click here for additional data file. Supplemental material, sj-pdf-3-hpq-10.1177_13591053231175809 for How public trust and healthcare quality relate to blood donation behavior: Cross-cultural evidence by Caroline Graf, Bianca Suanet, Pamala Wiepking and Eva-Maria Merz in Journal of Health Psychology

sj-pdf-4-hpq-10.1177_13591053231175809 – Supplemental material for How public trust and healthcare quality relate to blood donation behavior: Cross-cultural evidence Click here for additional data file. Supplemental material, sj-pdf-4-hpq-10.1177_13591053231175809 for How public trust and healthcare quality relate to blood donation behavior: Cross-cultural evidence by Caroline Graf, Bianca Suanet, Pamala Wiepking and Eva-Maria Merz in Journal of Health Psychology

sj-pdf-5-hpq-10.1177_13591053231175809 – Supplemental material for How public trust and healthcare quality relate to blood donation behavior: Cross-cultural evidence Click here for additional data file. Supplemental material, sj-pdf-5-hpq-10.1177_13591053231175809 for How public trust and healthcare quality relate to blood donation behavior: Cross-cultural evidence by Caroline Graf, Bianca Suanet, Pamala Wiepking and Eva-Maria Merz in Journal of Health Psychology

sj-pdf-6-hpq-10.1177_13591053231175809 – Supplemental material for How public trust and healthcare quality relate to blood donation behavior: Cross-cultural evidence Click here for additional data file. Supplemental material, sj-pdf-6-hpq-10.1177_13591053231175809 for How public trust and healthcare quality relate to blood donation behavior: Cross-cultural evidence by Caroline Graf, Bianca Suanet, Pamala Wiepking and Eva-Maria Merz in Journal of Health Psychology This article is distributed under the terms of the Creative Commons Attribution 4.0 License ( http://www.creativecommons.org/licenses/by/4.0/) which permits any use, reproduction and distribution of the work without further permission provided the original work is attributed as specified on the SAGE and Open Access pages ( https://us.sagepub.com/en-us/nam/open-access-at-sage).
