# Supplementary material for: How public trust and healthcare quality relate to blood donation behavior: Cross-cultural evidence
Source: J Health Psychol. 2023 Jun 5;29(1):3–14. doi: 10.1177/13591053231175809 (PMC10757392; doi:10.1177/13591053231175809)
Supplement: sj-docx-2-hpq-10.1177_13591053231175809 – Supplemental material for How public trust and healthcare quality relate to blood donation behavior: Cross-cultural evidence [file sj-docx-2-hpq-10.1177_13591053231175809.docx]

Data sharing and replication instructions: Explanatory note

This note accompanies the manuscript entitled *How Public Trust and Healthcare Quality Relate to Blood Donation Behavior: Cross-Cultural Evidence* by [details removed for peer review].

Our analyses can be reproduced by running the script *P2_analysis.R* in the statistical software R (R version 4.0.2). The file *P2_analysis.R* includes the analysis code (syntax) for all main and supplementary analyses reported in the main text and appendix of the manuscript. The statistical software R does not produce external log files; Output (including all figures and all statistical results) are produced by running *P2_analysis.R* within R.

We make use of several secondary data sources that need to be downloaded and saved in the folder data/ before running *P2_analysis.R*: the Eurobarometer [1], European Values Survey [2], Healthcare Access and Quality index [3], and the World Health Organization Global Health Expenditure database [4].

**Instructions on accessing secondary datasets**

1. Eurobarometer 82.2

- Instructions: Download file ZA5931_v3-0-0.dta from https://doi.org/10.4232/1.12999 (free account with GESIS required)

1. Global Burden of Diseases Collaborative: Healthcare Access and Quality Index

- Instructions: Download folder IHME_GBD_2015_HAQ_INDEX_1990_2015 from http://ghdx.healthdata.org/record/ihme-data/gbd-2015-healthcare-access-and-quality-index-1990-2015 (click on 'files'; free)

1. World Health Organization Global Health Expenditure database

- Instructions: Download file total-healthcare-expenditure-as-share-of-national-gdp-by-country.csv from Our World in Data: https://ourworldindata.org/grapher/total-healthcare-expenditure-as-share-of-national-gdp-by-country (select 'Download' and then 'full data (CSV)'; free)

1. European Values Survey: EVS (2021)

- Instructions: Download zip folder ZA7503_v2-0-0.dta.zip from https://doi.org/10.4232/1.13736 (free account with GESIS required)

**References**

[1] European Commission. Eurobarometer 82.2 (2014). TNS opinion [producer]. Cologne, Germany: GESIS Data Archive; 2018.

[2] European Values Survey: EVS (2021): EVS Trend File 1981-2017. GESIS Data Archive, Cologne. ZA7503 Data file Version 2.0.0, https://doi.org/10.4232/1.13736.

[3] Global Burden of Diseases Collaborative: Healthcare Access and Quality Index: Fullman, N., Yearwood, J., Abay, S. M., Abbafati, C., Abd-Allah, F., Abdela, J., ... & Chang, H. Y. (2018). Measuring performance on the Healthcare Access and Quality Index for 195 countries and territories and selected subnational locations: a systematic analysis from the Global Burden of Disease Study 2016. The Lancet, 391(10136), 2236-2271.

[4] World Health Organization Global Health Expenditure database: World Health Organization. (2015). Global Health Expenditure Database (GHED). World Health Organization.
