# Supplementary material for: How public trust and healthcare quality relate to blood donation behavior: Cross-cultural evidence
Source: J Health Psychol. 2023 Jun 5;29(1):3–14. doi: 10.1177/13591053231175809 (PMC10757392; doi:10.1177/13591053231175809)
Supplement: sj-pdf-3-hpq-10.1177_13591053231175809 – Supplemental material for How public trust and healthcare quality relate to blood donation behavior: Cross-cultural evidence [file sj-pdf-3-hpq-10.1177_13591053231175809.pdf]

Appendix

This appendix accompanies the paper entitled *How Public Trust and Healthcare Quality Relate to Blood Donation Behavior: Cross-Cultural Evidence* by [details removed for peer review].

Supplementary analyses: Robustness checks

Supplementary methods

In order to test for the robustness of our findings, we performed multiple checks. First, we checked the robustness of results when running a full model including all three country-level indicators. To better interpret the results of this model, we tested for multicollinearity by checking if there is a relationship between country-level quality of healthcare and public trust in the healthcare system. Next, we examined whether the results were driven by any single country. To this end we excluded one of the 28 countries in each model specification. Lastly, we tested whether our temporal matching strategy (i.e., matching of the approximate donation time point with time-specific country-level indicators) affected the results. To examine this, we ran an additional analysis on the subset of respondents who are relatively young. If these individuals have donated, they will have done this relatively recently. For these respondents we can be more certain that the country-level indicators capture the state of quality of healthcare and public trust at the time of donation. We constructed this subset by taking the youngest respondents such that country level sample sizes amounted to at least N = 250. In addition to the extra analyses on young respondents, we also conducted the main analyses using country-level indicators that were aggregated across years.

Supplementary results

As a first robustness check, we implemented one full model which included all three country-level indicators. The results of this analysis mirrored the results of the separate models for public trust and healthcare quality. While trust in the healthcare system had a significant positive effect on blood donation behavior ($b = 0.120$, $p < 0.001$; Model 4 in Table S2), HAQ index and healthcare expenditures were not statistically significantly associated with having donated blood (HAQ: $b = 0.083$, $p = 0.154$; healthcare expenditures: $b = -0.051$, $p = 0.226$; Model 4 in Table S2). However, we also found that our country-level indicators are correlated, which raises issues of multicollinearity in the full model. More specifically, public trust and HAQ index were significantly correlated (Pearson's $r = 0.48$, $p < 0.05$), and HAQ index and healthcare expenditures were also strongly correlated (Pearson's $r = 0.69$, $p < 0.001$). Public trust and healthcare expenditures were not significantly correlated (Pearson's $r = 0.29$, $p = 0.137$). Given the strong correlation between HAQ index and healthcare expenditures, we implemented an additional robustness check in which separate models were run for HAQ index and healthcare expenditures. The results of these two models were similar to the results reported for the combined model (HAQ: $b = 0.094$, $p = 0.088$; healthcare expenditures: $b = -0.021$, $p = 0.609$).

Next, we conducted additional robustness checks in which we excluded one of the 28 countries in each specification. Our main results remained robust for trust in the healthcare system and healthcare expenditures. However, in six specifications there was now a significant positive effect of HAQ index (see Table S3; excluded countries: Italy, Latvia, Lithuania, Malta, Slovenia, or Sweden). That is, excluding one of these six countries had as a result that higher healthcare quality in terms of HAQ index was associated with a higher likelihood to have donated blood.

We also conducted a robustness check on exclusively young respondents by including only respondents 43 years or younger. This cutoff resulted in all country-level sample sizes being larger than 250 (except for three small countries: Malta (n = 149), Cyprus (n = 183) and Luxembourg (n = 187)). Models were hence run on N_{young} = 9,421, which corresponds to 36% of the original sample size. The summary statistics (see Table S4) illustrate that individuals in this subsample are less likely to have donated blood, more educated, more likely to be employed and have children in the household, and more likely to live in a city than in a rural area, compared to the full sample. Moreover, country-level trust in the healthcare system is *lower*, whereas HAQ index and healthcare expenditures are *higher*, in this subsample compared to the full sample. Despite these differences, the overall distribution of the three indicators across countries appears to be similar to the indicator distributions in the full sample (see Fig. S10). The models for this subsample however revealed no significant effect of trust in the healthcare system, HAQ index or healthcare expenditures on blood donation behavior (see Table S5). This indicates that the country-level indicators are not associated with donating blood when examining a smaller subsample of young respondents.

Lastly, we aggregated the country-level indicators across years (i.e., taking the average over years) and performed main models M2 and M3 using these indicators. The results are presented in Table S6. Neither trust in the healthcare system nor the healthcare quality indicators were statistically significantly associated with blood donation behavior. This suggests that our main findings are at least partly driven by heterogeneity of trust and healthcare quality over time.

Supplementary discussion

In addition to our main results, we also gained further insights from several robustness checks. A first set of robustness checks provided some evidence that healthcare quality in terms of HAQ index may after all be associated with blood donation behavior. That is, in six model specifications that excluded respondents from Italy, Latvia, Lithuania, Malta, Slovenia, or Sweden, respectively, there was actually a statistically significant *positive* effect of HAQ index on propensity to donate blood. Given that the HAQ index does not reach significance in our main model, we interpret this as mixed evidence for a relationship between HAQ index and blood donation.

Further sensitivity checks examined the subset of young respondents. Within these analyses, none of the three country level indicators predicted blood donation. The null effect of public trust was particularly unexpected given the main model results. A possible explanation for this may be that the sample size was greatly reduced in these models and that young respondents were overall less likely to have donated blood. There may also be generational differences, such that young people donate less overall, or are less influenced by trust. The relationship between trust and blood donation may also be non-linear (e.g., there may be a trust threshold that must be reached for it to affect blood donation behavior). We also found that country-level indicator values averaged across time are not predictive of donating blood. This suggests that the temporal variation of trust in the healthcare system plays an important role for the observed relationship between public trust and blood donation behavior. Although our strategy of indicator matching involves multiple assumptions, we found it important to account for temporal variation in trust and healthcare quality and hence included time-specific measures when conceiving the study (as documented in our preregistration). That is, we did not anticipate that there may be systematic variation across time when designing the analysis plan, but we think that the existence of temporal variation and its role for blood donation is an interesting finding in itself, which we deem promising for further research to investigate further.

Supplementary figures and tables

Table S 1: Dependent and independent variables employed in the analyses. ▷ denotes source: Eurobarometer; other sources in brackets.

Variable	Description	Original item	Response range
Blood donation	Individual-level response: Blood donation during lifetime ▷	During the lifetime of a person it is possible to donate different body substances (blood or cells) to help other people. Could you please indicate which ones you have [...] donate[d] yourself? Blood	0; 1 (dummy coded) 0 (not donated blood in the past) 1 (donated blood in the past)
Healthcare quality	Country-level variable: Healthcare expenditures per GDP (in percent; World Health Organization Global Health Expenditure database)	NA	numeric (theoretical range: 0 - 100 percent)
	Country-level variable: Healthcare Access and Quality Index (HAQ index; Global Burden of Diseases Collaborative)	NA	numeric (theoretical range: 0 - 100)

Public trust in the healthcare system	Country-level variable: Aggregated response of level of confidence in the healthcare system (European Values Survey)	How much confidence do you have in the healthcare system?	$1.0 - 4.0$<br><br>Aggregation of individual-level responses with response range:<br><br>1 (none at all)<br><br>2 (not very much)<br><br>3 (quite a lot)<br><br>4 (a great deal)
Controls	Age of respondent ▷	How old are you?	numeric
	Gender ▷	NA	$0; 1$ (dummy coded)<br><br>0 (Male)<br><br>1 (Female)
	Education (Age when finished full-time education) ▷	How old were you when you stopped full-time education?	numeric
	Partner status (live with partner or not) ▷	Could you give me the letter which corresponds best to your own current situation?<br><br>(recoded from 14 levels, e.g., married/remarried and living with/without children)	$0; 1$ (dummy coded)<br><br>0 (Living without partner)<br><br>1 (Living with partner)
	Employment status ▷	What is your current occupation?	$0; 1$ (dummy coded)<br><br>0 (unemployed)<br><br>1 (employed)

Parental status (operationalized as children in household) ▷	NA	0; 1 (dummy coded)<br>0 (Living without children)<br>1 (Living with children)
Type of community ▷	Would you say you live in a rural area or village, small or middle sized town, or a large town?	0; 1; 2 (dummy coded)<br>0 (large town)<br>1 (small or middle sized town)<br>2 (rural area or village)

Table S 2: Descriptives of indicators at the country-level, averaged across available years.

	Trust in the healthcare system		HAQ index		Healthcare expenditure	
Country	Mean	Std.	Mean	Std.	Mean	Std.
Austria	3.06	0.12	79.64	4.60	10.03	0.56
Belgium	3.06	0.10	79.69	4.19	8.28	0.95
Bulgaria	2.10	0.16	64.79	2.10	5.71	1.16
Croatia	2.33	0.02	73.99	4.13	7.08	0.59
Cyprus	2.66	0.00	76.23	4.16	5.68	0.96
Czech Republic	2.41	0.05	75.99	5.20	6.81	0.37
Denmark	2.80	0.04	78.63	2.61	8.74	0.95
Estonia	2.62	0.07	68.08	4.85	6.02	0.55
Finland	2.97	0.10	79.49	4.53	8.00	0.53
France	2.91	0.01	78.75	4.57	10.29	0.42
Germany	2.49	0.17	78.26	4.70	9.97	0.59
Greece	2.09	0.11	81.19	3.40	8.50	0.56
Hungary	2.24	0.05	69.4	4.47	7.40	0.37
Ireland	2.48	0.27	80.15	3.95	6.94	0.83
Italy	2.35	0.12	81.57	4.15	7.88	0.79
Latvia	2.62	0.11	69.52	4.17	6.20	0.37
Lithuania	2.23	0.02	68.45	3.27	5.86	0.59
Luxembourg	3.03	0.12	80.94	4.63	6.63	1.09
Malta	3.18	0.03	77.78	3.21	6.77	1.41
Netherlands	2.88	0.05	81.75	2.98	8.01	0.99
Poland	2.54	0.16	69.59	5.68	5.93	0.54
Portugal	2.38	0.09	74	5.90	8.47	1.13
Romania	2.62	0.06	63.69	5.05	4.21	0.99
Slovakia	2.49	0.03	69.97	3.09	6.45	0.88
Slovenia	2.79	0.05	75.1	4.86	7.91	0.61
Spain	2.80	0.11	79.91	5.20	7.83	0.70
Sweden	2.88	0.01	83.47	3.12	8.44	0.81
United Kingdom	2.76	0.18	77.79	3.40	7.45	1.05

Note: All countries have data available for all years, except Cyprus for public trust in 1999. Sources: European Values Survey (EVS, 2021, available years: 1999, 2008); Healthcare access and quality index (Global Burden of Diseases Collaborative; Fullman et al., 2018, available years: 1990, 1995, 2000, 2005, 2010, 2015); World Health Organization Global Health Expenditure database (World Health Organization, 2015, available years: 1995- 2014).

Table S 3: Full multilevel model results.

		Main models			Additional models	
		(1)	(2)	(3)	(4)	(5)
Individual-level	(Intercept)	-0.439^{***}	-0.478^{***}	-0.615^{***}	-0.611^{***}	-0.438^{***}
		(0.069)	(0.137)	(0.221)	(0.205)	(0.080)
	Age (years) ▷	0.216^{***}	0.094^{***}	-0.113^{*}	-0.109^{*}	0.097^{***}
		(0.016)	(0.024)	(0.060)	(0.060)	(0.033)
	Gender (female = 1)	-0.449^{***}	-0.456^{***}	-0.464^{***}	-0.464^{***}	-0.452^{***}
		(0.027)	(0.027)	(0.027)	(0.027)	(0.027)
	Education ▷	0.182^{***}	0.178^{***}	0.174^{***}	0.172^{***}	0.179^{***}
		(0.015)	(0.015)	(0.015)	(0.015)	(0.015)
	Living with partner	0.111^{***}	0.092^{***}	0.059^{**}	0.062^{**}	0.095^{***}
		(0.029)	(0.029)	(0.030)	(0.030)	(0.029)
	Employed	0.299^{***}	0.251^{***}	0.218^{***}	0.219^{***}	0.280^{***}
		(0.030)	(0.031)	(0.034)	(0.034)	(0.030)
	Children in household	0.035	0.087^{**}	0.082^{**}	0.084^{**}	0.055
		(0.037)	(0.038)	(0.041)	(0.041)	(0.038)
	Type of community					
	Large town (ref.)	-	-	-	-	-
		-	-	-	-	-
	Mid-sized town	−0.023	−0.027	−0.027	−0.029	−0.024
		(0.033)	(0.033)	(0.033)	(0.033)	(0.033)
	Rural area	−0.054	-0.061^{*}	-0.064^{*}	-0.066^{*}	-0.059^{*}
		(0.035)	(0.035)	(0.035)	(0.035)	(0.035)
Country-level	Trust in healthcare system ▷		0.114^{***}		0.120^{***}	0.165^{***}
			(0.033)		(0.035)	(0.034)
	HAQ index ▷			0.101	0.083	-0.114^{**}
				(0.056)	(0.058)	(0.048)
	Healthcare expenditures ▷			−0.020	−0.051	-0.124^{***}
				(0.041)	(0.042)	(0.041)
	Country random intercept	Yes	Yes	Yes	Yes	Yes
	Survey wave random intercept	No	Yes	Yes	Yes	No
	Observations	26,019	26,019	26,019	26,019	26,019

Note: Standard errors in brackets; *** denotes p<0.001; ** denotes p<0.01; * denotes p<0.05; ▷ indicates variable was normalized. Dependent variable is blood donation. All models control for sociodemographic factors. Main model M2 further includes trust in the healthcare system; M3 includes the two healthcare quality indicators. Additional models M4-M5 include trust in the healthcare system and the two healthcare quality indicators simultaneously. M4 includes survey wave random effects, whereas M5 does not.

Table S 4: Coefficients of HAQ index for model specifications excluding individual countries (only specifications where HAQ index had a significant effect on blood donation are listed). Standard errors in brackets; * denotes p<0.05.

Excluded country	HAQ index
Italy	0.112*
	(0.056)
Latvia	0.121*
	(0.059)
Lithuania	0.135*
	(0.060)
Malta	0.126*
	(0.058)
Slovenia	0.116*
	(0.057)
Sweden	0.126*
	(0.057)

Table S 5: Descriptive statistics for the dependent and independent variables for the subsample of young respondents (age <44).

	Variable	Range	Mean	Std.	N
Individual-level	Blood donation	0; 1	0.34		9640
	Gender (female = 1)	0; 1	0.56		9640
	Age (years)	18; 43	31.82	7.37	9640
	Education	0; 43	20.26	4.14	9452
	Living with partner	0; 1	0.65		9609
	Employed	0; 1	0.68		9640
	Children in household	0; 1	0.42		9638
	Type of community				
	Large town (ref.)	0; 1	0.30		9635
	Mid-sized town	0; 1	0.42		9635
	Rural area	0; 1	0.28		9635
Country-level	Trust in healthcare system	1.84; 3.23	2.57	0.33	9640
	HAQ index	64.2; 90.5	79.91	6.07	9640
	Healthcare expenditures	4.57; 11.97	8.19	1.62	9640

Sources: Eurobarometer (European Commission, 2018); European Values Survey (EVS, 2021); Healthcare access and quality index (Global Burden of Diseases Collaborative; Fullman et al., 2018); World Health Organization Global Health Expenditure database (World Health Organization, 2015).

Table S 6: Model results for subsample of young respondents (age<44). Y1 and Y2 are analogous to main models 2 and 3 (see Table S2).

		(Y1)	(Y2)
Individual-level	(Intercept)	-0.411^{***}	-0.341^{**}
		(0.156)	(0.159)
	Age (years) ▷	0.360^{***}	0.488^{***}
		(0.116)	(0.107)
	Gender (female = 1)	-0.304^{***}	-0.308^{***}
		(0.046)	(0.046)
	Education ▷	0.287^{***}	0.281^{***}
		(0.032)	(0.032)
	Living with partner	0.067	0.063
		(0.055)	(0.055)
	Employed	0.268^{***}	0.256^{***}
		(0.055)	(0.055)
	Children in household	0.067	0.081
		(0.053)	(0.051)
	Type of community		
	Large town (ref.)	-	-
		-	-
	Mid-sized town	0.007	0.006
		(0.055)	(0.055)
	Rural area	−0.045	−0.045
		(0.060)	(0.060)
Country-level	Trust in healthcare system ▷	0.025	
		(0.047)	
	HAQ index ▷		0.062
			(0.090)
	Healthcare expenditures ▷		0.048
			(0.068)
	Country random intercept	Yes	Yes
	Survey wave random intercept	Yes	Yes
	Observations	9,421	9,421

Note: Standard errors in brackets; *** denotes p<0.001; ** denotes p<0.01; * denotes p<0.05; ▷ indicates variable was normalized. Dependent variable is blood donation. All models control for sociodemographic factors. Y1 further includes trust in the healthcare system; Y2 includes the two healthcare quality indicators.

Table S 7: Model results for analysis with independent variables (i.e., trust in the healthcare system, HAQ index, healthcare expenditures) aggregated over time. A1 and A2 are analogous to main models 2 and 3 (see Table S2).

		(A1)	(A2)
Individual-level	(Intercept)	-0.476^{***}	-0.584^{**}
		(0.143)	(0.195)
	Age (years) ▷	0.093^{***}	-0.107
		(0.024)	(0.060)
	Gender (female = 1)	-0.456^{***}	-0.464^{***}
		(0.027)	(0.027)
	Education ▷	0.180^{***}	0.175^{***}
		(0.015)	(0.015)
	Living with partner	0.089^{**}	0.059^{*}
		(0.029)	(0.030)
	Employed	0.250^{***}	0.219^{***}
		(0.031)	(0.034)
	Children in household	0.086^{*}	0.082^{*}
		(0.038)	(0.041)
	Type of community		
	Large town (ref.)	-	-
		-	-
	Mid-sized town	-0.025	-0.027
		(0.033)	(0.033)
	Rural area	-0.059	-0.063
		(0.035)	(0.035)
Country-level	Trust in healthcare system ▷	0.036	
		(0.059)	
	HAQ index ▷		0.020
			(0.073)
	Healthcare expenditures ▷		0.026
			(0.071)
	Country random intercept	Yes	Yes
	Survey wave random intercept	Yes	Yes
	Observations	26,019	26,019

Note: Standard errors in brackets; *** denotes p<0.001; ** denotes p<0.01; * denotes p<0.05; ▷ indicates variable was normalized. Dependent variable is blood donation. All models control for sociodemographic factors. A1 further includes trust in the healthcare system; A2 includes the two healthcare quality indicators.

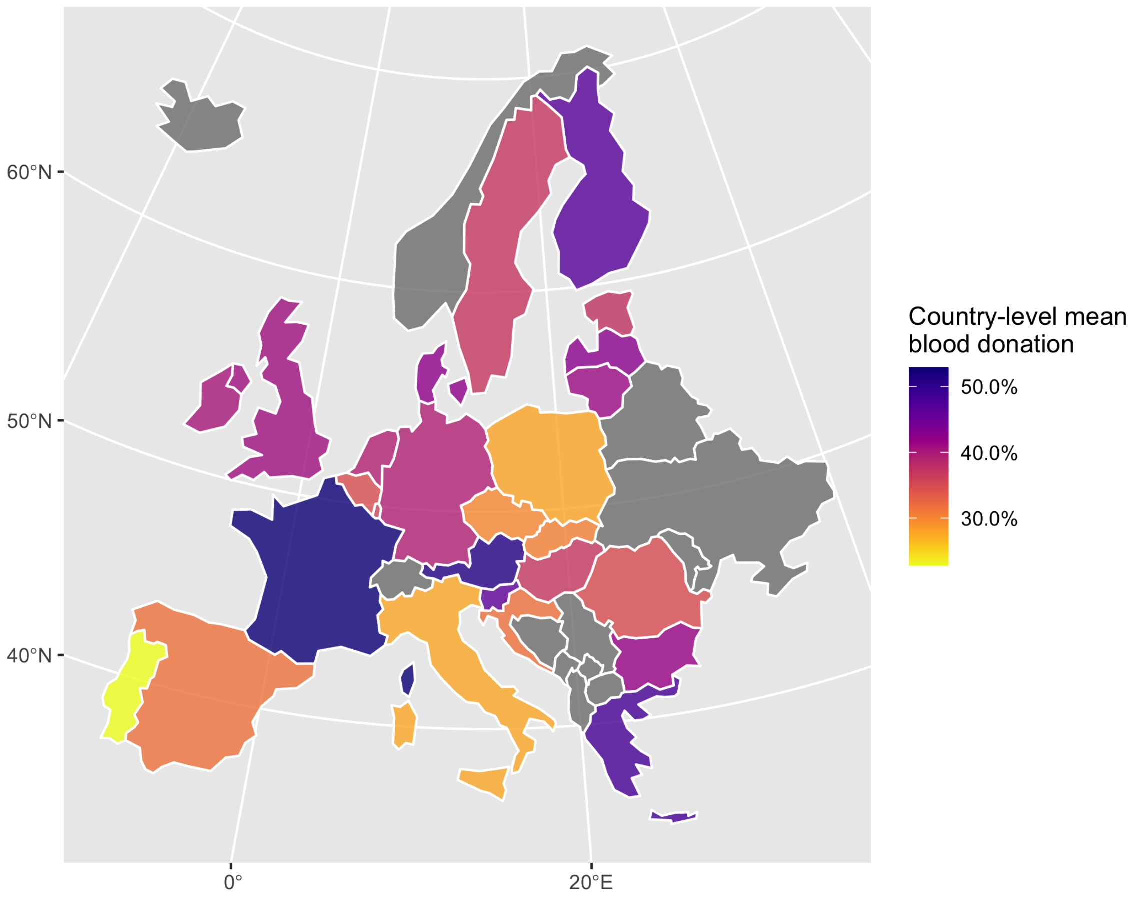

Figure S 1: Map of the proportion of individuals within a country who have donated blood (in percent).

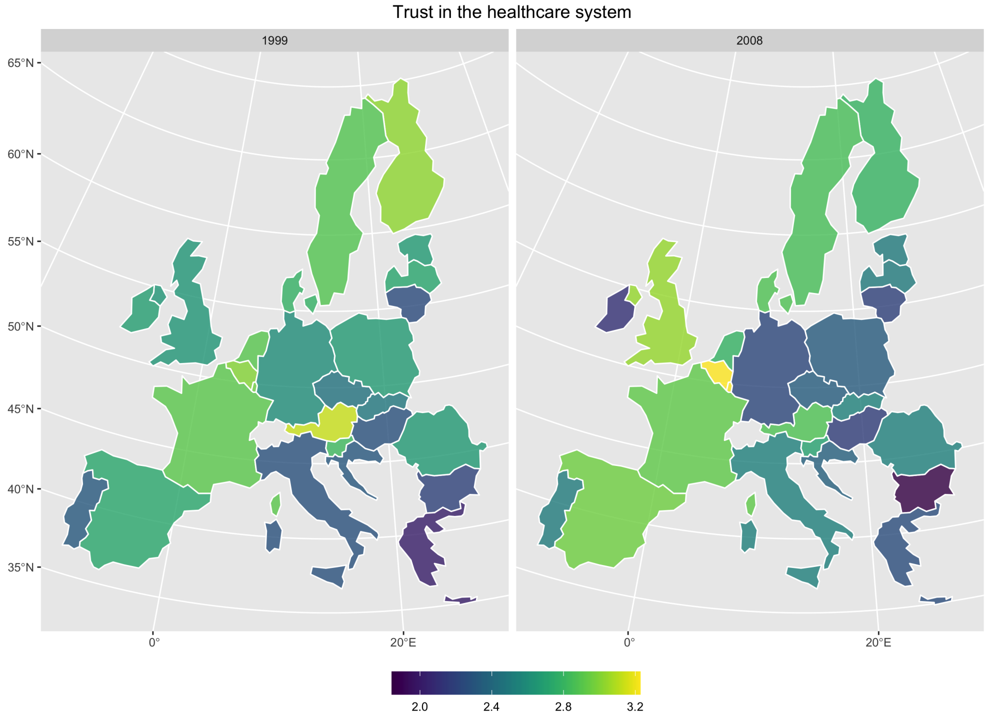

Figure S 2: Maps of trust in the healthcare system over time. There are two time points for which data is available (1999; 2008).

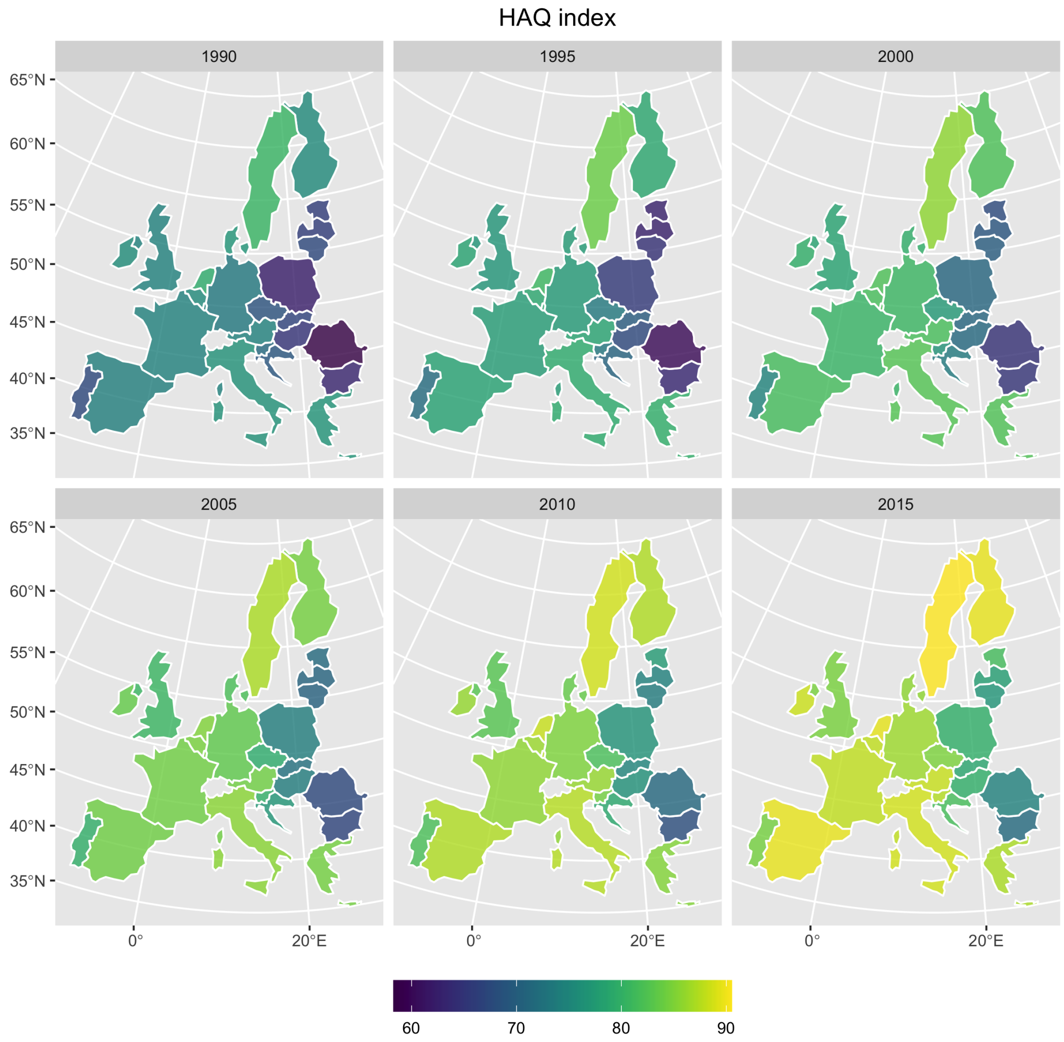

Figure S 3: Maps of healthcare quality in terms of HAQ index over time. There are six time points for which data is available (1990; 1995; 2000; 2005; 2010; 2015).

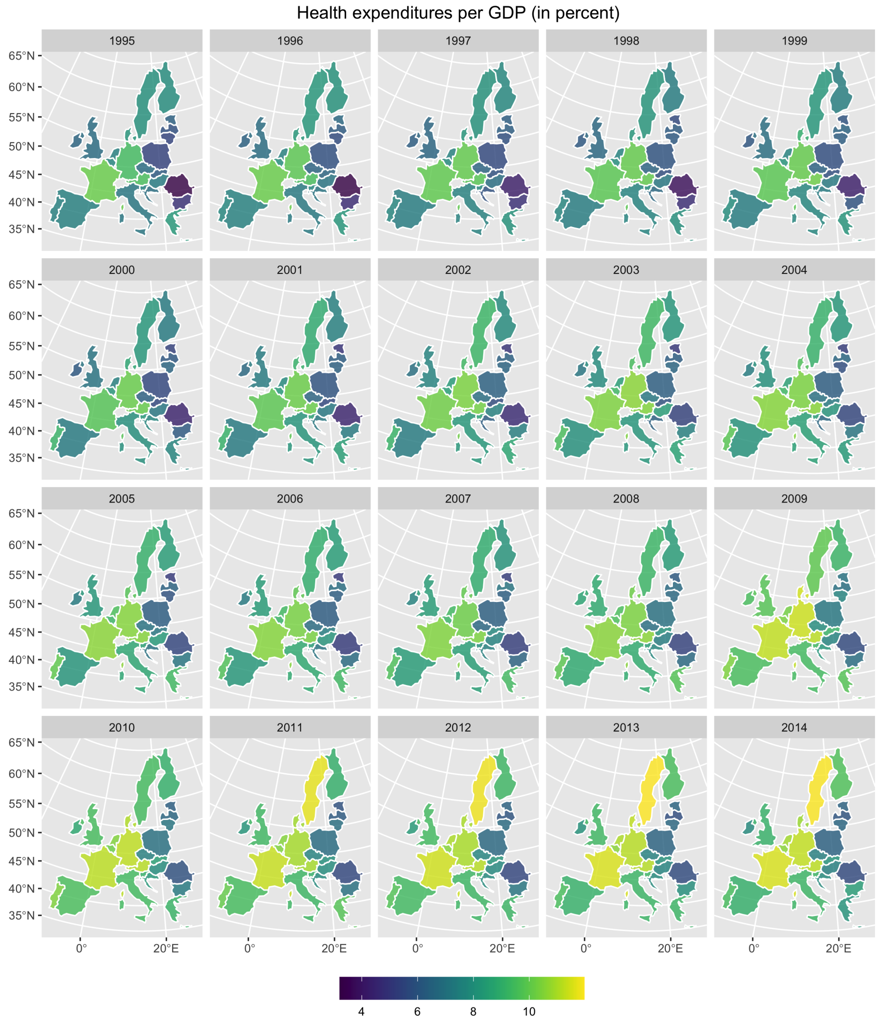

Figure S 4: Maps of healthcare quality in terms of healthcare expenditures per GDP (in percent) over time. There are 20 time points for which data is available (1995 - 2014, yearly).

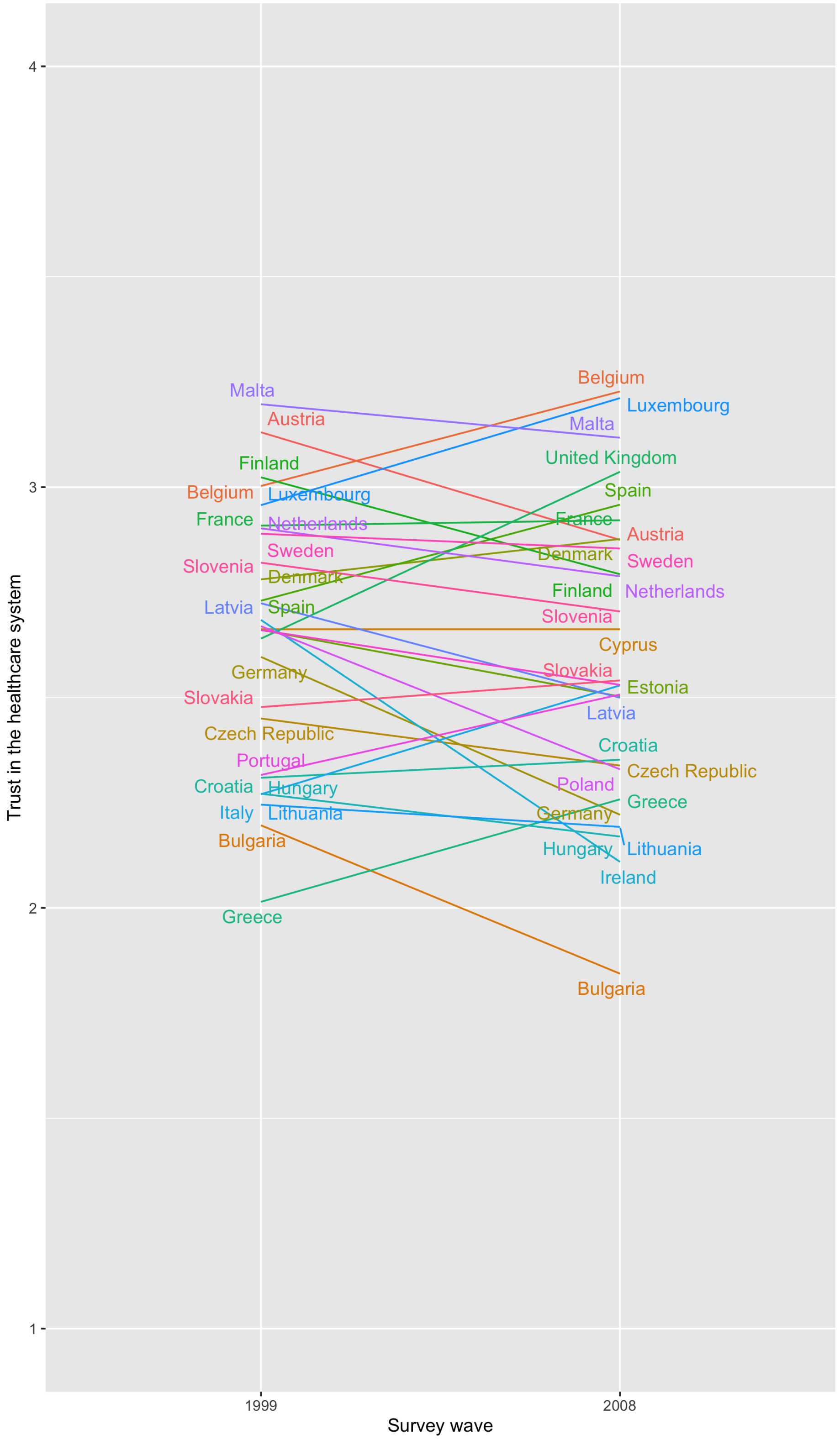

Figure S 5: Line chart displaying country-level trust in the healthcare system across the two survey waves.

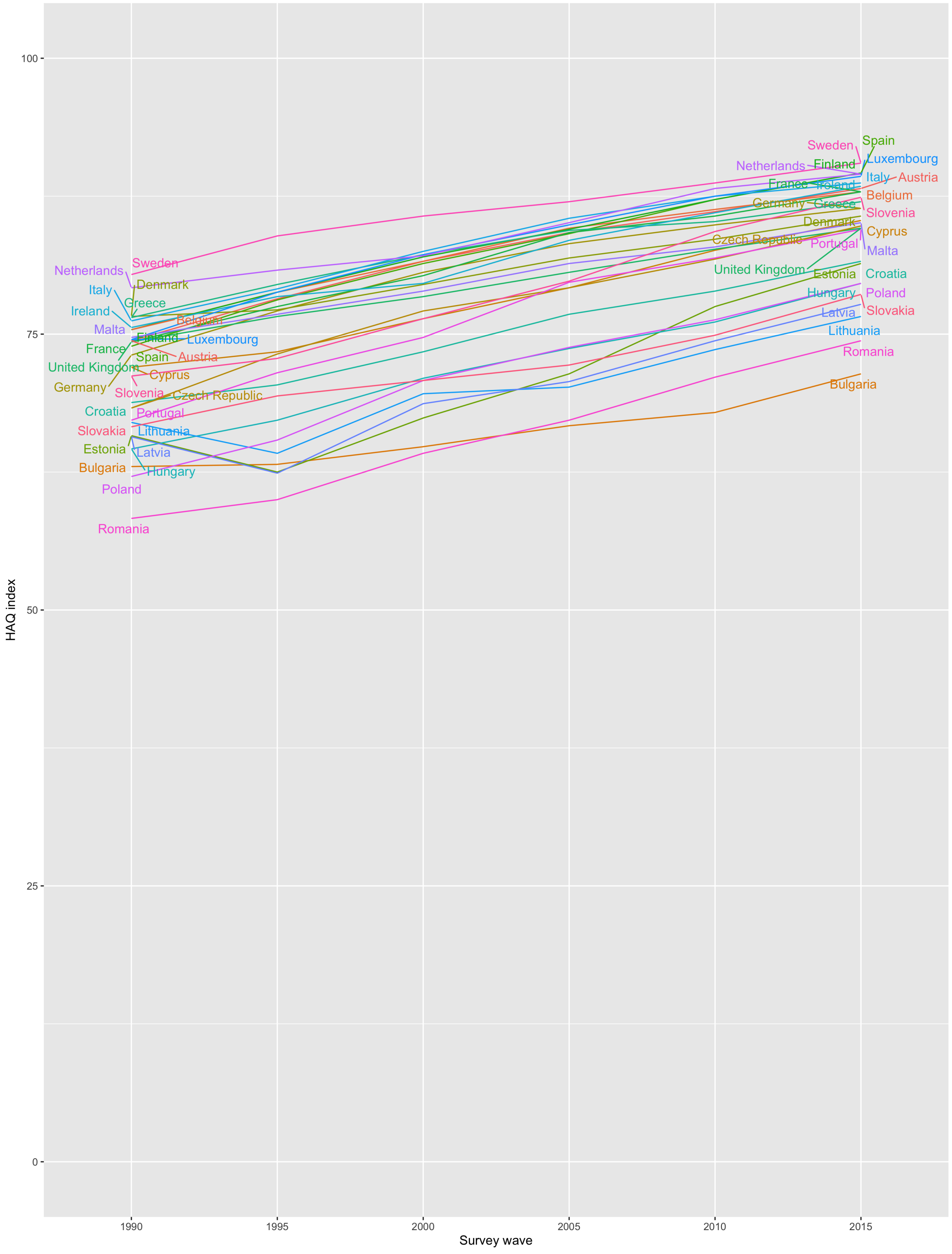

Figure S 6: Line chart displaying country-level scores of the HAQ index across the six survey waves.

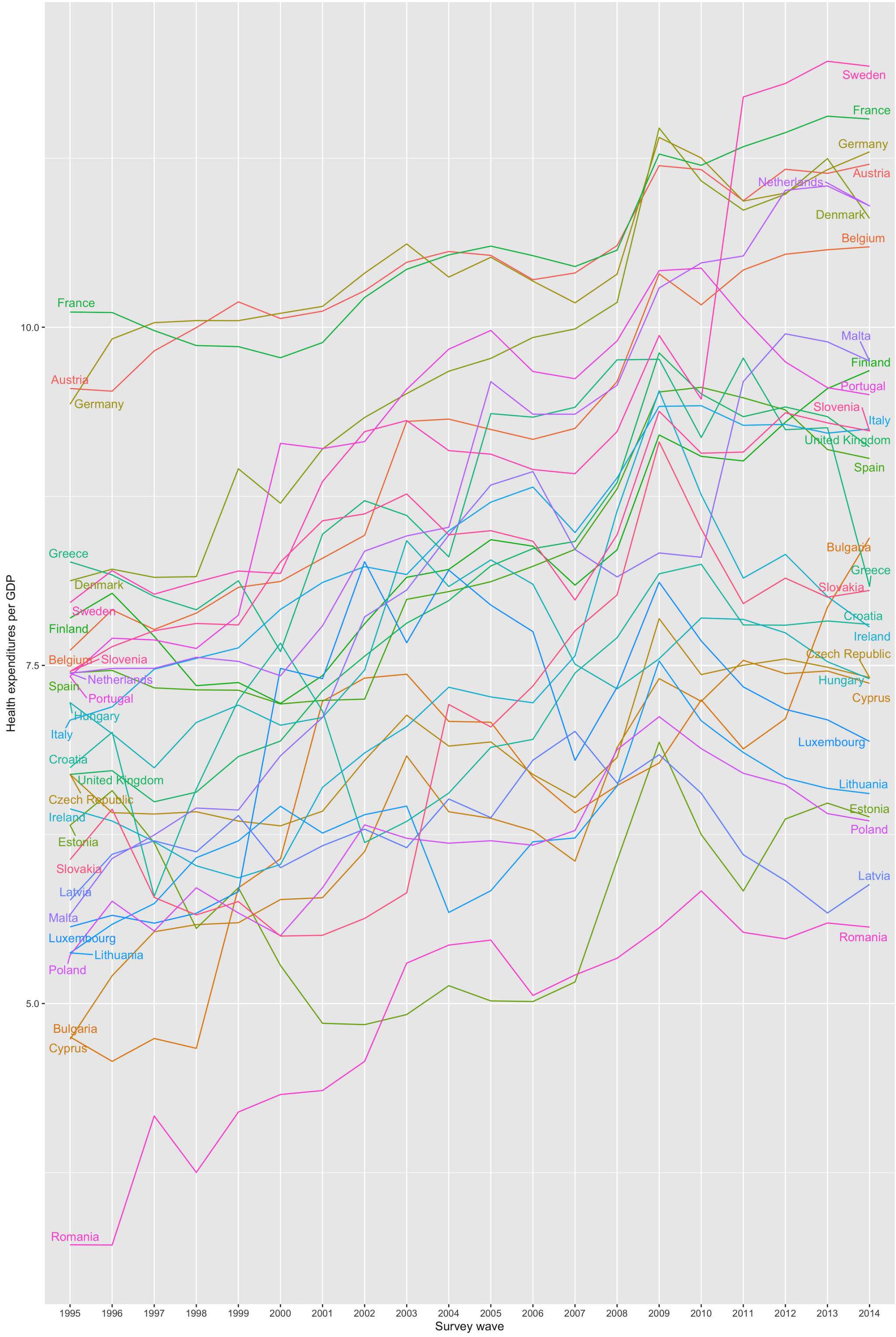

Figure S 7: Line chart displaying country-level healthcare expenditures per GDP across the 20 survey waves.

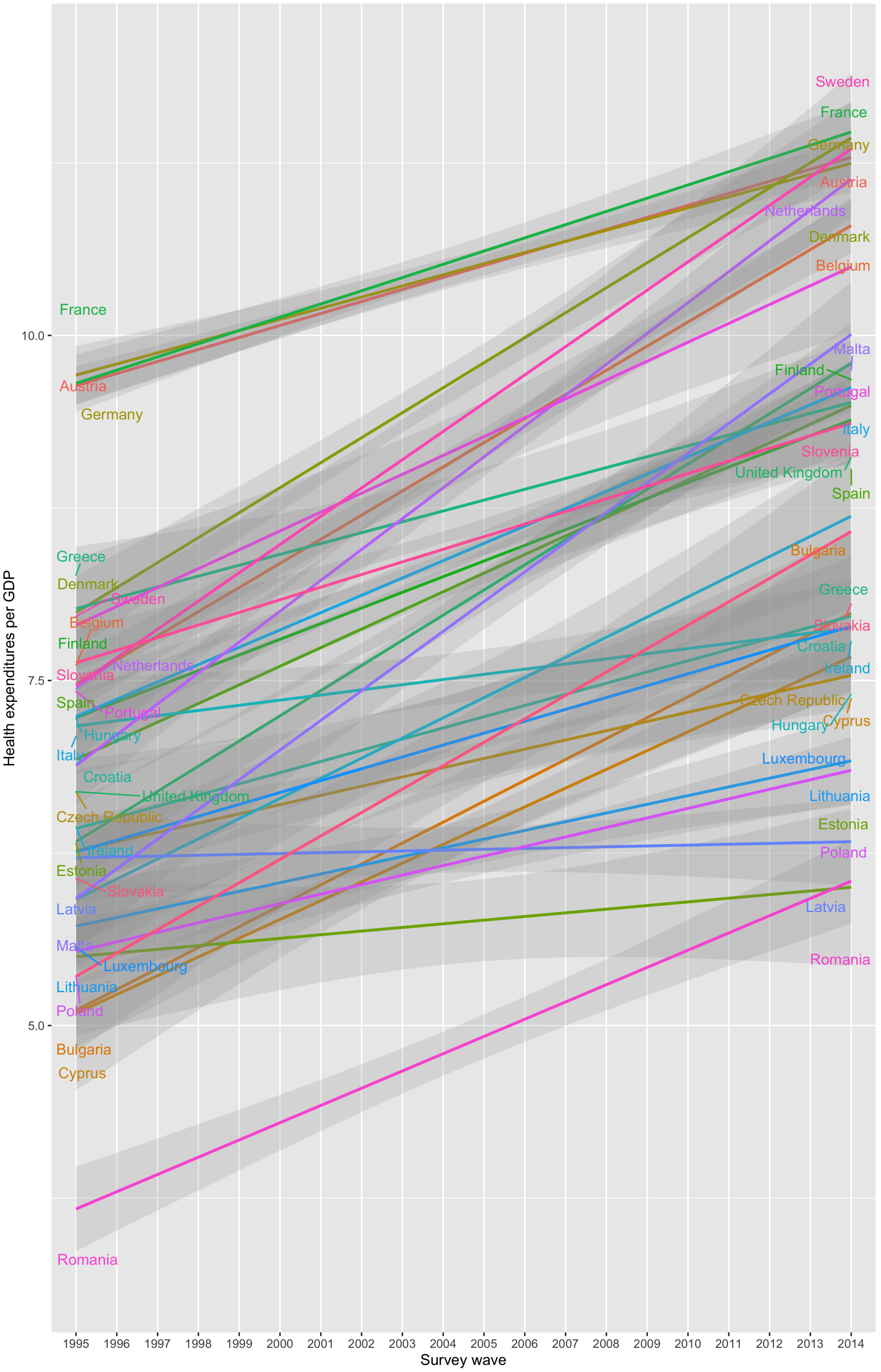

Figure S 8: Line chart displaying country-level healthcare expenditures per GDP at different survey time points. Straight lines are obtained by applying Lowess smoothing.

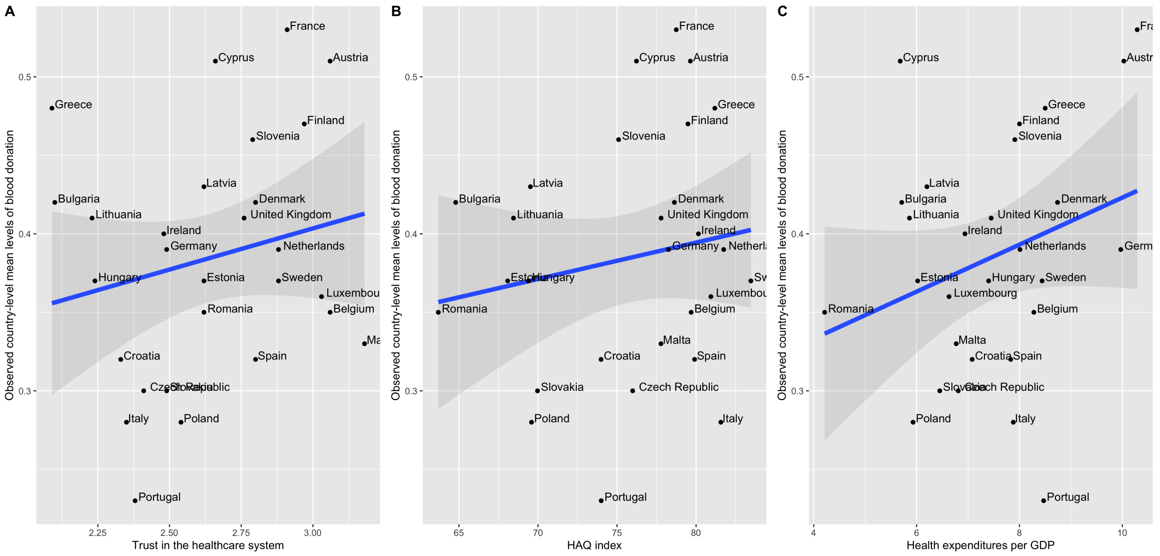

Figure S 9: Country-level mean levels of blood donation plotted against country-level mean indicator values. (A) Trust in the healthcare system. (B) Healthcare quality as measured by the HAQ index. (C) Healthcare quality as measured by healthcare expenditures per GDP.

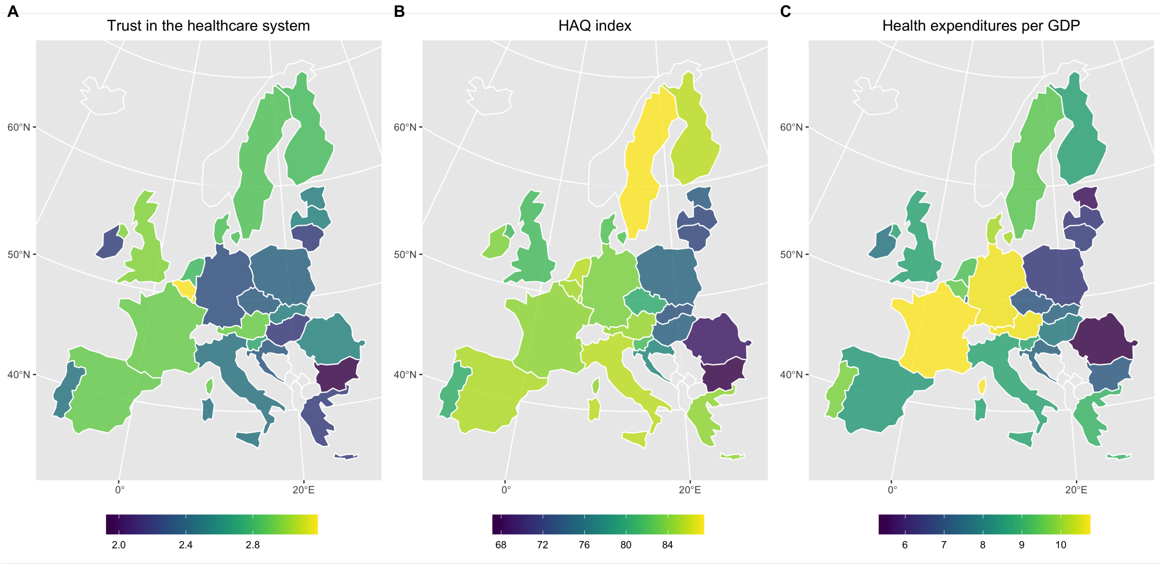

Figure S 10: Maps displaying trust in the healthcare system and quality of healthcare across Europe for the subsample of young respondents (age <44). (A) Trust in the healthcare system. (B) Healthcare quality as measured by the HAQ index. (C) Healthcare quality as measured by healthcare expenditures per GDP.
