# Supplementary material for: How public trust and healthcare quality relate to blood donation behavior: Cross-cultural evidence
Source: J Health Psychol. 2023 Jun 5;29(1):3–14. doi: 10.1177/13591053231175809 (PMC10757392; doi:10.1177/13591053231175809)
Supplement: sj-pdf-4-hpq-10.1177_13591053231175809 – Supplemental material for How public trust and healthcare quality relate to blood donation behavior: Cross-cultural evidence [file sj-pdf-4-hpq-10.1177_13591053231175809.pdf]

README: Data sharing and replication instructions

This note accompanies the manuscript entitled *How Public Trust and Healthcare Quality Relate to Blood Donation Behavior: Cross-Cultural Evidence* by [details removed for peer review].

Overview of replication files:

- **P2_data_sharing_explanatory_note.docx**: DOCX document explaining how to conduct replication of all main and supplementary analyses reported in the main text and appendix of the manuscript.
- **P2_analysis_syntax.R** (identical to **P2_analysis_log.R**) is the R syntax script which can be executed by the statistical software R to reproduce all main and supplementary analyses reported in the main text and appendix of the manuscript. Note that R does not produce external log files; Output (including all figures and all statistical results) are produced by running P2_analysis_syntax.R within R.
- **P2_data_isocodes_and_explanation.csv** is a CSV file with the country codes used in the analysis. Note that all other datasets used for analyses are secondary data which need to be downloaded from the publicly accessible websites of their respective owners. Detailed instructions for downloading the data are provided in P2_data_sharing_explanatory_note.docx.

Instructions on accessing secondary datasets (all datasets are free and publicly accessible)

1. **Eurobarometer 82.2**

   Instructions: Download file ZA5931_v3-0-0.dta from https://doi.org/10.4232/1.12999 (free account with GESIS required)

2. **Global Burden of Diseases Collaborative: Healthcare Access and Quality Index**

   Instructions: Download folder IHME_GBD_2015_HAQ_INDEX_1990_2015 from http://ghdx.healthdata.org/record/ihme-data/gbd-2015-healthcare-access-and-quality-index-1990-2015 (click on 'files'; free)

3. **World Health Organization Global Health Expenditure database**

   Instructions: Download file total-healthcare-expenditure-as-share-of-national-gdp-by-country.csv: https://ourworldindata.org/grapher/total-healthcare-expenditure-as-share-of-national-gdp-by-country (select 'Download' and then 'full data (CSV)'; free)

4. **European Values Survey: EVS (2021)**

   Instructions: Download zip folder ZA7503_v2-0-0.dta.zip from https://doi.org/10.4232/1.13736 (free account with GESIS required)
