# Supplementary material for: How public trust and healthcare quality relate to blood donation behavior: Cross-cultural evidence
Source: J Health Psychol. 2023 Jun 5;29(1):3–14. doi: 10.1177/13591053231175809 (PMC10757392; doi:10.1177/13591053231175809)
Supplement: sj-pdf-5-hpq-10.1177_13591053231175809 – Supplemental material for How public trust and healthcare quality relate to blood donation behavior: Cross-cultural evidence [file sj-pdf-5-hpq-10.1177_13591053231175809.pdf]

```
R version 4.3.0 (2023-04-21) -- "Already Tomorrow"
Copyright (C) 2023 The R Foundation for Statistical Computing
Platform: x86_64-apple-darwin20 (64-bit)

R is free software and comes with ABSOLUTELY NO WARRANTY.
You are welcome to redistribute it under certain conditions.
Type 'license()' or 'licence()' for distribution details.

  Natural language support but running in an English locale

R is a collaborative project with many contributors.
Type 'contributors()' for more information and
'citation()' on how to cite R or R packages in publications.

Type 'demo()' for some demos, 'help()' for on-line help, or
'help.start()' for an HTML browser interface to help.
Type 'q()' to quit R.

> # Install the 'remotes' package if not already installed
> if (!requireNamespace("remotes", quietly = TRUE)) {
+   install.packages("remotes")
+ }
> # Load the 'remotes' package
> library(remotes)
> # Install an older version of rnaturalearth
> install_version("rnaturalearth", version = "0.1.0")
Downloading package from url:
https://cran.rstudio.com//src/contrib/Archive/rnaturalearth/rnaturalearth_0.1.0.tar.gz
These packages have more recent versions available.
It is recommended to update all of them.
Which would you like to update?

1: All
2: CRAN packages only
3: None
4: wk    (0.7.2  -> 0.7.3 ) [CRAN]
5: units (0.8-1  -> 0.8-2 ) [CRAN]
6: s2    (1.1.2  -> 1.1.4 ) [CRAN]
7: sf    (1.0-12 -> 1.0-13) [CRAN]
8: sp    (1.6-0  -> 1.6-1 ) [CRAN]

Enter one or more numbers, or an empty line to skip updates:
* installing *source* package ‘rnaturalearth’ ...
** package ‘rnaturalearth’ successfully unpacked and MD5 sums checked
** using staged installation
** R
** data
*** moving datasets to lazyload DB
** inst
** byte-compile and prepare package for lazy loading
** help
*** installing help indices
** building package indices
** installing vignettes
** testing if installed package can be loaded from temporary location
** testing if installed package can be loaded from final location
** testing if installed package keeps a record of temporary installation path
* DONE (rnaturalearth)
> ##### Load packages
> library("tidyverse")
── Attaching core tidyverse packages
──────────────────────────────────────────────────────────────────
 tidyverse 2.0.0 ──
✔ dplyr     1.1.2     ✔ readr     2.1.4
✔ forcats   1.0.0     ✔ stringr   1.5.0
✔ ggplot2   3.4.2     ✔ tibble    3.2.1
✔ lubridate 1.9.2     ✔ tidyr     1.3.0
✔ purrr     1.0.1
── Conflicts
──────────────────────────────────────────────────────────────────
 tidyverse_conflicts() ──
✖ dplyr::filter() masks stats::filter()
✖ dplyr::lag()    masks stats::lag()
ℹ Use the conflicted package to force all conflicts to become errors
> library("lme4")
Loading required package: Matrix
```

```
Attaching package: ‘Matrix’

The following objects are masked from ‘package:tidyr’:

    expand, pack, unpack

> library("haven")
> library("sjlabelled")

Attaching package: ‘sjlabelled’

The following objects are masked from ‘package:haven’:

    as_factor, read_sas, read_spss, read_stata, write_sas, zap_labels

The following object is masked from ‘package:forcats’:

    as_factor

The following object is masked from ‘package:dplyr’:

    as_label

The following object is masked from ‘package:ggplot2’:

    as_label

> library("data.table")
data.table 1.14.8 using 1 threads (see ?getDTthreads).  Latest news: r-datatable.com
**********
This installation of data.table has not detected OpenMP support. It should still work but in single-threaded mode.
This is a Mac. Please read https://mac.r-project.org/openmp/. Please engage with Apple and ask them for support.
Check r-datatable.com for updates, and our Mac instructions here:
https://github.com/Rdatatable/data.table/wiki/Installation. After several years of many reports of installation
problems on Mac, it's time to gingerly point out that there have been no similar problems on Windows or Linux.
**********

Attaching package: ‘data.table’

The following objects are masked from ‘package:lubridate’:

    hour, isoweek, mday, minute, month, quarter, second, wday, week, yday, year

The following objects are masked from ‘package:dplyr’:

    between, first, last

The following object is masked from ‘package:purrr’:

    transpose

> library("scales")

Attaching package: ‘scales’

The following object is masked from ‘package:purrr’:

    discard

The following object is masked from ‘package:readr’:

    col_factor

> library("RColorBrewer")
> library("psych")

Attaching package: ‘psych’

The following objects are masked from ‘package:scales’:

    alpha, rescale

The following objects are masked from ‘package:ggplot2’:

    %+%, alpha
```

```
> library("ggrepel")
> library("sf")
Linking to GEOS 3.11.0, GDAL 3.5.3, PROJ 9.1.0; sf_use_s2() is TRUE
> library("rnaturalearth")
> library("ggpubr")
> library("corrplot")
corrplot 0.92 loaded
> library("Hmisc")

Attaching package: ‘Hmisc’

The following object is masked from ‘package:psych’:

    describe

The following objects are masked from ‘package:dplyr’:

    src, summarize

The following objects are masked from ‘package:base’:

    format.pval, units

> library("ggrepel")
> ##### Set seed for reproducibility
> set.seed(101)
> # Eurobarometer (2014 wave)
> eurobarometer_data_raw = read_dta("../data/Eurobarometer/ZA5931_v3-0-0.dta")
> # European Values Survey: Confidence in healthcare system
> evs_raw = read_dta("../data/EVS/ZA7503_v2-0-0.dta/ZA7503_v2-0-0.dta")
> # HAQ
> haq_raw =
read.csv("../data/HAQ/IHME_GBD_2015_HAQ_INDEX_1990_2015/IHME_GBD_2015_HAQ_INDEX_1990_2015_HAQ_INDEX_AND_VALUES_Y201
> # Healthcare expenditures (OWID from World Bank - World Development Indicators)
> health_exp_raw = read.csv("../data/OWID health expenditures/total-healthcare-expenditure-as-share-of-national-
gdp-by-country.csv")
> # Country codes
> iso_code_alpha2 = read.csv("../data/iso_code_alpha2.csv")
> # UN_geoscheme_classification
> UN_geoscheme_classification = read.csv("../data/UN_geoscheme_classification.csv")
> ################################################################
>
> ####### 1) Preprocessing
>
> ### A) European Values Survey: Confidence in healthcare system
>
> evs = dplyr::select(evs_raw, S007, S009, S020, E069_16)%>%
+   rename(resp_num = S007, iso_code_alpha2 = S009, year = S020, confidence_healthcare = E069_16)%>%
+   filter(confidence_healthcare > 0 & confidence_healthcare < 5 & year < 2014)%>%
+   mutate(confidence_healthcare = ifelse(confidence_healthcare == 1, 4,
+                                        ifelse(confidence_healthcare == 2, 3,
+                                               ifelse(confidence_healthcare == 3, 2,
+                                                      ifelse(confidence_healthcare == 4, 1, NA)))))%>% #
reverse-code
+   mutate(year = as.numeric(year),
+          confidence_healthcare = as.numeric(confidence_healthcare))
>
> # Filter by Eurobarometer countries
> eurobarometer_countries = unique(eurobarometer_data_raw$isocntry)
> eurobarometer_countries = ifelse(eurobarometer_countries == "DE-W", "DE",
+                                  ifelse(eurobarometer_countries == "DE-E", "DE", eurobarometer_countries))
>
> iso_code_alpha2 = iso_code_alpha2%>%
+   mutate(country_name = ifelse(country_name == "United Kingdom of Great Britain and Northern Ireland", "United
Kingdom",
+                                ifelse(country_name == "Czechia", "Czech Republic", country_name)))
>
> eurobarometer_countries_names = filter(iso_code_alpha2, iso_code_alpha2 %in% eurobarometer_countries |
iso_code_alpha2 == "GB")
>
> evs_filtered = filter(evs, iso_code_alpha2 %in% eurobarometer_countries)
>
> evs_agg = evs_filtered%>%
+   group_by(iso_code_alpha2, year)%>%
+   summarise(mean_confidence_healthcare = mean(confidence_healthcare))%>%
+   ungroup()
`summarise()` has grouped output by 'iso_code_alpha2'. You can override using the `.groups` argument.
```

```
>
>
> # Get UK estimate by weighting
> # Weight UK EVS estimate by population (GB and NIR)
> # Population UK (= GB + NIR): 58.68 mio. (1999); 61.81 mio. (2008)
> # Population NIR: 1.679 mio. (1999); 1.77 mio. (2008)
> # Population GB (calculated with UK - NIR): 57.001 mio. (1999); 60.04 mio. (2008)
> mean_confidence_healthcare_UK_1999 = weighted.mean(c(2.634221, 2.832653), c(57.001, 1.679))
> mean_confidence_healthcare_UK_2008 = weighted.mean(c(3.046572, 2.692929), c(60.04, 1.77))
>
> evs_agg = evs_agg%>%
+   add_row(iso_code_alpha2 = "GB", year = 1999, mean_confidence_healthcare =
mean_confidence_healthcare_UK_1999)%>%
+   add_row(iso_code_alpha2 = "GB", year = 2008, mean_confidence_healthcare =
mean_confidence_healthcare_UK_2008)%>%
+   add_row(iso_code_alpha2 = "CY", year = 1999, mean_confidence_healthcare = 2.662091)%>% # add dummy Cyprus data
for year 1999
+   filter(iso_code_alpha2 != "GB-NIR" & iso_code_alpha2 != "GB-GBN")%>%
+   mutate(year = ifelse(year == 2009, 2008,
+                        ifelse(year == 2000, 1999, year)))%>%
+   rename(year_EVS = year)
>
>
> ### B) HAQ
>
> haq = filter(haq_raw, indicator_name == "Healthcare Access and Quality", location_name %in%
eurobarometer_countries_names$country_name)%>%
+   select(location_name, year_id, val)%>%
+   rename(country_name = location_name, year_haq = year_id, haq_index = val)
>
>
> ### C) Healthcare expenditures
>
> health_exp = mutate(health_exp_raw, Entity = ifelse(Entity == "Czechia", "Czech Republic", Entity))%>%
+   filter(Entity %in% eurobarometer_countries_names$country_name)%>%
+   select(Entity, Year, Health.expenditure..total....of.GDP.)%>%
+   rename(country_name = Entity, year_health_exp = Year, health_exp_per_GDP =
Health.expenditure..total....of.GDP.)
>
>
> ### D) Eurobarometer
>
> # Select relevant variables
> eurobarometer_data = dplyr::select(eurobarometer_data_raw, uniqid, nuts, nutslvl, isocntry, qe1_1, d15a, d40b,
d25, d11, d8, d10, d7)
>
> # Remove labels
> eurobarometer_data = remove_all_labels(eurobarometer_data)
> eurobarometer_data = zap_formats(eurobarometer_data)
>
> # Rename columns
> eurobarometer_data = rename(eurobarometer_data,
+                             donate_blood = qe1_1,
+                             employment_status = d15a,
+                             parental_status = d40b,
+                             type_of_community = d25,
+                             age = d11,
+                             education = d8,
+                             gender = d10,
+                             partner_status = d7)
>
> # Recode country code
> eurobarometer_data$iso_code_alpha2 = ifelse(eurobarometer_data$isocntry == "DE-W", "DE",
+                                             ifelse(eurobarometer_data$isocntry == "DE-E", "DE",
+                                                    ifelse(eurobarometer_data$isocntry == "GB-GBN", "GB",
+                                                           ifelse(eurobarometer_data$isocntry == "GB-NIR", "GB",
eurobarometer_data$isocntry))))
>
>
> # Variable transformations & converting variables to right format
> data = eurobarometer_data %>%
+   mutate(donate_blood = ifelse(donate_blood == 1 | donate_blood == 2, 1, 0),
+          employment_status = ifelse(employment_status == 1 | employment_status == 2 | employment_status == 3 |
employment_status == 4, 0, 1),
+          education = ifelse(education == 97, 0, ifelse(education == 98, age, education)),
+          parental_status = ifelse(parental_status > 0, 1, 0),
+          partner_status = ifelse(partner_status > 0 & partner_status < 9, 1, 0),
```

```
+          type_of_community = ifelse(type_of_community == 1, 2, ifelse(type_of_community == 2, 1,
ifelse(type_of_community == 3, 0, NA))),
+          gender = gender - 1)%>%
+   mutate(donate_blood = as.factor(donate_blood),
+          type_of_community = as.factor(type_of_community),
+          parental_status = as.factor(parental_status),
+          gender = as.factor(gender),
+          iso_code_alpha2 = as.factor(iso_code_alpha2),
+          partner_status = as.factor(partner_status),
+          employment_status = as.factor(employment_status))%>%
+   mutate(index_time_r = ifelse(age < 66, round(2014 - (age - 18)/2, 0),
+                                ifelse(age > 65, round(2014 - (65 - 18)/2 - (age - 65), 0), NA)),
+          year_EVS = ifelse(index_time_r > 2003, 2008, 1999),
+          year_haq = ifelse(index_time_r < 1993, 1990,
+                            ifelse(index_time_r < 1998, 1995,
+                                   ifelse(index_time_r < 2003, 2000,
+                                          ifelse(index_time_r < 2008, 2005,
+                                                 ifelse(index_time_r < 2013, 2010,
+                                                        ifelse(index_time_r < 2018, 2015, NA)))))),
+          year_health_exp = ifelse(index_time_r < 1995, 1995, index_time_r))
>
>
> ### E) Merge country-level variables with individual-level data (Eurobarometer)
>
> # Merge country-level variables with individual-level data (Eurobarometer)
> data_full = left_join(data, eurobarometer_countries_names, by = "iso_code_alpha2")
> data_full = left_join(data_full, evs_agg, by=c("year_EVS", "iso_code_alpha2"))
> data_full = left_join(data_full, haq, by=c("year_haq", "country_name"))
> data_full = left_join(data_full, health_exp, by=c("year_health_exp", "country_name"))
> data_full = left_join(data_full, UN_geoscheme_classification, by="country_name")
>
>
> ### F) Add country-level IVs aggregated over years
>
> # Calculate trust
> evs_agg_no_time = evs_filtered%>%
+   group_by(iso_code_alpha2)%>%
+   summarise(mean_confidence_healthcare_agg_no_time = mean(confidence_healthcare))
>
> mean_confidence_healthcare_UK = weighted.mean(c(2.886994, 2.785763), c(57.001, 1.679))
> evs_agg_no_time = evs_agg_no_time%>% add_row(iso_code_alpha2 = "GB", mean_confidence_healthcare_agg_no_time =
mean_confidence_healthcare_UK)
>
> # Calculate HAQ
> haq_agg_no_time = haq%>%
+   group_by(country_name)%>%
+   summarise(haq_agg_no_time = mean(haq_index))
>
> # Calculate expenditures
> health_exp_agg_no_time = health_exp%>%
+   group_by(country_name)%>%
+   summarise(health_exp_per_GDP_agg_no_time = mean(health_exp_per_GDP))
>
> # Merge with full data
> data_full = left_join(data_full, evs_agg_no_time, by="iso_code_alpha2")
> data_full = left_join(data_full, haq_agg_no_time, by="country_name")
> data_full = left_join(data_full, health_exp_agg_no_time, by="country_name")
>
> ### Convert variables to right format
> data_full = data_full%>%
+   rename(country = country_name)%>%
+   mutate(iso_code_alpha2 = as.factor(iso_code_alpha2),
+          country = as.factor(country),
+          UN_geoscheme_classification = as.factor(UN_geoscheme_classification))
>
>
> ### G) Exclude observations with NA in DV and respondents younger than 18
>
> ## Raw data: number of respondents
> nrow(data_full) # 27868
[1] 27868
>
> # Remove invalid data
> data_full = filter(data_full, !is.na(donate_blood))
> nrow(data_full) # 27082
[1] 27082
>
```

```
> # Remove respondents under 18
> data_full = filter(data_full, age > 17)
> nrow(data_full) # 26532
[1] 26532
>
>
> ################################################################
>
> ####### 2) Descriptives:
>
> ##### a) Summary statistics
>
> # Individual-level
> summary(data_full$donate_blood) # 0: 16337; 1: 10195
    0     1
16337 10195
> 10195 / 26532 # 38.4%
[1] 0.384253
>
> describe(data_full$donate_blood) # mean: 0.38
data_full$donate_blood
       n  missing distinct
   26532        0        2

Value          0     1
Frequency  16337 10195
Proportion 0.616 0.384
> describe(data_full$gender) # 0: 11653; 1: 14879
data_full$gender
       n  missing distinct
   26532        0        2

Value          0     1
Frequency  11653 14879
Proportion 0.439 0.561
> psych::describe(data_full$age) # mean 51.3; median 52; range 18 - 99
   vars     n  mean    sd median trimmed   mad min max range  skew kurtosis   se
X1    1 26532 51.29 17.81     52    51.3 20.76  18  99    81 -0.01    -0.96 0.11
> psych::describe(data_full$education) # mean 19.24; median 18; range 0 - 89; NA: 457
   vars     n  mean   sd median trimmed  mad min max range skew kurtosis   se
X1    1 26075 19.24 5.53     18   18.89 2.97   0  89    89 1.67    12.24 0.03
> describe(data_full$partner_status) # 0: 9260 ; 1: 17216; NA: 56
data_full$partner_status
       n  missing distinct
   26476       56        2

Value          0     1
Frequency   9260 17216
Proportion  0.35  0.65
> describe(data_full$employment_status) # 0: 13638; 1: 12894
data_full$employment_status
       n  missing distinct
   26532        0        2

Value          0     1
Frequency  13638 12894
Proportion 0.514 0.486
> describe(data_full$parental_status) # 0: 21507 ; 1: 5020; NA: 5
data_full$parental_status
       n  missing distinct
   26527        5        2

Value          0     1
Frequency  21507  5020
Proportion 0.811 0.189
> describe(data_full$type_of_community) # 0: 7228; 1: 11153; 2: 8137; NA: 14
data_full$type_of_community
       n  missing distinct
   26518       14        3

Value          0     1     2
Frequency   7228 11153  8137
Proportion 0.273 0.421 0.307
>
> colSums(!is.na(data_full))
                                uniqid                                   nuts
nutslvl                               isocntry
```

```
                               26532                                  26532
26532                                26532
                        donate_blood                       employment_status
parental_status                     type_of_community
                               26532                                  26532
26527                                26518
                                 age                               education
gender                          partner_status
                               26532                                  26075
26532                                26476
                     iso_code_alpha2                            index_time_r
year_EVS                             year_haq
                               26532                                  26532
26532                                26532
                     year_health_exp                                 country
mean_confidence_healthcare                          haq_index
                               26532                                  26532
26532                                26532
                  health_exp_per_GDP            UN_geoscheme_classification
mean_confidence_healthcare_agg_no_time                          haq_agg_no_time
                               26532                                  26532
26532                                26532
        health_exp_per_GDP_agg_no_time
                               26532
>
> # Country-level
> psych::describe(data_full$mean_confidence_healthcare)
   vars     n mean   sd median trimmed  mad  min  max range  skew kurtosis se
X1    1 26532 2.61 0.31   2.66    2.62 0.36 1.84 3.23  1.38 -0.13    -0.75  0
> psych::describe(data_full$haq_index)
   vars     n  mean   sd median trimmed  mad  min  max range  skew kurtosis   se
X1    1 26532 75.56 6.93   76.1   75.78 7.41 58.3 90.5  32.2 -0.27    -0.62 0.04
> psych::describe(data_full$health_exp_per_GDP)
   vars     n mean   sd median trimmed  mad  min   max range skew kurtosis   se
X1    1 26532 7.52 1.64   7.44    7.51 1.49 3.21 11.97  8.75 0.05    -0.16 0.01
> psych::describe(evs_agg)
                           vars  n    mean   sd  median trimmed   mad     min     max range  skew kurtosis   se
iso_code_alpha2*              1 56   14.50 8.15   14.50   14.50 10.38    1.00   28.00 27.00  0.00    -1.27 1.09
year_EVS                      2 56 2003.50 4.54 2003.50 2003.50  6.67 1999.00 2008.00  9.00  0.00    -2.04 0.61
mean_confidence_healthcare    3 56    2.63 0.33    2.66    2.63  0.36    1.84    3.23  1.38 -0.14    -0.78 0.04
> psych::describe(haq)
             vars   n    mean   sd median trimmed   mad    min    max range  skew kurtosis   se
country_name*    1 168   14.50 8.10   14.5   14.50 10.38    1.0   28.0  27.0  0.00    -1.22 0.63
year_haq         2 168 2002.50 8.56 2002.5 2002.50 11.12 1990.0 2015.0  25.0  0.00    -1.29 0.66
haq_index        3 168   77.78 7.38   78.8   78.17  8.01   58.3   90.5  32.2 -0.43    -0.61 0.57
> psych::describe(health_exp)
                  vars   n    mean   sd median trimmed   mad     min     max range skew kurtosis   se
country_name*         1 560   14.50 8.08   14.5   14.50 10.38    1.00   28.00 27.00 0.00    -1.21 0.34
year_health_exp       2 560 2004.50 5.77 2004.5 2004.50  7.41 1995.00 2014.00 19.00 0.00    -1.21 0.24
health_exp_per_GDP    3 560    7.91 1.72    7.8    7.88  2.02    3.21   11.97  8.75 0.13    -0.61 0.07
>
>
> # Country-level indicator values and availability
> country_level_indicator_descriptives = data_full%>%
+   group_by(country)%>%
+   dplyr::summarise(country_mean_confidence_healthcare = round(mean(mean_confidence_healthcare), 2),
+                    sd_confidence_healthcare = round(sd(mean_confidence_healthcare), 2),
+                    country_haq_index = round(mean(haq_index), 2),
+                    sd_haq_index = round(sd(haq_index), 2),
+                    country_health_exp_per_GDP = round(mean(health_exp_per_GDP), 2),
+                    sd_health_exp_per_GDP = round(sd(health_exp_per_GDP), 2),
+                    donate_blood = round(mean(as.numeric(as.character(donate_blood))), 2),
+                    num_resp = n(),
+                    UN_geoscheme_classification = unique(UN_geoscheme_classification))
>
> # write.csv(country_level_indicator_descriptives, "country_level_indicator_descriptives.csv")
>
>
> ################################################################
>
> ##### b) Map plots
>
> # Map plotting based on tutorial here: https://bhaskarvk.github.io/user2017.geodataviz/notebooks/02-Static-
Maps.nb.html#
>
> ### Initiation of map data
>
```

```
> # Get map data
> world <- st_as_sf(rnaturalearth::countries110)
> europe <- dplyr::filter(world, region_un=="Europe" & name!='Russia')
>
> # Filter polygons that are part of continental Europe with the help of a bounding box
> europe.bbox <- st_polygon(list(
+   matrix(c(-25,29,45,29,45,75,-25,75,-25,29),byrow = T,ncol = 2)))
>
> europe.clipped <- suppressWarnings(st_intersection(europe, st_sfc(europe.bbox, crs=st_crs(europe))))
>
>
> ## EVS
> data_maps_evs = evs_agg%>%
+   rename(iso_a2 = iso_code_alpha2)%>%
+   mutate(iso_a2 = as.character(iso_a2),
+          year_EVS = as.character(year_EVS))
>
> mean_EVS = data_maps_evs%>% group_by(iso_a2)%>%dplyr::summarize(mean_confidence_healthcare =
mean(mean_confidence_healthcare))%>%mutate(year_EVS = "mean 1999-2008")
>
> data_maps_evs = union(data_maps_evs, mean_EVS)
>
> # Add to map data
> map_data_evs = left_join(europe.clipped, data_maps_evs, by="iso_a2")%>%
+   filter(!is.na(year_EVS))
>
> # Two maps for mean and time_series
> map_data_evs_mean = filter(map_data_evs, year_EVS == "mean 1999-2008")
> map_data_evs_timeseries = filter(map_data_evs, year_EVS != "mean 1999-2008")
>
> map_evs_mean = ggplot(data=subset(map_data_evs_mean, sovereignt != "Belarus" & sovereignt != "Ukraine" &
sovereignt != "Moldova"), aes(fill=mean_confidence_healthcare)) +
+   geom_sf(alpha=0.8,col='white') +
+   coord_sf(crs="+proj=aea +lat_1=36.333333333333336 +lat_2=65.66666666666667 +lon_0=14") +
+   viridis::scale_fill_viridis(name='', direction = 1, na.value = "grey92") +
+   labs(x=NULL, y=NULL, title="Trust in the healthcare system")+
+   theme(legend.position="bottom", legend.direction = "horizontal", legend.key.width = unit(1.3,"cm"), plot.title
= element_text(hjust = 0.5))
>
> ggplot(data=subset(map_data_evs_timeseries, sovereignt != "Belarus" & sovereignt != "Ukraine" & sovereignt !=
"Moldova"), aes(fill=mean_confidence_healthcare)) +
+   geom_sf(alpha=0.8,col='white') +
+   coord_sf(crs="+proj=aea +lat_1=36.333333333333336 +lat_2=65.66666666666667 +lon_0=14") +
+   viridis::scale_fill_viridis(name='', direction = 1, na.value = "grey92") +
+   labs(x=NULL, y=NULL, title="Trust in the healthcare system")+
+   facet_grid(~ year_EVS)+
+   theme(legend.position="bottom", legend.direction = "horizontal", legend.key.width = unit(1.3,"cm"), plot.title
= element_text(hjust = 0.5))
>
> #ggsave("plots/maps/trust_timeseries.png", width = 11.9, height = 7.5, units = "in")
>
>
> ## HAQ
> haq = left_join(haq, eurobarometer_countries_names, by="country_name")%>%
+   select(-country_name)
>
> data_maps_haq = haq%>%
+   rename(iso_a2 = iso_code_alpha2)%>%
+   mutate(iso_a2 = as.character(iso_a2),
+          year_haq = as.character(year_haq))
>
> mean_haq = data_maps_haq%>% group_by(iso_a2)%>%dplyr::summarize(haq_index = mean(haq_index))%>%mutate(year_haq =
"mean 1990-2015")
>
> data_maps_haq = union(data_maps_haq, mean_haq)
>
> # Add to map data
> map_data_haq = left_join(europe.clipped, data_maps_haq, by="iso_a2")%>%
+   filter(!is.na(year_haq))
>
> # Two maps for mean and time_series
> map_data_haq_mean = filter(map_data_haq, year_haq == "mean 1990-2015")
> map_data_haq_timeseries = filter(map_data_haq, year_haq != "mean 1990-2015")
>
> map_haq_mean = ggplot(data=subset(map_data_haq_mean, sovereignt != "Belarus" & sovereignt != "Ukraine" &
sovereignt != "Moldova"), aes(fill=haq_index)) +
+   geom_sf(alpha=0.8,col='white') +
```

```
+   coord_sf(crs="+proj=aea +lat_1=36.333333333333336 +lat_2=65.66666666666667 +lon_0=14") +
+   viridis::scale_fill_viridis(name='', direction = 1, na.value = "grey92") +
+   labs(x=NULL, y=NULL, title="HAQ index")+
+   theme(legend.position="bottom", legend.direction = "horizontal", legend.key.width = unit(1.3,"cm"), plot.title
= element_text(hjust = 0.5))
>
> ggplot(data=subset(map_data_haq_timeseries, sovereignt != "Belarus" & sovereignt != "Ukraine" & sovereignt !=
"Moldova"), aes(fill=haq_index)) +
+   geom_sf(alpha=0.8,col='white') +
+   coord_sf(crs="+proj=aea +lat_1=36.333333333333336 +lat_2=65.66666666666667 +lon_0=14") +
+   viridis::scale_fill_viridis(name='', direction = 1, na.value = "grey92") +
+   labs(x=NULL, y=NULL, title="HAQ index")+
+   facet_wrap(~ year_haq, ncol=3)+
+   theme(legend.position="bottom", legend.direction = "horizontal", legend.key.width = unit(1.3,"cm"), plot.title
= element_text(hjust = 0.5))
>
> #ggsave("plots/maps/haq_timeseries.png", width = 8.75, height = 8, units = "in")
>
>
> ## health_exp
> health_exp = left_join(health_exp, eurobarometer_countries_names, by="country_name")%>%
+   select(-country_name)
>
> data_maps_health_exp = health_exp%>%
+   rename(iso_a2 = iso_code_alpha2)%>%
+   mutate(iso_a2 = as.character(iso_a2),
+          year_health_exp = as.character(year_health_exp))
>
> mean_health_exp = data_maps_health_exp%>% group_by(iso_a2)%>%dplyr::summarize(health_exp_per_GDP =
mean(health_exp_per_GDP))%>%mutate(year_health_exp = "mean 1995-2014")
>
> data_maps_health_exp = union(data_maps_health_exp, mean_health_exp)
>
> # Add to map data
> map_data_health_exp = left_join(europe.clipped, data_maps_health_exp, by="iso_a2")%>%
+   filter(!is.na(year_health_exp))
>
> # Two maps for mean and time_series
> map_data_health_exp_mean = filter(map_data_health_exp, year_health_exp == "mean 1995-2014")
> map_data_health_exp_timeseries = filter(map_data_health_exp, year_health_exp != "mean 1995-2014")
>
> map_health_exp_mean = ggplot(data=subset(map_data_health_exp_mean, sovereignt != "Belarus" & sovereignt !=
"Ukraine" & sovereignt != "Moldova"), aes(fill=health_exp_per_GDP)) +
+   geom_sf(alpha=0.8,col='white') +
+   coord_sf(crs="+proj=aea +lat_1=36.333333333333336 +lat_2=65.66666666666667 +lon_0=14") +
+   viridis::scale_fill_viridis(name='', direction = 1, na.value = "grey92") +
+   labs(x=NULL, y=NULL, title="Health expenditures per GDP")+
+   theme(legend.position="bottom", legend.direction = "horizontal", legend.key.width = unit(1.3,"cm"), plot.title
= element_text(hjust = 0.5))
>
>
> ggplot(data=subset(map_data_health_exp_timeseries, sovereignt != "Belarus" & sovereignt != "Ukraine" &
sovereignt != "Moldova"), aes(fill=health_exp_per_GDP)) +
+   geom_sf(alpha=0.8,col='white') +
+   coord_sf(crs="+proj=aea +lat_1=36.333333333333336 +lat_2=65.66666666666667 +lon_0=14") +
+   viridis::scale_fill_viridis(name='', direction = 1, na.value = "grey92") +
+   labs(x=NULL, y=NULL, title="Health expenditures per GDP")+
+   facet_wrap(~ year_health_exp, ncol=5)+
+   theme(legend.position="bottom", legend.direction = "horizontal", legend.key.width = unit(1.3,"cm"), plot.title
= element_text(hjust = 0.5))
>
> #ggsave("plots/maps/health_exp_timeseries.png", width = 12.1, height = 10.8, units = "in")
>
>
> ## Blood donation
>
> # Calculate country-level means
> blood_donation_agg = data_full%>%
+   group_by(iso_code_alpha2)%>%
+   dplyr::summarize(donate_blood_agg = mean(as.numeric(as.character(donate_blood)), na.rm=T))%>%
+   rename(iso_a2 = iso_code_alpha2)%>%
+   mutate(iso_a2 = as.character(iso_a2))
>
> map_data_blood_donation = left_join(europe.clipped, blood_donation_agg, by="iso_a2")
>
> ggplot(map_data_blood_donation, aes(fill=donate_blood_agg)) +
+   geom_sf(alpha=0.8,col='white') +
```

```
+   coord_sf(crs="+proj=aea +lat_1=36.333333333333336 +lat_2=65.66666666666667 +lon_0=14") +
+   viridis::scale_fill_viridis(name='Country-level mean\nblood donation', direction = -1, labels=scales::percent,
option="plasma") +
+   labs(x=NULL, y=NULL, title=NULL)
>
> #ggsave("plots/maps/blood_donation.png", width = 7, height = 5.7, units = "in")
>
>
> ################################################################
>
> ##### c) Density plots of indicators over time
>
> # A) Trust
> evs_agg$year_EVS = as.factor(evs_agg$year_EVS)
>
> density1 = ggplot(data=evs_agg, aes(mean_confidence_healthcare, color = year_EVS, fill = year_EVS)) +
+   geom_density(alpha = 0.4)+
+   guides(fill = "none")+
+   labs(x = "Trust in the healthcare system", color="Year")
>
> # B) HAQ
> haq$year_haq = as.factor(haq$year_haq)
>
> density2 = ggplot(data=haq, aes(haq_index, color = year_haq, fill = year_haq)) +
+   geom_density(alpha = 0.2)+
+   guides(fill = "none")+
+   labs(x = "HAQ index", color="Year")
>
> # C) Health expenditures
> health_exp$year_health_exp = as.factor(health_exp$year_health_exp)
>
> density3 = ggplot(data=health_exp, aes(health_exp_per_GDP, color = year_health_exp, fill = year_health_exp)) +
+   geom_density(alpha = 0.08)+
+   guides(fill = "none")+
+   labs(x = "Health expenditures per GDP", color="Year")
>
> # D) Histogram of index_time_r
> ggplot(data=data_full, aes(index_time_r)) +
+   geom_histogram(bins=20) +
+   labs(x = "Assumed time point of donation", y="Number of respondents")
>
> # ggsave("plots/density/index_time_r.png", width = 8.4, height = 7, units = "in")
>
> # E) Make combined plot for paper
> ggarrange(density1, density2, density3,
+           labels = c("A", "B", "C"),
+           ncol = 3, nrow = 1)
>
> #ggsave("plots/density/combined_indicators_density.png", width = 15.3, height = 6, units = "in")
>
>
> ################################################################
>
> ##### d) Time series plots of indicators over time
>
> # A) Trust
> evs_agg = left_join(evs_agg, eurobarometer_countries_names, by="iso_code_alpha2")
>
> ggplot(evs_agg, aes(year_EVS, mean_confidence_healthcare, color=iso_code_alpha2, group = iso_code_alpha2,
label=country_name)) +
+   geom_line(aes()) +
+   geom_text_repel() +
+   labs(y = "Trust in the healthcare system", x = "Survey wave") +
+   guides(color = "none")+
+   ylim(1, 4)
Warning message:
ggrepel: 14 unlabeled data points (too many overlaps). Consider increasing max.overlaps
>
> #ggsave("plots/line/trust_timeseries_fullscale.png", width = 7, height = 12, units = "in")
>
> # B) HAQ
> haq = left_join(haq, eurobarometer_countries_names, by="iso_code_alpha2")
> haq$country_name_alt = ifelse(haq$year_haq == 1990 | haq$year_haq == 2015, haq$country_name, "")
>
> ggplot(haq, aes(year_haq, haq_index, color=iso_code_alpha2, group = iso_code_alpha2, label=country_name_alt)) +
+   geom_line(aes()) +
+   geom_text_repel(max.overlaps =24) +
```

```
+   labs(y = "HAQ index", x = "Survey wave") +
+   guides(color = "none")+
+   ylim(0, 100)
Warning message:
ggrepel: 8 unlabeled data points (too many overlaps). Consider increasing max.overlaps
>
> #ggsave("plots/line/haq_timeseries_fullscale.png", width = 11.4, height = 15, units = "in")
>
> # C) Health expenditures
> health_exp = left_join(health_exp, eurobarometer_countries_names, by="iso_code_alpha2")
> health_exp$country_name_alt = ifelse(health_exp$year_health_exp == 1995 | health_exp$year_health_exp == 2014,
health_exp$country_name, "")
>
> ggplot(health_exp, aes(year_health_exp, health_exp_per_GDP, color=iso_code_alpha2, group = iso_code_alpha2,
label=country_name_alt)) +
+   geom_line(aes()) +
+   geom_text_repel() +
+   labs(y = "Health expenditures per GDP", x = "Survey wave") +
+   guides(color = "none")
Warning message:
ggrepel: 6 unlabeled data points (too many overlaps). Consider increasing max.overlaps
>
> #ggsave("plots/line/health_exp_timeseries.png", width = 11.4, height = 17, units = "in")
>
> ggplot(health_exp, aes(year_health_exp, health_exp_per_GDP, color=iso_code_alpha2, group = iso_code_alpha2,
label=country_name_alt)) +
+   geom_smooth(method = "glm", alpha=0.2) +
+   geom_text_repel() +
+   labs(y = "Health expenditures per GDP", x = "Survey wave") +
+   guides(color = "none")
`geom_smooth()` using formula = 'y ~ x'
Warning messages:
1: The following aesthetics were dropped during statistical transformation: label
ℹ This can happen when ggplot fails to infer the correct grouping structure in the data.
ℹ Did you forget to specify a `group` aesthetic or to convert a numerical variable into a factor?
2: ggrepel: 6 unlabeled data points (too many overlaps). Consider increasing max.overlaps
>
> #ggsave("plots/line/health_exp_timeseries_smooth.png", width = 9, height = 14, units = "in")
>
>
> ################################################################
>
> ##### e) Scatter plot of observed country-level mean blood donation rates
>
> # To distinguish different European regions, include as aesthetic ", color=UN_geoscheme_classification"
>
> # Scatter plot: mean_confidence_healthcare
> observed_country_trust = ggplot(country_level_indicator_descriptives, aes(country_mean_confidence_healthcare,
donate_blood)) +
+   stat_smooth(method="glm", formula=y~x, alpha=0.2, size=2) +
+   geom_point() +
+   geom_text(aes(label=country), color = "black", hjust=-0.08, vjust=0) +
+   labs(y = "Observed country-level mean levels of blood donation", x = "Trust in the healthcare system") +
+   theme(legend.position="bottom", plot.title = element_text(hjust = 0.5))
Warning messages:
1: ggrepel: 24 unlabeled data points (too many overlaps). Consider increasing max.overlaps
2: ggrepel: 24 unlabeled data points (too many overlaps). Consider increasing max.overlaps
3: Using `size` aesthetic for lines was deprecated in ggplot2 3.4.0.
ℹ Please use `linewidth` instead.
This warning is displayed once every 8 hours.
Call `lifecycle::last_lifecycle_warnings()` to see where this warning was generated.
>
> # Scatter plot: HAQ
> observed_country_haq = ggplot(country_level_indicator_descriptives, aes(country_haq_index, donate_blood)) +
+   stat_smooth(method="glm", formula=y~x, alpha=0.2, size=2) +
+   geom_point() +
+   geom_text(aes(label=country), color = "black", hjust=-0.08, vjust=0) +
+   labs(y = "Observed country-level mean levels of blood donation", x = "HAQ index") +
+   theme(legend.position="bottom", plot.title = element_text(hjust = 0.5))
>
> # Scatter plot: health_exp_per_GDP
> observed_country_exp = ggplot(country_level_indicator_descriptives, aes(country_health_exp_per_GDP,
donate_blood)) +
+   stat_smooth(method="glm", formula=y~x, alpha=0.2, size=2) +
+   geom_point() +
+   geom_text(aes(label=country), color = "black", hjust=-0.08, vjust=0) +
+   labs(y = "Observed country-level mean levels of blood donation", x = "Health expenditures per GDP") +
```

```
+   theme(legend.position="bottom", plot.title = element_text(hjust = 0.5))
>
> # make combined descriptves plot for paper SI
> ggarrange(observed_country_trust, observed_country_haq, observed_country_exp,
+           labels = c("A", "B", "C"),
+           ncol = 3, nrow = 1)
>
> #ggsave("plots/scatter/combined_descr.png", width = 15.6, height = 7.49, units = "in")
>
> ################################################################
>
> ##### f) Correlations
>
> ### Plot correlations
> data_variables_of_interest = select(data_full, donate_blood, age, gender, education, partner_status,
employment_status, parental_status, type_of_community, mean_confidence_healthcare, haq_index,
health_exp_per_GDP)%>%
+   # delete categorical variables with more than two levels
+   select(-type_of_community)%>%
+   # convert all (binary) factor variables to numeric (Note that we calculate a Pearson correlation if a
categorical variable has a 0/1-coding (--> point-biserial correlation coefficient))
+   mutate_if(is.factor, as.character)%>%
+   mutate_if(is.character, as.numeric)%>%
+   # Rename as in model
+   rename(`Blood donation` = donate_blood,
+          `Gender (female = 1)` = gender,
+          `Age (years)` = age,
+          `Education (years)` = education,
+          `Partner status` = partner_status,
+          `Employment status` = employment_status,
+          `Parental status` = parental_status,
+          `Country-level trust in the healthcare system` = mean_confidence_healthcare,
+          `Country-level HAQ index` = haq_index,
+          `Country-level healthcare expenditures per GDP` = health_exp_per_GDP)
>
> correlations = cor(data_variables_of_interest, use = "complete.obs", method = "pearson")
> corrplot(correlations, type="lower", col=brewer.pal(n=8, name="RdYlBu"))
>
> ### Indicator correlations
>
> # a) Country-level
> cor.test(country_level_indicator_descriptives$country_mean_confidence_healthcare,
country_level_indicator_descriptives$country_haq_index, method = "pearson")

    Pearson's product-moment correlation

data:  country_level_indicator_descriptives$country_mean_confidence_healthcare and
country_level_indicator_descriptives$country_haq_index
t = 2.7564, df = 26, p-value = 0.01054
alternative hypothesis: true correlation is not equal to 0
95 percent confidence interval:
 0.1245581 0.7207451
sample estimates:
      cor
0.4755369

> # 0.48, p < 0.05
> cor.test(country_level_indicator_descriptives$country_mean_confidence_healthcare,
country_level_indicator_descriptives$country_health_exp_per_GDP, method = "pearson")

    Pearson's product-moment correlation

data:  country_level_indicator_descriptives$country_mean_confidence_healthcare and
country_level_indicator_descriptives$country_health_exp_per_GDP
t = 1.5341, df = 26, p-value = 0.1371
alternative hypothesis: true correlation is not equal to 0
95 percent confidence interval:
 -0.09521307  0.59700671
sample estimates:
      cor
0.2880976

> # 0.29, p = 0.137
> cor.test(country_level_indicator_descriptives$country_haq_index,
country_level_indicator_descriptives$country_health_exp_per_GDP, method = "pearson")

    Pearson's product-moment correlation
```

```
data:  country_level_indicator_descriptives$country_haq_index and
country_level_indicator_descriptives$country_health_exp_per_GDP
t = 4.8317, df = 26, p-value = 5.241e-05
alternative hypothesis: true correlation is not equal to 0
95 percent confidence interval:
 0.4233891 0.8442534
sample estimates:
      cor
0.6878202

> # 0.69, p < 0.0001
>
> # b) Individual-level
> cor.test(data_full$mean_confidence_healthcare, data_full$haq_index, method = "pearson")

    Pearson's product-moment correlation

data:  data_full$mean_confidence_healthcare and data_full$haq_index
t = 51.347, df = 26530, p-value < 2.2e-16
alternative hypothesis: true correlation is not equal to 0
95 percent confidence interval:
 0.2896712 0.3115617
sample estimates:
      cor
0.3006561

> # 0.30, p < 0.0001
> cor.test(data_full$mean_confidence_healthcare, data_full$health_exp_per_GDP, method = "pearson")

    Pearson's product-moment correlation

data:  data_full$mean_confidence_healthcare and data_full$health_exp_per_GDP
t = 43.599, df = 26530, p-value < 2.2e-16
alternative hypothesis: true correlation is not equal to 0
95 percent confidence interval:
 0.2473061 0.2697629
sample estimates:
      cor
0.2585694

> # 0.26, p < 0.0001
> cor.test(data_full$haq_index, data_full$health_exp_per_GDP, method = "pearson")

    Pearson's product-moment correlation

data:  data_full$haq_index and data_full$health_exp_per_GDP
t = 163.96, df = 26530, p-value < 2.2e-16
alternative hypothesis: true correlation is not equal to 0
95 percent confidence interval:
 0.7034016 0.7153561
sample estimates:
      cor
0.7094299

> # 0.71, p < 0.0001
>
>
> #################################################################
>
> ##### g) Tests for variation
>
> ## 1) Test for country-level variation (using proportions test (see https://sphweb.bumc.bu.edu/otlt/MPH-
Modules/BS/R/R6_CategoricalDataAnalysis/R6_CategoricalDataAnalysis6.html))
>
> # Calculate sums
> prop_test = country_level_indicator_descriptives%>%
+   mutate(country_haq_index_recoded = country_haq_index/100,
+          country_health_exp_per_GDP_recoded = country_health_exp_per_GDP/100,
+          country_mean_confidence_healthcare_recoded = scales::rescale(country_mean_confidence_healthcare, to =
c(0, 1)))%>%
+   mutate(sum_trust = round(country_mean_confidence_healthcare_recoded * num_resp, 0),
+          sum_haq = round(country_haq_index_recoded * num_resp, 0),
+          sum_exp = round(country_health_exp_per_GDP_recoded * num_resp, 0))
>
> prop.test(prop_test$sum_trust, prop_test$num_resp)
```

```
    28-sample test for equality of proportions without continuity correction

data:  prop_test$sum_trust out of prop_test$num_resp
X-squared = 7434, df = 27, p-value < 2.2e-16
alternative hypothesis: two.sided
sample estimates:
     prop 1      prop 2      prop 3      prop 4      prop 5      prop 6      prop 7      prop 8      prop 9 
prop 10     prop 11     prop 12     prop 13     prop 14     prop 15 
0.889898990 0.889698231 0.009307135 0.220502901 0.524017467 0.293293293 0.651068159 0.485744456 0.807291667 
0.752577320 0.366803279 0.000000000 0.137897782 0.357905983 0.238095238 
    prop 16     prop 17     prop 18     prop 19     prop 20     prop 21     prop 22     prop 23     prop 24 
prop 25     prop 26     prop 27     prop 28 
0.485744456 0.128258603 0.863157895 1.000000000 0.724798387 0.413114754 0.265822785 0.486542443 0.366764995 
0.642292490 0.651090343 0.724713243 0.614533965 

> # X-squared(27) = 7434, p < 0.001
> prop.test(prop_test$sum_haq, prop_test$num_resp)

    28-sample test for equality of proportions without continuity correction

data:  prop_test$sum_haq out of prop_test$num_resp
X-squared = 429.51, df = 27, p-value < 2.2e-16
alternative hypothesis: two.sided
sample estimates:
   prop 1    prop 2    prop 3    prop 4    prop 5    prop 6    prop 7    prop 8    prop 9   prop 10   prop 11 
prop 12   prop 13   prop 14   prop 15   prop 16   prop 17   prop 18 
0.7959596 0.7970864 0.6483971 0.7398453 0.7620087 0.7597598 0.7863683 0.6810982 0.7947917 0.7876289 0.7827869 
0.8120531 0.6943105 0.8012821 0.8157350 0.6948258 0.6840459 0.8084211 
  prop 19   prop 20   prop 21   prop 22   prop 23   prop 24   prop 25   prop 26   prop 27   prop 28 
0.7770563 0.8175403 0.6961749 0.7405063 0.6366460 0.7000983 0.7509881 0.7995846 0.8342023 0.7780411 

> # X-squared(27) = 429.51, p < 0.001
> prop.test(prop_test$sum_exp, prop_test$num_resp)

    28-sample test for equality of proportions without continuity correction

data:  prop_test$sum_exp out of prop_test$num_resp
X-squared = 78.711, df = 27, p-value = 5.913e-07
alternative hypothesis: two.sided
sample estimates:
    prop 1     prop 2     prop 3     prop 4     prop 5     prop 6     prop 7     prop 8     prop 9    prop 10 
prop 11    prop 12    prop 13    prop 14    prop 15    prop 16    prop 17 
0.10000000 0.08324662 0.05687694 0.07059961 0.05676856 0.06806807 0.08748728 0.06019007 0.08020833 0.10309278 
0.09972678 0.08478039 0.07425265 0.06944444 0.07867495 0.06230201 0.05839416 
   prop 18    prop 19    prop 20    prop 21    prop 22    prop 23    prop 24    prop 25    prop 26    prop 27 
prop 28 
0.06526316 0.06709957 0.07963710 0.05901639 0.08438819 0.04244306 0.06489676 0.07905138 0.07788162 0.08446298 
0.07424961 

> # X-squared(27) = 78.711, p < 0.001
> 
> 
> ## 2) Test for wave-level variation: Relationship between survey wave and macro-level indicators
> 
> # Doesn't work for trust, because only 2 time points
> 
> indicator_descriptives_across_HAQ_years = haq%>%
+   mutate(year_haq = as.numeric(as.character(year_haq)))%>%
+   group_by(year_haq)%>%
+   dplyr::summarise(wave_haq_index = round(mean(haq_index), 2))
> 
> indicator_descriptives_across_health_exp_years = health_exp%>%
+   mutate(year_health_exp = as.numeric(as.character(year_health_exp)))%>%
+   group_by(year_health_exp)%>%
+   dplyr::summarise(wave_health_exp_per_GDP = round(mean(health_exp_per_GDP), 2))
> 
> cor.test(indicator_descriptives_across_HAQ_years$wave_haq_index,
indicator_descriptives_across_HAQ_years$year_haq, method = "pearson")

    Pearson's product-moment correlation

data:  indicator_descriptives_across_HAQ_years$wave_haq_index and indicator_descriptives_across_HAQ_years$year_haq
t = 47.466, df = 4, p-value = 1.178e-06
alternative hypothesis: true correlation is not equal to 0
95 percent confidence interval:
 0.9915100 0.9999077
sample estimates:
```

```
      cor
0.9991135

> # 0.99, p < 0.001
>
> cor.test(indicator_descriptives_across_health_exp_years$wave_health_exp_per_GDP,
indicator_descriptives_across_health_exp_years$year_health_exp, method = "pearson")

    Pearson's product-moment correlation

data:  indicator_descriptives_across_health_exp_years$wave_health_exp_per_GDP and
indicator_descriptives_across_health_exp_years$year_health_exp
t = 16.577, df = 18, p-value = 2.392e-12
alternative hypothesis: true correlation is not equal to 0
95 percent confidence interval:
 0.9211591 0.9878163
sample estimates:
      cor
0.9687758

> # 0.97, p < 0.001
>
> ### Examine also trends at the country level
>
> # Trust
> evs_spread = evs_agg%>%
+   select(-iso_code_alpha2)%>%
+   mutate(year_EVS = ifelse(year_EVS == 1999, "y1999", "y2008"))%>%
+   spread(year_EVS, mean_confidence_healthcare)%>%
+   mutate(change = y2008 - y1999)
>
> # in 11/28 countries: increase in trust over time
> # in 17/28 countries: decrease in trust over time
>
> # HAQ
> haq_spread = haq%>%
+   select(-iso_code_alpha2)%>%
+   spread(year_haq, haq_index)
>
> # For all countries but 3 (Lithuania, Estonia, Latvia) continuous increase in HAQ index over time
> # For all countries continuous increase from 1995 onwards
>
> # Health exp
> health_exp_spread = health_exp%>%
+   select(-iso_code_alpha2, -country_name_alt)%>%
+   spread(year_health_exp, health_exp_per_GDP)
>
> # Quite some fluctuation across individual years
> # When examining smoothed relationship of time on health_exp, all countries show positive trend (most constant
relationship in Estonia)
> #################################################################
>
> ####### 3) Mixed-effects models
>
> # Due to problems with convergence in some models, we normalized the continuous variables (age, education)
> # (see https://rstudio-pubs-static.s3.amazonaws.com/33653_57fc7b8e5d484c909b615d8633c01d51.html)
> data_full$age_std = as.numeric(scale(data_full$age))
> data_full$education_std = as.numeric(scale(data_full$education))
> data_full$haq_index_std = as.numeric(scale(data_full$haq_index))
> data_full$health_exp_per_GDP_std = as.numeric(scale(data_full$health_exp_per_GDP))
> data_full$mean_confidence_healthcare_std = as.numeric(scale(data_full$mean_confidence_healthcare))
>
> # Convert year variables to factor
> data_full$year_health_exp = as.factor(data_full$year_health_exp)
> data_full$year_haq = as.factor(data_full$year_haq)
> data_full$year_EVS = as.factor(data_full$year_EVS)
>
> ##### a) Intercept-only models (empty models)
>
> # 0) Only including random effect of country
> m0 = glmer(donate_blood ~ (1|country), data = data_full, family = "binomial")
> summary(m0)
Generalized linear mixed model fit by maximum likelihood (Laplace Approximation) ['glmerMod']
 Family: binomial  ( logit )
Formula: donate_blood ~ (1 | country)
   Data: data_full
```

```
     AIC      BIC   logLik deviance df.resid
 34848.6  34864.9 -17422.3  34844.6    26530

Scaled residuals:
    Min      1Q  Median      3Q     Max
-1.0471 -0.8071 -0.6672  1.1865  1.8035

Random effects:
 Groups  Name        Variance Std.Dev.
 country (Intercept) 0.1008   0.3175
Number of obs: 26532, groups:  country, 28

Fixed effects:
            Estimate Std. Error z value Pr(>|z|)
(Intercept) -0.48195    0.06138  -7.852  4.1e-15 ***
---
Signif. codes:  0 ‘***’ 0.001 ‘**’ 0.01 ‘*’ 0.05 ‘.’ 0.1 ‘ ’ 1
> # AIC = 34848.6
>
> # 0A) Only including random effect of time (EVS)
> m0a = glmer(donate_blood ~ (1|year_EVS), data = data_full, family = "binomial")
> summary(m0a)
Generalized linear mixed model fit by maximum likelihood (Laplace Approximation) ['glmerMod']
 Family: binomial  ( logit )
Formula: donate_blood ~ (1 | year_EVS)
   Data: data_full

     AIC      BIC   logLik deviance df.resid
 35205.7  35222.1 -17600.9  35201.7    26530

Scaled residuals:
    Min      1Q  Median      3Q     Max
-0.8299 -0.8299 -0.6981  1.2050  1.4325

Random effects:
 Groups   Name        Variance Std.Dev.
 year_EVS (Intercept) 0.03031  0.1741
Number of obs: 26532, groups:  year_EVS, 2

Fixed effects:
            Estimate Std. Error z value Pr(>|z|)
(Intercept)   -0.546      0.123  -4.438 9.08e-06 ***
---
Signif. codes:  0 ‘***’ 0.001 ‘**’ 0.01 ‘*’ 0.05 ‘.’ 0.1 ‘ ’ 1
> # AIC = 35205.7
>
> # 0B) Only including random effect of time (HAQ)
> m0b = glmer(donate_blood ~ (1|year_haq), data = data_full, family = "binomial")
> summary(m0b)
Generalized linear mixed model fit by maximum likelihood (Laplace Approximation) ['glmerMod']
 Family: binomial  ( logit )
Formula: donate_blood ~ (1 | year_haq)
   Data: data_full

     AIC      BIC   logLik deviance df.resid
 35136.0  35152.4 -17566.0  35132.0    26530

Scaled residuals:
    Min      1Q  Median      3Q     Max
-0.8627 -0.8080 -0.6937  1.1795  1.9381

Random effects:
 Groups   Name        Variance Std.Dev.
 year_haq (Intercept) 0.1251   0.3537
Number of obs: 26532, groups:  year_haq, 6

Fixed effects:
            Estimate Std. Error z value Pr(>|z|)
(Intercept)  -0.6133     0.1446   -4.24 2.23e-05 ***
---
Signif. codes:  0 ‘***’ 0.001 ‘**’ 0.01 ‘*’ 0.05 ‘.’ 0.1 ‘ ’ 1
> # AIC = 35136.0
>
> # 0C) Only including random effect of time (health_exp)
> m0c = glmer(donate_blood ~ (1|year_health_exp), data = data_full, family = "binomial")
> summary(m0c)
Generalized linear mixed model fit by maximum likelihood (Laplace Approximation) ['glmerMod']
```

```
 Family: binomial  ( logit )
Formula: donate_blood ~ (1 | year_health_exp)
   Data: data_full

     AIC      BIC   logLik deviance df.resid
 35167.3  35183.6 -17581.6  35163.3    26530

Scaled residuals:
    Min      1Q  Median      3Q     Max
-0.8622 -0.8192 -0.7254  1.2207  1.9964

Random effects:
 Groups          Name        Variance Std.Dev.
 year_health_exp (Intercept) 0.07897  0.281
Number of obs: 26532, groups:  year_health_exp, 20

Fixed effects:
            Estimate Std. Error z value Pr(>|z|)
(Intercept)  -0.5838     0.0657  -8.887   <2e-16 ***
---
Signif. codes:  0 ‘***’ 0.001 ‘**’ 0.01 ‘*’ 0.05 ‘.’ 0.1 ‘ ’ 1
> # AIC = 35167.3
>
> # Significance of random effects
> m0_constrained = glm(donate_blood ~ 1, data = data_full, family = "binomial")
> summary(m0_constrained)

Call:
glm(formula = donate_blood ~ 1, family = "binomial", data = data_full)

Coefficients:
            Estimate Std. Error z value Pr(>|z|)
(Intercept) -0.47154    0.01262  -37.36   <2e-16 ***
---
Signif. codes:  0 ‘***’ 0.001 ‘**’ 0.01 ‘*’ 0.05 ‘.’ 0.1 ‘ ’ 1

(Dispersion parameter for binomial family taken to be 1)

    Null deviance: 35346  on 26531  degrees of freedom
Residual deviance: 35346  on 26531  degrees of freedom
AIC: 35348

Number of Fisher Scoring iterations: 4

> # AIC = 35348
>
> anova(m0, m0_constrained)
Data: data_full
Models:
m0_constrained: donate_blood ~ 1
m0: donate_blood ~ (1 | country)
               npar   AIC   BIC logLik deviance  Chisq Df Pr(>Chisq)
m0_constrained    1 35348 35357 -17673    35346
m0                2 34849 34865 -17422    34845 501.79  1  < 2.2e-16 ***
---
Signif. codes:  0 ‘***’ 0.001 ‘**’ 0.01 ‘*’ 0.05 ‘.’ 0.1 ‘ ’ 1
> # LL-test significant: country random effect improves model fit
> # chi^2(1) = 501.79, p < 0.001
>
> anova(m0a, m0_constrained)
Data: data_full
Models:
m0_constrained: donate_blood ~ 1
m0a: donate_blood ~ (1 | year_EVS)
               npar   AIC   BIC logLik deviance Chisq Df Pr(>Chisq)
m0_constrained    1 35348 35357 -17673    35346
m0a               2 35206 35222 -17601    35202 144.6  1  < 2.2e-16 ***
---
Signif. codes:  0 ‘***’ 0.001 ‘**’ 0.01 ‘*’ 0.05 ‘.’ 0.1 ‘ ’ 1
> # LL-test significant: time (EVS) random effect improves model fit
> # chi^2(1) = 144.6, p < 0.001
>
> anova(m0b, m0_constrained)
Data: data_full
Models:
m0_constrained: donate_blood ~ 1
m0b: donate_blood ~ (1 | year_haq)
```

```
                npar   AIC   BIC logLik deviance  Chisq Df Pr(>Chisq)    
m0_constrained     1 35348 35357 -17673    35346                         
m0b                2 35136 35152 -17566    35132 214.33  1  < 2.2e-16 ***
---
Signif. codes:  0 ‘***’ 0.001 ‘**’ 0.01 ‘*’ 0.05 ‘.’ 0.1 ‘ ’ 1
> # LL-test significant: time (HAQ) random effect improves model fit
> # chi^2(1) = 214.33, p < 0.001
> 
> anova(m0c, m0_constrained)
Data: data_full
Models:
m0_constrained: donate_blood ~ 1
m0c: donate_blood ~ (1 | year_health_exp)
                npar   AIC   BIC logLik deviance  Chisq Df Pr(>Chisq)    
m0_constrained     1 35348 35357 -17673    35346                         
m0c                2 35167 35184 -17582    35163 183.07  1  < 2.2e-16 ***
---
Signif. codes:  0 ‘***’ 0.001 ‘**’ 0.01 ‘*’ 0.05 ‘.’ 0.1 ‘ ’ 1
> # LL-test significant: time (health exp) random effect improves model fit
> # chi^2(1) = 183.07, p < 0.001
> 
> 
> ##### b) Demographics
> 
> m1 = glmer(donate_blood ~ age_std + gender + education_std + partner_status + employment_status + 
parental_status + type_of_community + (1|country), data = data_full, family = "binomial")
> summary(m1)
Generalized linear mixed model fit by maximum likelihood (Laplace Approximation) ['glmerMod']
 Family: binomial  ( logit )
Formula: donate_blood ~ age_std + gender + education_std + partner_status +      employment_status + 
parental_status + type_of_community +      (1 | country)
   Data: data_full

     AIC      BIC   logLik deviance df.resid 
 33481.3  33563.0 -16730.6  33461.3    26009 

Scaled residuals: 
    Min      1Q  Median      3Q     Max 
-2.6927 -0.7931 -0.6102  1.1000  3.1839 

Random effects:
 Groups  Name        Variance Std.Dev.
 country (Intercept) 0.09398  0.3066  
Number of obs: 26019, groups:  country, 28

Fixed effects:
                   Estimate Std. Error z value Pr(>|z|)    
(Intercept)        -0.43908    0.06898  -6.365 1.95e-10 ***
age_std             0.21627    0.01616  13.380  < 2e-16 ***
gender1            -0.44880    0.02658 -16.882  < 2e-16 ***
education_std       0.18230    0.01461  12.479  < 2e-16 ***
partner_status1     0.11075    0.02893   3.828 0.000129 ***
employment_status1  0.29899    0.02988  10.006  < 2e-16 ***
parental_status1    0.03518    0.03742   0.940 0.347192    
type_of_community1 -0.02327    0.03295  -0.706 0.479960    
type_of_community2 -0.05366    0.03535  -1.518 0.129037    
---
Signif. codes:  0 ‘***’ 0.001 ‘**’ 0.01 ‘*’ 0.05 ‘.’ 0.1 ‘ ’ 1

Correlation of Fixed Effects:
            (Intr) ag_std gendr1 edctn_ prtn_1 empl_1 prnt_1 typ__1
age_std     -0.075                                                 
gender1     -0.242 -0.029                                          
educatn_std -0.005  0.148 -0.022                                   
prtnr_stts1 -0.234 -0.110  0.099 -0.025                            
emplymnt_s1 -0.190  0.388  0.059 -0.075 -0.139                     
prntl_stts1 -0.015  0.340 -0.079 -0.003 -0.213 -0.025              
typ_f_cmmn1 -0.281 -0.035  0.007  0.058 -0.031  0.001 -0.019       
typ_f_cmmn2 -0.258 -0.038  0.007  0.107 -0.048  0.005 -0.030  0.563
> print(summary(m1),digits=2)
Generalized linear mixed model fit by maximum likelihood (Laplace Approximation) ['glmerMod']
 Family: binomial  ( logit )
Formula: donate_blood ~ age_std + gender + education_std + partner_status +      employment_status + 
parental_status + type_of_community +      (1 | country)
   Data: data_full

     AIC      BIC   logLik deviance df.resid 
```

```
 33481.3  33563.0 -16730.6  33461.3    26009

Scaled residuals: 
   Min     1Q Median     3Q    Max 
 -2.69  -0.79  -0.61   1.10   3.18 

Random effects:
 Groups  Name        Variance Std.Dev.
 country (Intercept) 0.094    0.31    
Number of obs: 26019, groups:  country, 28

Fixed effects:
                   Estimate Std. Error z value Pr(>|z|)    
(Intercept)          -0.439      0.069    -6.4    2e-10 ***
age_std               0.216      0.016    13.4   <2e-16 ***
gender1              -0.449      0.027   -16.9   <2e-16 ***
education_std         0.182      0.015    12.5   <2e-16 ***
partner_status1       0.111      0.029     3.8    1e-04 ***
employment_status1    0.299      0.030    10.0   <2e-16 ***
parental_status1      0.035      0.037     0.9      0.3    
type_of_community1   -0.023      0.033    -0.7      0.5    
type_of_community2   -0.054      0.035    -1.5      0.1    
---
Signif. codes:  0 '***' 0.001 '**' 0.01 '*' 0.05 '.' 0.1 ' ' 1

Correlation of Fixed Effects:
            (Intr) ag_std gendr1 edctn_ prtn_1 empl_1 prnt_1 typ__1
age_std     -0.075                                                 
gender1     -0.242 -0.029                                          
educatn_std -0.005  0.148 -0.022                                   
prtnr_stts1 -0.234 -0.110  0.099 -0.025                            
emplymnt_s1 -0.190  0.388  0.059 -0.075 -0.139                     
prntl_stts1 -0.015  0.340 -0.079 -0.003 -0.213 -0.025              
typ_f_cmmn1 -0.281 -0.035  0.007  0.058 -0.031  0.001 -0.019       
typ_f_cmmn2 -0.258 -0.038  0.007  0.107 -0.048  0.005 -0.030  0.563
> # AIC = 33481.3 (< 34848.6 --> demographics improve model fit)
> # significant effects: age (older more), gender (male more), education (higher education more), partner_status (with partner more), employment_status1 (employed more)
> 
> 
> ##### c) Trust in healthcare system
> 
> m2 = glmer(donate_blood ~ age_std + gender + education_std + partner_status + employment_status + parental_status + type_of_community + mean_confidence_healthcare_std + (1|country) + (1|year_EVS), data = data_full, family = "binomial")
> summary(m2)
Generalized linear mixed model fit by maximum likelihood (Laplace Approximation) ['glmerMod']
 Family: binomial  ( logit )
Formula: donate_blood ~ age_std + gender + education_std + partner_status +      employment_status + parental_status + type_of_community +  
    mean_confidence_healthcare_std + (1 | country) + (1 | year_EVS)
   Data: data_full

     AIC      BIC   logLik deviance df.resid 
 33431.3  33529.3 -16703.7  33407.3    26007 

Scaled residuals: 
    Min      1Q  Median      3Q     Max 
-2.6259 -0.7891 -0.6052  1.0927  3.2852 

Random effects:
 Groups   Name        Variance Std.Dev.
 country  (Intercept) 0.09919  0.3149  
 year_EVS (Intercept) 0.02748  0.1658  
Number of obs: 26019, groups:  country, 28; year_EVS, 2

Fixed effects:
                               Estimate Std. Error z value Pr(>|z|)    
(Intercept)                    -0.47803    0.13683  -3.494 0.000476 ***
age_std                         0.09421    0.02449   3.847 0.000120 ***
gender1                        -0.45641    0.02664 -17.130  < 2e-16 ***
education_std                   0.17807    0.01463  12.169  < 2e-16 ***
partner_status1                 0.09236    0.02915   3.169 0.001531 ** 
employment_status1              0.25084    0.03077   8.152 3.58e-16 ***
parental_status1                0.08710    0.03825   2.277 0.022779 *  
type_of_community1             -0.02723    0.03300  -0.825 0.409414    
type_of_community2             -0.06133    0.03542  -1.731 0.083370 .  
```

```
mean_confidence_healthcare_std  0.11376    0.03318   3.429 0.000607 ***
---
Signif. codes:  0 ‘***’ 0.001 ‘**’ 0.01 ‘*’ 0.05 ‘.’ 0.1 ‘ ’ 1

Correlation of Fixed Effects:
            (Intr) ag_std gendr1 edctn_ prtn_1 empl_1 prnt_1 typ__1 typ__2
age_std      0.004
gender1     -0.120  0.017
educatn_std -0.001  0.108 -0.020
prtnr_stts1 -0.113  0.010  0.103 -0.024
emplymnt_s1 -0.084  0.428  0.068 -0.070 -0.107
prntl_stts1 -0.016  0.073 -0.086 -0.007 -0.228 -0.071
typ_f_cmmn1 -0.142 -0.017  0.007  0.059 -0.030  0.002 -0.020
typ_f_cmmn2 -0.129 -0.009  0.008  0.108 -0.046  0.010 -0.033  0.564
mn_cnfdnc__ -0.010  0.007 -0.002 -0.044  0.033  0.014  0.011 -0.015 -0.019
> print(summary(m2),digits=2)
Generalized linear mixed model fit by maximum likelihood (Laplace Approximation) ['glmerMod']
 Family: binomial  ( logit )
Formula: donate_blood ~ age_std + gender + education_std + partner_status +      employment_status +
parental_status + type_of_community +
    mean_confidence_healthcare_std + (1 | country) + (1 | year_EVS)
   Data: data_full

     AIC      BIC   logLik deviance df.resid
 33431.3  33529.3 -16703.7  33407.3    26007

Scaled residuals:
   Min     1Q Median     3Q    Max
 -2.63  -0.79  -0.61   1.09   3.29

Random effects:
 Groups   Name        Variance Std.Dev.
 country  (Intercept) 0.099    0.31
 year_EVS (Intercept) 0.027    0.17
Number of obs: 26019, groups:  country, 28; year_EVS, 2

Fixed effects:
                               Estimate Std. Error z value Pr(>|z|)
(Intercept)                      -0.478      0.137    -3.5    5e-04 ***
age_std                           0.094      0.024     3.8    1e-04 ***
gender1                          -0.456      0.027   -17.1   <2e-16 ***
education_std                     0.178      0.015    12.2   <2e-16 ***
partner_status1                   0.092      0.029     3.2    0.002 **
employment_status1                0.251      0.031     8.2    4e-16 ***
parental_status1                  0.087      0.038     2.3    0.023 *
type_of_community1               -0.027      0.033    -0.8    0.409
type_of_community2               -0.061      0.035    -1.7    0.083 .
mean_confidence_healthcare_std    0.114      0.033     3.4    6e-04 ***
---
Signif. codes:  0 ‘***’ 0.001 ‘**’ 0.01 ‘*’ 0.05 ‘.’ 0.1 ‘ ’ 1

Correlation of Fixed Effects:
            (Intr) ag_std gendr1 edctn_ prtn_1 empl_1 prnt_1 typ__1 typ__2
age_std      0.004
gender1     -0.120  0.017
educatn_std -0.001  0.108 -0.020
prtnr_stts1 -0.113  0.010  0.103 -0.024
emplymnt_s1 -0.084  0.428  0.068 -0.070 -0.107
prntl_stts1 -0.016  0.073 -0.086 -0.007 -0.228 -0.071
typ_f_cmmn1 -0.142 -0.017  0.007  0.059 -0.030  0.002 -0.020
typ_f_cmmn2 -0.129 -0.009  0.008  0.108 -0.046  0.010 -0.033  0.564
mn_cnfdnc__ -0.010  0.007 -0.002 -0.044  0.033  0.014  0.011 -0.015 -0.019
> # significant effects: mean_confidence_healthcare (higher trust MORE)
> # AIC: 33431.3
>
>
> ##### d) Healthcare quality
>
> m3 = glmer(donate_blood ~ age_std + gender + education_std + partner_status + employment_status +
parental_status + type_of_community + haq_index_std + health_exp_per_GDP_std + (1|country) + (1|year_haq) +
(1|year_health_exp), data = data_full, family = "binomial")
> summary(m3)
Generalized linear mixed model fit by maximum likelihood (Laplace Approximation) ['glmerMod']
 Family: binomial  ( logit )
Formula: donate_blood ~ age_std + gender + education_std + partner_status +      employment_status +
parental_status + type_of_community +
    haq_index_std + health_exp_per_GDP_std + (1 | country) +      (1 | year_haq) + (1 | year_health_exp)
```

```
   Data: data_full

     AIC      BIC   logLik deviance df.resid
 33424.7  33539.0 -16698.3  33396.7    26005

Scaled residuals:
    Min      1Q  Median      3Q     Max
-2.4699 -0.7923 -0.6048  1.0909  3.0164

Random effects:
 Groups          Name        Variance Std.Dev.
 country         (Intercept) 0.101364 0.31838
 year_health_exp (Intercept) 0.002846 0.05335
 year_haq        (Intercept) 0.250663 0.50066
Number of obs: 26019, groups:  country, 28; year_health_exp, 20; year_haq, 6

Fixed effects:
                       Estimate Std. Error z value Pr(>|z|)
(Intercept)            -0.61497    0.22049  -2.789  0.00528 **
age_std                -0.11275    0.05997  -1.880  0.06009 .
gender1                -0.46373    0.02674 -17.342  < 2e-16 ***
education_std           0.17419    0.01465  11.894  < 2e-16 ***
partner_status1         0.05863    0.02979   1.968  0.04904 *
employment_status1      0.21825    0.03403   6.413 1.43e-10 ***
parental_status1        0.08246    0.04057   2.032  0.04212 *
type_of_community1     -0.02709    0.03303  -0.820  0.41209
type_of_community2     -0.06373    0.03545  -1.798  0.07218 .
haq_index_std           0.10137    0.05643   1.796  0.07242 .
health_exp_per_GDP_std -0.01979    0.04086  -0.484  0.62808
---
Signif. codes:  0 ‘***’ 0.001 ‘**’ 0.01 ‘*’ 0.05 ‘.’ 0.1 ‘ ’ 1

Correlation of Fixed Effects:
            (Intr) ag_std gendr1 edctn_ prtn_1 empl_1 prnt_1 typ__1 typ__2 hq_nd_
age_std      0.136
gender1     -0.074  0.021
educatn_std  0.006  0.074 -0.015
prtnr_stts1 -0.051  0.141  0.113 -0.010
emplymnt_s1 -0.070  0.098  0.091 -0.041 -0.046
prntl_stts1 -0.029 -0.046 -0.071  0.006 -0.200  0.019
typ_f_cmmn1 -0.087 -0.002  0.008  0.059 -0.027  0.008 -0.018
typ_f_cmmn2 -0.076  0.017  0.009  0.107 -0.040  0.013 -0.033  0.563
haq_ndx_std -0.085 -0.057 -0.003 -0.034 -0.011 -0.014  0.001 -0.001 -0.012
hlth___GDP_ -0.011 -0.026 -0.004 -0.006  0.025 -0.009  0.033 -0.019 -0.002 -0.218
> print(summary(m3),digits=2)
Generalized linear mixed model fit by maximum likelihood (Laplace Approximation) ['glmerMod']
 Family: binomial  ( logit )
Formula: donate_blood ~ age_std + gender + education_std + partner_status +      employment_status +
parental_status + type_of_community +
    haq_index_std + health_exp_per_GDP_std + (1 | country) +      (1 | year_haq) + (1 | year_health_exp)
   Data: data_full

     AIC      BIC   logLik deviance df.resid
 33424.7  33539.0 -16698.3  33396.7    26005

Scaled residuals:
   Min     1Q Median     3Q    Max
 -2.47  -0.79  -0.60   1.09   3.02

Random effects:
 Groups          Name        Variance Std.Dev.
 country         (Intercept) 0.1014   0.318
 year_health_exp (Intercept) 0.0028   0.053
 year_haq        (Intercept) 0.2507   0.501
Number of obs: 26019, groups:  country, 28; year_health_exp, 20; year_haq, 6

Fixed effects:
                       Estimate Std. Error z value Pr(>|z|)
(Intercept)              -0.615      0.220    -2.8    0.005 **
age_std                  -0.113      0.060    -1.9    0.060 .
gender1                  -0.464      0.027   -17.3   <2e-16 ***
education_std             0.174      0.015    11.9   <2e-16 ***
partner_status1           0.059      0.030     2.0    0.049 *
employment_status1        0.218      0.034     6.4    1e-10 ***
parental_status1          0.082      0.041     2.0    0.042 *
type_of_community1       -0.027      0.033    -0.8    0.412
type_of_community2       -0.064      0.035    -1.8    0.072 .
```

```
haq_index_std             0.101      0.056     1.8    0.072 .
health_exp_per_GDP_std   -0.020      0.041    -0.5    0.628
---
Signif. codes:  0 '***' 0.001 '**' 0.01 '*' 0.05 '.' 0.1 ' ' 1

Correlation of Fixed Effects:
            (Intr) ag_std gendr1 edctn_ prtn_1 empl_1 prnt_1 typ__1 typ__2 hq_nd_
age_std      0.136
gender1     -0.074  0.021
educatn_std  0.006  0.074 -0.015
prtnr_stts1 -0.051  0.141  0.113 -0.010
emplymnt_s1 -0.070  0.098  0.091 -0.041 -0.046
prntl_stts1 -0.029 -0.046 -0.071  0.006 -0.200  0.019
typ_f_cmmn1 -0.087 -0.002  0.008  0.059 -0.027  0.008 -0.018
typ_f_cmmn2 -0.076  0.017  0.009  0.107 -0.040  0.013 -0.033  0.563
haq_ndx_std -0.085 -0.057 -0.003 -0.034 -0.011 -0.014  0.001 -0.001 -0.012
hlth___GDP_ -0.011 -0.026 -0.004 -0.006  0.025 -0.009  0.033 -0.019 -0.002 -0.218
> # MARGINALLY significant effect: haq_index (higher HAQ MORE)
> # AIC: 33424.7
>
>
> ## a) Only HAQ
> m3a = glmer(donate_blood ~ haq_index_std + age_std + gender + education_std + partner_status + employment_status
+ parental_status + type_of_community + (1|country) + (1|year_haq), data = data_full, family = "binomial")
> summary(m3a)
Generalized linear mixed model fit by maximum likelihood (Laplace Approximation) ['glmerMod']
 Family: binomial  ( logit )
Formula: donate_blood ~ haq_index_std + age_std + gender + education_std +      partner_status + employment_status
+ parental_status + type_of_community +
    (1 | country) + (1 | year_haq)
   Data: data_full

     AIC      BIC   logLik deviance df.resid
 33422.3  33520.3 -16699.1  33398.3    26007

Scaled residuals:
    Min      1Q  Median      3Q     Max
-2.4864 -0.7908 -0.6054  1.0911  2.9907

Random effects:
 Groups   Name        Variance Std.Dev.
 country  (Intercept) 0.09949  0.3154
 year_haq (Intercept) 0.23175  0.4814
Number of obs: 26019, groups:  country, 28; year_haq, 6

Fixed effects:
                   Estimate Std. Error z value Pr(>|z|)
(Intercept)        -0.59347    0.21169  -2.803  0.00506 **
haq_index_std       0.09390    0.05503   1.706  0.08792 .
age_std            -0.07709    0.05082  -1.517  0.12933
gender1            -0.46302    0.02672 -17.327  < 2e-16 ***
education_std       0.17451    0.01464  11.921  < 2e-16 ***
partner_status1     0.06274    0.02956   2.122  0.03381 *
employment_status1  0.21978    0.03379   6.504  7.8e-11 ***
parental_status1    0.07649    0.03993   1.916  0.05538 .
type_of_community1 -0.02711    0.03301  -0.821  0.41150
type_of_community2 -0.06300    0.03543  -1.778  0.07539 .
---
Signif. codes:  0 '***' 0.001 '**' 0.01 '*' 0.05 '.' 0.1 ' ' 1

Correlation of Fixed Effects:
            (Intr) hq_nd_ ag_std gendr1 edctn_ prtn_1 empl_1 prnt_1 typ__1
haq_ndx_std -0.092
age_std      0.112 -0.065
gender1     -0.079 -0.004  0.008
educatn_std  0.004 -0.036  0.075 -0.016
prtnr_stts1 -0.063 -0.004  0.096  0.111 -0.012
emplymnt_s1 -0.076 -0.016  0.077  0.089 -0.044 -0.057
prntl_stts1 -0.019  0.006  0.018 -0.070  0.008 -0.195  0.018
typ_f_cmmn1 -0.092 -0.005 -0.007  0.008  0.059 -0.027  0.008 -0.017
typ_f_cmmn2 -0.081 -0.012  0.009  0.009  0.107 -0.042  0.013 -0.031  0.563
> # MARGINALLY significant effect: haq_index (higher HAQ MORE)
>
> ## b) Only Health exp
> m3b = glmer(donate_blood ~ health_exp_per_GDP_std + age_std + gender + education_std + partner_status +
employment_status + parental_status + type_of_community + (1|country) + (1|year_health_exp), data = data_full,
family = "binomial")
```

```
> summary(m3b)
Generalized linear mixed model fit by maximum likelihood (Laplace Approximation) ['glmerMod']
 Family: binomial  ( logit )
Formula: donate_blood ~ health_exp_per_GDP_std + age_std + gender + education_std +      partner_status +
employment_status + parental_status + type_of_community +
    (1 | country) + (1 | year_health_exp)
   Data: data_full

     AIC      BIC   logLik deviance df.resid
 33447.7  33545.7 -16711.8  33423.7    26007

Scaled residuals:
    Min      1Q  Median      3Q     Max
-2.5137 -0.7918 -0.6060  1.0923  3.1229

Random effects:
 Groups          Name        Variance Std.Dev.
 country         (Intercept) 0.10148  0.3186
 year_health_exp (Intercept) 0.08674  0.2945
Number of obs: 26019, groups:  country, 28; year_health_exp, 20

Fixed effects:
                       Estimate Std. Error z value Pr(>|z|)
(Intercept)            -0.57791    0.10391  -5.562 2.67e-08 ***
health_exp_per_GDP_std -0.02145    0.04196  -0.511   0.6092
age_std                -0.05169    0.04964  -1.041   0.2978
gender1                -0.46301    0.02675 -17.306  < 2e-16 ***
education_std           0.17595    0.01465  12.010  < 2e-16 ***
partner_status1         0.06422    0.02994   2.145   0.0320 *
employment_status1      0.21770    0.03411   6.383 1.74e-10 ***
parental_status1        0.08401    0.04074   2.062   0.0392 *
type_of_community1     -0.02630    0.03303  -0.796   0.4259
type_of_community2     -0.06197    0.03545  -1.748   0.0804 .
---
Signif. codes:  0 ‘***’ 0.001 ‘**’ 0.01 ‘*’ 0.05 ‘.’ 0.1 ‘ ’ 1

Correlation of Fixed Effects:
            (Intr) h___GD ag_std gendr1 edctn_ prtn_1 empl_1 prnt_1 typ__1
hlth___GDP_ -0.114
age_std      0.273 -0.093
gender1     -0.153 -0.011  0.045
educatn_std  0.003 -0.017  0.089 -0.014
prtnr_stts1 -0.107  0.005  0.169  0.117 -0.007
emplymnt_s1 -0.107 -0.045  0.321  0.096 -0.032 -0.014
prntl_stts1 -0.081  0.041 -0.016 -0.071  0.007 -0.192  0.019
typ_f_cmmn1 -0.181 -0.027  0.001  0.009  0.059 -0.027  0.008 -0.020
typ_f_cmmn2 -0.161 -0.009  0.021  0.010  0.107 -0.039  0.016 -0.033  0.563
> # not significant
>
>
> ################################################################
>
> ####### 4) Predicted probabilities and plotting
>
> # Prediction intervals are generated based on tutorial here: https://cran.r-
project.org/web/packages/merTools/vignettes/Using_predictInterval.html
>
> library("merTools")
Loading required package: arm
Loading required package: MASS

Attaching package: ‘MASS’

The following object is masked from ‘package:dplyr’:

    select


arm (Version 1.13-1, built: 2022-8-25)

Working directory is /Users/caroline/Desktop/PhD/P2_medical_trust/analysis


Attaching package: ‘arm’

The following object is masked from ‘package:corrplot’:
```

```
    corrplot

The following objects are masked from ‘package:psych’:

    logit, rescale, sim

The following object is masked from ‘package:scales’:

    rescale


Attaching package: ‘merTools’

The following object is masked from ‘package:psych’:

    ICC

>
> ##### a) Trust
>
> newdata <- expand.grid(mean_confidence_healthcare_std = sort(unique(data_full$mean_confidence_healthcare_std)),
+                        age_std = mean(data_full$age_std, na.rm=TRUE),
+                        gender = "1",
+                        education_std = mean(data_full$education_std, na.rm=TRUE),
+                        partner_status="1",
+                        employment_status = "1",
+                        parental_status = "0",
+                        type_of_community = "1",
+                        country = "new country",
+                        year_EVS = "new year")
>
> PI <- predictInterval(merMod = m2, newdata = newdata, level = 0.8, n.sims = 1000, stat = "mean",
type="probability", include.resid.var = F)
Warning messages:
1:      The following levels of country from newdata
 -- new country -- are not in the model data.
     Currently, predictions for these values are based only on the
 fixed coefficients and the observation-level error.
2:      The following levels of year_EVS from newdata
 -- new year -- are not in the model data.
     Currently, predictions for these values are based only on the
 fixed coefficients and the observation-level error.
>
> data_with_PI = bind_cols(newdata, PI)
>
> a_with_CI = ggplot(data_with_PI, aes(x = mean_confidence_healthcare_std, y=fit, ymin=lwr, ymax=upr)) +
+   geom_smooth(aes(ymin = lwr, ymax = upr), stat = "identity") +
+   labs(y = "Predicted probability of blood donation", x = "Trust in the healthcare system (normalized)") +
+   theme(legend.position="bottom", plot.title = element_text(hjust = 0.5))+
+   expand_limits(y = 0)
>
>
> ##### b) HAQ
>
> newdata <- expand.grid(haq_index_std = sort(unique(data_full$haq_index_std)),
+                        age_std = mean(data_full$age_std, na.rm=TRUE),
+                        gender = "1",
+                        education_std = mean(data_full$education_std, na.rm=TRUE),
+                        partner_status="1",
+                        employment_status = "1",
+                        parental_status = "0",
+                        type_of_community = "1",
+                        country = "new country",
+                        year_haq = "new year")
>
> PI <- predictInterval(merMod = m3a, newdata = newdata, level = 0.8, n.sims = 1000, stat = "mean",
type="probability", include.resid.var = F)
Warning messages:
1:      The following levels of country from newdata
 -- new country -- are not in the model data.
     Currently, predictions for these values are based only on the
 fixed coefficients and the observation-level error.
2:      The following levels of year_haq from newdata
 -- new year -- are not in the model data.
     Currently, predictions for these values are based only on the
 fixed coefficients and the observation-level error.
>
```

```
> data_with_PI = bind_cols(newdata, PI)
>
> b_with_CI = ggplot(data_with_PI, aes(x = haq_index_std, y=fit, ymin=lwr, ymax=upr)) +
+   geom_smooth(aes(ymin = lwr, ymax = upr), stat = "identity") +
+   labs(y = "Predicted probability of blood donation", x = "HAQ index (normalized)") +
+   theme(legend.position="bottom", plot.title = element_text(hjust = 0.5))+
+   expand_limits(y = 0)
>
>
> ##### c) Healthcare expenditures
>
> newdata <- expand.grid(health_exp_per_GDP_std = sort(unique(data_full$health_exp_per_GDP_std)),
+                        age_std = mean(data_full$age_std, na.rm=TRUE),
+                        gender = "1",
+                        education_std = mean(data_full$education_std, na.rm=TRUE),
+                        partner_status="1",
+                        employment_status = "1",
+                        parental_status = "0",
+                        type_of_community = "1",
+                        country = "new country",
+                        year_health_exp = "new year")
>
> PI <- predictInterval(merMod = m3b, newdata = newdata, level = 0.8, n.sims = 1000, stat = "mean",
type="probability", include.resid.var = F)
Warning messages:
1:      The following levels of country from newdata
 -- new country -- are not in the model data.
     Currently, predictions for these values are based only on the
 fixed coefficients and the observation-level error.
2:      The following levels of year_health_exp from newdata
 -- new year -- are not in the model data.
     Currently, predictions for these values are based only on the
 fixed coefficients and the observation-level error.
>
> data_with_PI = bind_cols(newdata, PI)
>
> c_with_CI = ggplot(data_with_PI, aes(x = health_exp_per_GDP_std, y=fit, ymin=lwr, ymax=upr)) +
+   geom_smooth(aes(ymin = lwr, ymax = upr), stat = "identity") +
+   labs(y = "Predicted probability of blood donation", x = "Healthcare expenditures (normalized)") +
+   theme(legend.position="bottom", plot.title = element_text(hjust = 0.5))+
+   expand_limits(y = 0)
>
>
> # make combined plot for paper
> ggarrange(a_with_CI, b_with_CI, c_with_CI,
+           labels = c("A", "B", "C"),
+           ncol = 3, nrow = 1)
>
> #ggsave("plots/scatter/combined_pred_horizontal.png", width = 13, height = 6.4, units = "in")
> #ggsave(file="plots/scatter/combined_pred_horizontal.pdf")
> ##############################################################################
>
> ####### 5) Additional exploratory analyses
>
> ##### a) Robustness check: Full model
>
> # 1) Full model with survey wave random effects
> m_full = glmer(donate_blood ~ age_std + gender + education_std + partner_status + employment_status +
parental_status + type_of_community + mean_confidence_healthcare_std + haq_index_std + health_exp_per_GDP_std +
(1|country) + (1|year_EVS) + (1|year_haq) + (1|year_health_exp), data = data_full, family = "binomial")
> summary(m_full)
Generalized linear mixed model fit by maximum likelihood (Laplace Approximation) ['glmerMod']
 Family: binomial  ( logit )
Formula: donate_blood ~ age_std + gender + education_std + partner_status +      employment_status +
parental_status + type_of_community +
    mean_confidence_healthcare_std + haq_index_std + health_exp_per_GDP_std +      (1 | country) + (1 | year_EVS)
+ (1 | year_haq) + (1 | year_health_exp)
   Data: data_full

     AIC      BIC   logLik deviance df.resid
 33415.0  33545.7 -16691.5  33383.0    26003

Scaled residuals:
    Min      1Q  Median      3Q     Max
-2.4912 -0.7907 -0.6027  1.0879  3.0750

Random effects:
```

```
 Groups          Name        Variance Std.Dev.
 country         (Intercept) 0.11038  0.33223 
 year_health_exp (Intercept) 0.00199  0.04461 
 year_haq        (Intercept) 0.16741  0.40916 
 year_EVS        (Intercept) 0.01371  0.11707 
Number of obs: 26019, groups:  country, 28; year_health_exp, 20; year_haq, 6; year_EVS, 2

Fixed effects:
                              Estimate Std. Error z value Pr(>|z|)    
(Intercept)                   -0.61067    0.20478  -2.982 0.002863 ** 
age_std                       -0.10883    0.05982  -1.819 0.068856 .  
gender1                       -0.46365    0.02675 -17.335  < 2e-16 ***
education_std                  0.17224    0.01466  11.749  < 2e-16 ***
partner_status1                0.06155    0.02980   2.065 0.038900 *  
employment_status1             0.21911    0.03399   6.447 1.14e-10 ***
parental_status1               0.08437    0.04053   2.082 0.037374 *  
type_of_community1            -0.02860    0.03304  -0.866 0.386708    
type_of_community2            -0.06571    0.03547  -1.853 0.063944 .  
mean_confidence_healthcare_std  0.11989    0.03502   3.423 0.000619 ***
haq_index_std                  0.08345    0.05848   1.427 0.153603    
health_exp_per_GDP_std        -0.05123    0.04235  -1.210 0.226406    
---
Signif. codes:  0 '***' 0.001 '**' 0.01 '*' 0.05 '.' 0.1 ' ' 1

Correlation of Fixed Effects:
            (Intr) ag_std gendr1 edctn_ prtn_1 empl_1 prnt_1 typ__1 typ__2 mn_c__ hq_nd_
age_std      0.152                                                                      
gender1     -0.079  0.021                                                               
educatn_std  0.006  0.074 -0.015                                                        
prtnr_stts1 -0.054  0.141  0.113 -0.010                                                 
emplymnt_s1 -0.075  0.096  0.091 -0.042 -0.047                                          
prntl_stts1 -0.032 -0.045 -0.072  0.006 -0.200  0.017                                   
typ_f_cmmn1 -0.094 -0.002  0.009  0.060 -0.027  0.008 -0.018                            
typ_f_cmmn2 -0.083  0.017  0.009  0.108 -0.040  0.013 -0.033  0.564                     
mn_cnfdnc__  0.008  0.028 -0.002 -0.039  0.028  0.011  0.000 -0.009 -0.015              
haq_ndx_std -0.097 -0.072 -0.005 -0.031 -0.016 -0.019  0.000  0.002 -0.009 -0.049       
hlth___GDP_ -0.014 -0.026 -0.004  0.002  0.020 -0.010  0.030 -0.018  0.000 -0.231 -0.190
> print(summary(m_full),digits=2)
Generalized linear mixed model fit by maximum likelihood (Laplace Approximation) ['glmerMod']
 Family: binomial  ( logit )
Formula: donate_blood ~ age_std + gender + education_std + partner_status +      employment_status + 
parental_status + type_of_community +  
    mean_confidence_healthcare_std + haq_index_std + health_exp_per_GDP_std +      (1 | country) + (1 | year_EVS) 
+ (1 | year_haq) + (1 | year_health_exp)
   Data: data_full

     AIC      BIC   logLik deviance df.resid 
 33415.0  33545.7 -16691.5  33383.0    26003 

Scaled residuals: 
   Min     1Q Median     3Q    Max 
 -2.49  -0.79  -0.60   1.09   3.08 

Random effects:
 Groups          Name        Variance Std.Dev.
 country         (Intercept) 0.110    0.332   
 year_health_exp (Intercept) 0.002    0.045   
 year_haq        (Intercept) 0.167    0.409   
 year_EVS        (Intercept) 0.014    0.117   
Number of obs: 26019, groups:  country, 28; year_health_exp, 20; year_haq, 6; year_EVS, 2

Fixed effects:
                              Estimate Std. Error z value Pr(>|z|)    
(Intercept)                     -0.611      0.205    -3.0    0.003 ** 
age_std                         -0.109      0.060    -1.8    0.069 .  
gender1                         -0.464      0.027   -17.3   <2e-16 ***
education_std                    0.172      0.015    11.7   <2e-16 ***
partner_status1                  0.062      0.030     2.1    0.039 *  
employment_status1               0.219      0.034     6.4    1e-10 ***
parental_status1                 0.084      0.041     2.1    0.037 *  
type_of_community1              -0.029      0.033    -0.9    0.387    
type_of_community2              -0.066      0.035    -1.9    0.064 .  
mean_confidence_healthcare_std   0.120      0.035     3.4    6e-04 ***
haq_index_std                    0.083      0.058     1.4    0.154    
health_exp_per_GDP_std          -0.051      0.042    -1.2    0.226    
---
Signif. codes:  0 '***' 0.001 '**' 0.01 '*' 0.05 '.' 0.1 ' ' 1
```

```
Correlation of Fixed Effects:
            (Intr) ag_std gendr1 edctn_ prtn_1 empl_1 prnt_1 typ__1 typ__2 mn_c__ hq_nd_
age_std      0.152
gender1     -0.079  0.021
educatn_std  0.006  0.074 -0.015
prtnr_stts1 -0.054  0.141  0.113 -0.010
emplymnt_s1 -0.075  0.096  0.091 -0.042 -0.047
prntl_stts1 -0.032 -0.045 -0.072  0.006 -0.200  0.017
typ_f_cmmn1 -0.094 -0.002  0.009  0.060 -0.027  0.008 -0.018
typ_f_cmmn2 -0.083  0.017  0.009  0.108 -0.040  0.013 -0.033  0.564
mn_cnfdnc__  0.008  0.028 -0.002 -0.039  0.028  0.011  0.000 -0.009 -0.015
haq_ndx_std -0.097 -0.072 -0.005 -0.031 -0.016 -0.019  0.000  0.002 -0.009 -0.049
hlth___GDP_ -0.014 -0.026 -0.004  0.002  0.020 -0.010  0.030 -0.018  0.000 -0.231 -0.190
> # significant effects: mean_confidence_healthcare (higher trust MORE)
> # HAQ and exp not significant
>
> # 2) Full model without survey wave random effects
> m_full_no_RE_wave = glmer(donate_blood ~ age_std + gender + education_std + partner_status + employment_status +
parental_status + type_of_community + mean_confidence_healthcare_std + haq_index_std + health_exp_per_GDP_std +
(1|country), data = data_full, family = "binomial")
> summary(m_full_no_RE_wave)
Generalized linear mixed model fit by maximum likelihood (Laplace Approximation) ['glmerMod']
 Family: binomial  ( logit )
Formula: donate_blood ~ age_std + gender + education_std + partner_status +      employment_status +
parental_status + type_of_community +
    mean_confidence_healthcare_std + haq_index_std + health_exp_per_GDP_std +      (1 | country)
   Data: data_full

     AIC      BIC   logLik deviance df.resid
 33448.3  33554.5 -16711.2  33422.3    26006

Scaled residuals:
    Min      1Q  Median      3Q     Max
-2.6815 -0.7889 -0.6059  1.0938  3.3108

Random effects:
 Groups  Name        Variance Std.Dev.
 country (Intercept) 0.1382   0.3718
Number of obs: 26019, groups:  country, 28

Fixed effects:
                               Estimate Std. Error z value Pr(>|z|)
(Intercept)                    -0.43838    0.07970  -5.500 3.79e-08 ***
age_std                         0.09704    0.03257   2.980  0.00289 **
gender1                        -0.45245    0.02662 -16.996  < 2e-16 ***
education_std                   0.17882    0.01464  12.211  < 2e-16 ***
partner_status1                 0.09532    0.02924   3.260  0.00111 **
employment_status1              0.27990    0.03020   9.269  < 2e-16 ***
parental_status1                0.05467    0.03766   1.452  0.14662
type_of_community1             -0.02420    0.03300  -0.733  0.46331
type_of_community2             -0.05894    0.03541  -1.664  0.09604 .
mean_confidence_healthcare_std  0.16548    0.03440   4.810 1.51e-06 ***
haq_index_std                  -0.11352    0.04843  -2.344  0.01906 *
health_exp_per_GDP_std         -0.12405    0.04118  -3.012  0.00259 **
---
Signif. codes:  0 ‘***’ 0.001 ‘**’ 0.01 ‘*’ 0.05 ‘.’ 0.1 ‘ ’ 1

Correlation of Fixed Effects:
            (Intr) ag_std gendr1 edctn_ prtn_1 empl_1 prnt_1 typ__1 typ__2 mn_c__ hq_nd_
age_std     -0.055
gender1     -0.210  0.008
educatn_std -0.003  0.076 -0.021
prtnr_stts1 -0.204  0.055  0.102 -0.025
emplymnt_s1 -0.164  0.270  0.062 -0.072 -0.119
prntl_stts1 -0.012  0.088 -0.081 -0.004 -0.221 -0.034
typ_f_cmmn1 -0.243 -0.026  0.007  0.059 -0.033 -0.001 -0.019
typ_f_cmmn2 -0.223 -0.012  0.007  0.107 -0.047  0.007 -0.031  0.563
mn_cnfdnc__ -0.024 -0.152 -0.012 -0.046 -0.002 -0.042  0.047 -0.015 -0.021
haq_ndx_std -0.029  0.732  0.016 -0.008  0.083  0.019 -0.073 -0.003  0.001 -0.008
hlth___GDP_  0.000  0.290  0.020  0.013  0.090  0.132 -0.037 -0.015  0.010 -0.176 -0.217
> print(summary(m_full_no_RE_wave),digits=2)
Generalized linear mixed model fit by maximum likelihood (Laplace Approximation) ['glmerMod']
 Family: binomial  ( logit )
Formula: donate_blood ~ age_std + gender + education_std + partner_status +      employment_status +
parental_status + type_of_community +
    mean_confidence_healthcare_std + haq_index_std + health_exp_per_GDP_std +      (1 | country)
```

```
   Data: data_full

     AIC      BIC   logLik deviance df.resid
 33448.3  33554.5 -16711.2  33422.3    26006

Scaled residuals:
   Min     1Q Median     3Q    Max
 -2.68  -0.79  -0.61   1.09   3.31

Random effects:
 Groups  Name        Variance Std.Dev.
 country (Intercept) 0.14     0.37
Number of obs: 26019, groups:  country, 28

Fixed effects:
                              Estimate Std. Error z value Pr(>|z|)
(Intercept)                     -0.438      0.080    -5.5    4e-08 ***
age_std                          0.097      0.033     3.0    0.003 **
gender1                         -0.452      0.027   -17.0   <2e-16 ***
education_std                    0.179      0.015    12.2   <2e-16 ***
partner_status1                  0.095      0.029     3.3    0.001 **
employment_status1               0.280      0.030     9.3   <2e-16 ***
parental_status1                 0.055      0.038     1.5    0.147
type_of_community1              -0.024      0.033    -0.7    0.463
type_of_community2              -0.059      0.035    -1.7    0.096 .
mean_confidence_healthcare_std   0.165      0.034     4.8    2e-06 ***
haq_index_std                   -0.114      0.048    -2.3    0.019 *
health_exp_per_GDP_std          -0.124      0.041    -3.0    0.003 **
---
Signif. codes:  0 '***' 0.001 '**' 0.01 '*' 0.05 '.' 0.1 ' ' 1

Correlation of Fixed Effects:
            (Intr) ag_std gendr1 edctn_ prtn_1 empl_1 prnt_1 typ__1 typ__2 mn_c__ hq_nd_
age_std     -0.055
gender1     -0.210  0.008
educatn_std -0.003  0.076 -0.021
prtnr_stts1 -0.204  0.055  0.102 -0.025
emplymnt_s1 -0.164  0.270  0.062 -0.072 -0.119
prntl_stts1 -0.012  0.088 -0.081 -0.004 -0.221 -0.034
typ_f_cmmn1 -0.243 -0.026  0.007  0.059 -0.033 -0.001 -0.019
typ_f_cmmn2 -0.223 -0.012  0.007  0.107 -0.047  0.007 -0.031  0.563
mn_cnfdnc__ -0.024 -0.152 -0.012 -0.046 -0.002 -0.042  0.047 -0.015 -0.021
haq_ndx_std -0.029  0.732  0.016 -0.008  0.083  0.019 -0.073 -0.003  0.001 -0.008
hlth___GDP_  0.000  0.290  0.020  0.013  0.090  0.132 -0.037 -0.015  0.010 -0.176 -0.217
>
>
> ##### b) Robustness check: Subset of young respondents
>
> data_young = filter(data_full, age < 44)
> # With age < 58, all group n > 250
> # With age < 44, all group n > 250 (EXCEPT small samples: Malta, Cyprus, Luxembourg)
>
> data_young_resp_per_country = data_young%>%
+   group_by(country)%>%
+   dplyr::summarise(num_resp = n())
>
> # Descriptives
> describe(data_young$donate_blood) # mean: 0.34
data_young$donate_blood
       n  missing distinct
    9640        0        2

Value          0     1
Frequency   6347  3293
Proportion 0.658 0.342
> describe(data_young$gender) # mean: 0.56
data_young$gender
       n  missing distinct
    9640        0        2

Value          0     1
Frequency   4280  5360
Proportion 0.444 0.556
> psych::describe(data_young$age) # mean 31.82; median 32; range 18 - 43
   vars    n  mean   sd median trimmed mad min max range  skew kurtosis   se
X1    1 9640 31.82 7.37     32   32.04 8.9  18  43    25 -0.21    -1.11 0.08
> psych::describe(data_young$education) # mean 20.26; median 19; range 0 - 43; NA: 188
```

```
   vars    n  mean   sd median trimmed  mad min max range skew kurtosis   se
X1    1 9452 20.26 4.14     19   20.03 2.97   0  43    43 0.42     4.77 0.04
> describe(data_young$partner_status) # mean: 0.65
data_young$partner_status
       n  missing distinct 
    9609       31        2 

Value          0     1
Frequency   3382  6227
Proportion 0.352 0.648
> describe(data_young$employment_status) # mean: 0.68
data_young$employment_status
       n  missing distinct 
    9640        0        2 

Value          0     1
Frequency   3124  6516
Proportion 0.324 0.676
> describe(data_young$parental_status) # mean: 0.42
data_young$parental_status
       n  missing distinct 
    9638        2        2 

Value          0     1
Frequency   5547  4091
Proportion 0.576 0.424
> describe(data_young$type_of_community) # 0: 2899 (0.30); 1: 4030 (0.42); 2: 2706 (0.28)
data_young$type_of_community
       n  missing distinct 
    9635        5        3 

Value          0     1     2
Frequency   2899  4030  2706
Proportion 0.301 0.418 0.281
> 
> # Summary statistics (country-level)
> psych::describe(data_young$mean_confidence_healthcare)
   vars    n mean   sd median trimmed  mad  min  max range skew kurtosis se
X1    1 9640 2.57 0.33   2.53    2.56 0.39 1.84 3.23  1.38 0.09    -0.71  0
> psych::describe(data_young$haq_index)
   vars    n  mean   sd median trimmed  mad  min  max range  skew kurtosis   se
X1    1 9640 79.91 6.07   81.8   80.37 5.78 64.2 90.5  26.3 -0.63    -0.59 0.06
> psych::describe(data_young$health_exp_per_GDP)
   vars    n mean   sd median trimmed  mad  min   max range skew kurtosis   se
X1    1 9640 8.19 1.62    8.2    8.18 1.92 4.57 11.97  7.39 0.07    -0.82 0.02
> 
> 
> ## 1) Mixed-effects models
> 
> # a) Trust
> m4 = glmer(donate_blood ~ age_std + gender + education_std + partner_status + employment_status +
parental_status + type_of_community + mean_confidence_healthcare_std + (1|country) + (1|year_EVS), data =
data_young, family = "binomial")
> summary(m4)
Generalized linear mixed model fit by maximum likelihood (Laplace Approximation) ['glmerMod']
 Family: binomial  ( logit )
Formula: donate_blood ~ age_std + gender + education_std + partner_status +      employment_status +
parental_status + type_of_community +  
    mean_confidence_healthcare_std + (1 | country) + (1 | year_EVS)
   Data: data_young

     AIC      BIC   logLik deviance df.resid 
 11725.7  11811.5  -5850.9  11701.7     9409 

Scaled residuals: 
    Min      1Q  Median      3Q     Max 
-1.6545 -0.7247 -0.5859  1.1520  3.7966 

Random effects:
 Groups   Name        Variance  Std.Dev.
 country  (Intercept) 0.0988255 0.31437 
 year_EVS (Intercept) 0.0007368 0.02714 
Number of obs: 9421, groups:  country, 28; year_EVS, 2

Fixed effects:
                                Estimate Std. Error z value Pr(>|z|)   
(Intercept)                    -0.411448   0.155742  -2.642  0.00825 **
```

```
age_std                         0.360093   0.116293   3.096  0.00196 **
gender1                        -0.304347   0.045658  -6.666 2.63e-11 ***
education_std                   0.287358   0.032327   8.889  < 2e-16 ***
partner_status1                 0.066850   0.054694   1.222  0.22161
employment_status1              0.268491   0.055328   4.853 1.22e-06 ***
parental_status1                0.067133   0.052730   1.273  0.20296
type_of_community1              0.007326   0.054982   0.133  0.89400
type_of_community2             -0.044771   0.060449  -0.741  0.45890
mean_confidence_healthcare_std  0.025392   0.047129   0.539  0.59004
---
Signif. codes:  0 ‘***’ 0.001 ‘**’ 0.01 ‘*’ 0.05 ‘.’ 0.1 ‘ ’ 1

Correlation of Fixed Effects:
            (Intr) ag_std gendr1 edctn_ prtn_1 empl_1 prnt_1 typ__1 typ__2
age_std      0.792
gender1     -0.184 -0.028
educatn_std -0.155 -0.130 -0.055
prtnr_stts1 -0.307 -0.219 -0.020  0.014
emplymnt_s1 -0.504 -0.366  0.123  0.030 -0.083
prntl_stts1 -0.270 -0.326 -0.101  0.056 -0.283  0.113
typ_f_cmmn1 -0.214 -0.020 -0.012  0.061 -0.013  0.006 -0.034
typ_f_cmmn2 -0.183 -0.011 -0.018  0.108 -0.041 -0.003 -0.049  0.535
mn_cnfdnc__  0.075  0.114  0.007 -0.062  0.011 -0.040 -0.051 -0.036 -0.041
> print(summary(m4),digits=1)
Generalized linear mixed model fit by maximum likelihood (Laplace Approximation) ['glmerMod']
 Family: binomial  ( logit )
Formula: donate_blood ~ age_std + gender + education_std + partner_status +      employment_status +
parental_status + type_of_community +
    mean_confidence_healthcare_std + (1 | country) + (1 | year_EVS)
   Data: data_young

     AIC      BIC   logLik deviance df.resid
 11725.7  11811.5  -5850.9  11701.7     9409

Scaled residuals:
   Min     1Q Median     3Q    Max
  -1.7   -0.7   -0.6    1.2    3.8

Random effects:
 Groups   Name        Variance Std.Dev.
 country  (Intercept) 1e-01    0.31
 year_EVS (Intercept) 7e-04    0.03
Number of obs: 9421, groups:  country, 28; year_EVS, 2

Fixed effects:
                               Estimate Std. Error z value Pr(>|z|)
(Intercept)                      -0.411      0.156    -2.6    0.008 **
age_std                           0.360      0.116     3.1    0.002 **
gender1                          -0.304      0.046    -6.7    3e-11 ***
education_std                     0.287      0.032     8.9   <2e-16 ***
partner_status1                   0.067      0.055     1.2    0.222
employment_status1                0.268      0.055     4.9    1e-06 ***
parental_status1                  0.067      0.053     1.3    0.203
type_of_community1                0.007      0.055     0.1    0.894
type_of_community2               -0.045      0.060    -0.7    0.459
mean_confidence_healthcare_std    0.025      0.047     0.5    0.590
---
Signif. codes:  0 ‘***’ 0.001 ‘**’ 0.01 ‘*’ 0.05 ‘.’ 0.1 ‘ ’ 1

Correlation of Fixed Effects:
            (Intr) ag_std gendr1 edctn_ prtn_1 empl_1 prnt_1 typ__1 typ__2
age_std      0.792
gender1     -0.184 -0.028
educatn_std -0.155 -0.130 -0.055
prtnr_stts1 -0.307 -0.219 -0.020  0.014
emplymnt_s1 -0.504 -0.366  0.123  0.030 -0.083
prntl_stts1 -0.270 -0.326 -0.101  0.056 -0.283  0.113
typ_f_cmmn1 -0.214 -0.020 -0.012  0.061 -0.013  0.006 -0.034
typ_f_cmmn2 -0.183 -0.011 -0.018  0.108 -0.041 -0.003 -0.049  0.535
mn_cnfdnc__  0.075  0.114  0.007 -0.062  0.011 -0.040 -0.051 -0.036 -0.041
> # Not significant
>
> # b) Healthcare quality
> m5 = glmer(donate_blood ~ age_std + gender + education_std + partner_status + employment_status +
parental_status + type_of_community + haq_index_std + health_exp_per_GDP_std + (1|country) + (1|year_haq) +
(1|year_health_exp), data = data_young, family = "binomial")
> summary(m5)
```

```
Generalized linear mixed model fit by maximum likelihood (Laplace Approximation) ['glmerMod']
 Family: binomial  ( logit )
Formula: donate_blood ~ age_std + gender + education_std + partner_status +      employment_status +
parental_status + type_of_community +
    haq_index_std + health_exp_per_GDP_std + (1 | country) +      (1 | year_haq) + (1 | year_health_exp)
   Data: data_young

     AIC      BIC   logLik deviance df.resid
 11722.8  11822.9  -5847.4  11694.8     9407

Scaled residuals:
    Min      1Q  Median      3Q     Max
-1.6914 -0.7255 -0.5855  1.1497  3.7277

Random effects:
 Groups          Name        Variance  Std.Dev.
 country         (Intercept) 0.0978127 0.31275
 year_health_exp (Intercept) 0.0007704 0.02776
 year_haq        (Intercept) 0.0059181 0.07693
Number of obs: 9421, groups:  country, 28; year_health_exp, 13; year_haq, 4

Fixed effects:
                        Estimate Std. Error z value Pr(>|z|)
(Intercept)            -0.341072   0.159012  -2.145    0.032 *
age_std                 0.488064   0.107175   4.554 5.27e-06 ***
gender1                -0.308081   0.045748  -6.734 1.65e-11 ***
education_std           0.281493   0.032238   8.732  < 2e-16 ***
partner_status1         0.062571   0.054524   1.148    0.251
employment_status1      0.255944   0.054711   4.678 2.89e-06 ***
parental_status1        0.080574   0.051122   1.576    0.115
type_of_community1      0.005803   0.055004   0.106    0.916
type_of_community2     -0.044958   0.060457  -0.744    0.457
haq_index_std           0.061881   0.090076   0.687    0.492
health_exp_per_GDP_std  0.048255   0.068286   0.707    0.480
---
Signif. codes:  0 '***' 0.001 '**' 0.01 '*' 0.05 '.' 0.1 ' ' 1

Correlation of Fixed Effects:
            (Intr) ag_std gendr1 edctn_ prtn_1 empl_1 prnt_1 typ__1 typ__2 hq_nd_
age_std      0.717
gender1     -0.186 -0.037
educatn_std -0.090 -0.055 -0.051
prtnr_stts1 -0.246 -0.135 -0.018  0.009
emplymnt_s1 -0.398 -0.223  0.131  0.018 -0.095
prntl_stts1 -0.094 -0.059 -0.109  0.019 -0.322  0.056
typ_f_cmmn1 -0.205 -0.025 -0.011  0.062 -0.013  0.003 -0.040
typ_f_cmmn2 -0.176 -0.019 -0.018  0.107 -0.041 -0.005 -0.053  0.535
haq_ndx_std -0.017  0.349  0.006 -0.053  0.012  0.015  0.006 -0.027 -0.029
hlth___GDP_  0.027 -0.031 -0.002  0.039  0.006 -0.019  0.004  0.009  0.013 -0.601
> print(summary(m5),digits=1)
Generalized linear mixed model fit by maximum likelihood (Laplace Approximation) ['glmerMod']
 Family: binomial  ( logit )
Formula: donate_blood ~ age_std + gender + education_std + partner_status +      employment_status +
parental_status + type_of_community +
    haq_index_std + health_exp_per_GDP_std + (1 | country) +      (1 | year_haq) + (1 | year_health_exp)
   Data: data_young

     AIC      BIC   logLik deviance df.resid
 11722.8  11822.9  -5847.4  11694.8     9407

Scaled residuals:
   Min     1Q Median     3Q    Max
  -1.7   -0.7   -0.6    1.1    3.7

Random effects:
 Groups          Name        Variance Std.Dev.
 country         (Intercept) 1e-01    0.31
 year_health_exp (Intercept) 8e-04    0.03
 year_haq        (Intercept) 6e-03    0.08
Number of obs: 9421, groups:  country, 28; year_health_exp, 13; year_haq, 4

Fixed effects:
                       Estimate Std. Error z value Pr(>|z|)
(Intercept)              -0.341      0.159    -2.1     0.03 *
age_std                   0.488      0.107     4.6    5e-06 ***
gender1                  -0.308      0.046    -6.7    2e-11 ***
education_std             0.281      0.032     8.7   <2e-16 ***
```

```
partner_status1           0.063      0.055     1.1     0.25
employment_status1        0.256      0.055     4.7    3e-06 ***
parental_status1          0.081      0.051     1.6     0.11
type_of_community1        0.006      0.055     0.1     0.92
type_of_community2       -0.045      0.060    -0.7     0.46
haq_index_std             0.062      0.090     0.7     0.49
health_exp_per_GDP_std    0.048      0.068     0.7     0.48
---
Signif. codes:  0 ‘***’ 0.001 ‘**’ 0.01 ‘*’ 0.05 ‘.’ 0.1 ‘ ’ 1

Correlation of Fixed Effects:
            (Intr) ag_std gendr1 edctn_ prtn_1 empl_1 prnt_1 typ__1 typ__2 hq_nd_
age_std      0.717
gender1     -0.186 -0.037
educatn_std -0.090 -0.055 -0.051
prtnr_stts1 -0.246 -0.135 -0.018  0.009
emplymnt_s1 -0.398 -0.223  0.131  0.018 -0.095
prntl_stts1 -0.094 -0.059 -0.109  0.019 -0.322  0.056
typ_f_cmmn1 -0.205 -0.025 -0.011  0.062 -0.013  0.003 -0.040
typ_f_cmmn2 -0.176 -0.019 -0.018  0.107 -0.041 -0.005 -0.053  0.535
haq_ndx_std -0.017  0.349  0.006 -0.053  0.012  0.015  0.006 -0.027 -0.029
hlth___GDP_  0.027 -0.031 -0.002  0.039  0.006 -0.019  0.004  0.009  0.013 -0.601
> # Both not significant
>
> # m_full_young does not converge
> # m_full_young = glmer(donate_blood ~ age_std + gender + education_std + partner_status + employment_status +
parental_status + type_of_community + mean_confidence_healthcare_std + haq_index_std + health_exp_per_GDP_std +
(1|country) + (1|year_EVS) + (1|year_haq)  + (1|year_health_exp), data = data_young, family = "binomial")
>
>
> # 2) Map plots
> mean_indicators_young = data_young%>%
+   group_by(iso_code_alpha2)%>%
+   dplyr::summarize(mean_confidence_healthcare = mean(mean_confidence_healthcare),
+                    haq_index = mean(haq_index),
+                    health_exp_per_GDP = mean(health_exp_per_GDP))%>%
+   rename(iso_a2 = iso_code_alpha2)
>
> # Add to map data
> map_data_young = left_join(europe.clipped, mean_indicators_young, by="iso_a2")
>
> trust_young = ggplot(data=subset(map_data_young, sovereignt != "Belarus" & sovereignt != "Ukraine" & sovereignt
!= "Moldova"), aes(fill=mean_confidence_healthcare)) +
+   geom_sf(alpha=0.8,col='white') +
+   coord_sf(crs="+proj=aea +lat_1=36.333333333333336 +lat_2=65.66666666666667 +lon_0=14") +
+   viridis::scale_fill_viridis(name='', direction = 1, na.value = "grey92") +
+   labs(x=NULL, y=NULL, title="Trust in the healthcare system")+
+   theme(legend.position="bottom", legend.direction = "horizontal", legend.key.width = unit(1.3,"cm"), plot.title
= element_text(hjust = 0.5))
>
> haq_young = ggplot(data=subset(map_data_young, sovereignt != "Belarus" & sovereignt != "Ukraine" & sovereignt !=
"Moldova"), aes(fill=haq_index)) +
+   geom_sf(alpha=0.8,col='white') +
+   coord_sf(crs="+proj=aea +lat_1=36.333333333333336 +lat_2=65.66666666666667 +lon_0=14") +
+   viridis::scale_fill_viridis(name='', direction = 1, na.value = "grey92") +
+   labs(x=NULL, y=NULL, title="HAQ index")+
+   theme(legend.position="bottom", legend.direction = "horizontal", legend.key.width = unit(1.3,"cm"), plot.title
= element_text(hjust = 0.5))
>
> health_exp_young = ggplot(data=subset(map_data_young, sovereignt != "Belarus" & sovereignt != "Ukraine" &
sovereignt != "Moldova"), aes(fill=health_exp_per_GDP)) +
+   geom_sf(alpha=0.8,col='white') +
+   coord_sf(crs="+proj=aea +lat_1=36.333333333333336 +lat_2=65.66666666666667 +lon_0=14") +
+   viridis::scale_fill_viridis(name='', direction = 1, na.value = "grey92") +
+   labs(x=NULL, y=NULL, title="Health expenditures per GDP")+
+   theme(legend.position="bottom", legend.direction = "horizontal", legend.key.width = unit(1.3,"cm"), plot.title
= element_text(hjust = 0.5))
>
> ggarrange(trust_young, haq_young, health_exp_young,
+           labels = c("A", "B", "C"),
+           ncol = 3, nrow = 1)
>
> #ggsave("plots/maps/combined_indicators_young.png", width = 14, height = 6.97, units = "in")
>
>
> ##### c) Robustness check: Analyses excluding each of the countries
>
```

```
> excluding_Austria = filter(data_full, country != "Austria")
> excluding_Belgium = filter(data_full, country != "Belgium")
> excluding_Bulgaria = filter(data_full, country != "Bulgaria")
> excluding_Croatia = filter(data_full, country != "Croatia")
> excluding_Cyprus = filter(data_full, country != "Cyprus")
> excluding_Czech_Republic = filter(data_full, country != "Czech Republic")
> excluding_Denmark = filter(data_full, country != "Denmark")
> excluding_Estonia = filter(data_full, country != "Estonia")
> excluding_Finland = filter(data_full, country != "Finland")
> excluding_France = filter(data_full, country != "France")
> excluding_Germany = filter(data_full, country != "Germany")
> excluding_Greece = filter(data_full, country != "Greece")
> excluding_Hungary = filter(data_full, country != "Hungary")
> excluding_Ireland = filter(data_full, country != "Ireland")
> excluding_Italy = filter(data_full, country != "Italy")
> excluding_Latvia = filter(data_full, country != "Latvia")
> excluding_Lithuania = filter(data_full, country != "Lithuania")
> excluding_Luxembourg = filter(data_full, country != "Luxembourg")
> excluding_Malta = filter(data_full, country != "Malta")
> excluding_Netherlands = filter(data_full, country != "Netherlands")
> excluding_Poland = filter(data_full, country != "Poland")
> excluding_Portugal = filter(data_full, country != "Portugal")
> excluding_Romania = filter(data_full, country != "Romania")
> excluding_Slovakia = filter(data_full, country != "Slovakia")
> excluding_Slovenia = filter(data_full, country != "Slovenia")
> excluding_Spain = filter(data_full, country != "Spain")
> excluding_Sweden = filter(data_full, country != "Sweden")
> excluding_United_Kingdom = filter(data_full, country != "United Kingdom")
>
> # Trust in the healthcare system
> summary(glmer(data = excluding_United_Kingdom, donate_blood ~ age_std + gender + education_std + partner_status
+ employment_status + parental_status + type_of_community + mean_confidence_healthcare_std + (1|country) +
(1|year_EVS), family = "binomial"))
Generalized linear mixed model fit by maximum likelihood (Laplace Approximation) ['glmerMod']
 Family: binomial  ( logit )
Formula: donate_blood ~ age_std + gender + education_std + partner_status +      employment_status +
parental_status + type_of_community +
    mean_confidence_healthcare_std + (1 | country) + (1 | year_EVS)
   Data: excluding_United_Kingdom

     AIC      BIC   logLik deviance df.resid
 31742.2  31839.7 -15859.1  31718.2    24759

Scaled residuals:
    Min      1Q  Median      3Q     Max
-2.6513 -0.7849 -0.5991  1.0880  3.3149

Random effects:
 Groups   Name        Variance Std.Dev.
 country  (Intercept) 0.10890  0.3300
 year_EVS (Intercept) 0.02339  0.1529
Number of obs: 24771, groups:  country, 27; year_EVS, 2

Fixed effects:
                               Estimate Std. Error z value Pr(>|z|)
(Intercept)                    -0.44095    0.13131  -3.358 0.000785 ***
age_std                         0.09409    0.02529   3.720 0.000199 ***
gender1                        -0.48606    0.02736 -17.769  < 2e-16 ***
education_std                   0.17612    0.01498  11.757  < 2e-16 ***
partner_status1                 0.08516    0.02996   2.843 0.004469 **
employment_status1              0.25061    0.03163   7.924 2.30e-15 ***
parental_status1                0.08232    0.03926   2.097 0.036031 *
type_of_community1             -0.04751    0.03399  -1.398 0.162178
type_of_community2             -0.08383    0.03644  -2.301 0.021398 *
mean_confidence_healthcare_std  0.15516    0.03720   4.171 3.04e-05 ***
---
Signif. codes:  0 ‘***’ 0.001 ‘**’ 0.01 ‘*’ 0.05 ‘.’ 0.1 ‘ ’ 1

Correlation of Fixed Effects:
            (Intr) ag_std gendr1 edctn_ prtn_1 empl_1 prnt_1 typ__1 typ__2
age_std      0.004
gender1     -0.129  0.015
educatn_std -0.003  0.107 -0.023
prtnr_stts1 -0.121  0.013  0.102 -0.023
emplymnt_s1 -0.089  0.430  0.068 -0.072 -0.103
prntl_stts1 -0.016  0.070 -0.083 -0.006 -0.229 -0.073
typ_f_cmmn1 -0.153 -0.013  0.010  0.056 -0.029  0.003 -0.020
```

```
typ_f_cmmn2 -0.139 -0.003  0.011  0.109 -0.047  0.011 -0.035  0.566
mn_cnfdnc__ -0.002 -0.005 -0.004 -0.051  0.029  0.011  0.018 -0.014 -0.027
>
> # Healthcare quality
> summary(glmer(data = excluding_United_Kingdom, donate_blood ~ age_std + gender + education_std + partner_status
+ employment_status + parental_status + type_of_community + haq_index_std + health_exp_per_GDP_std + (1|country) +
(1|year_haq) + (1|year_health_exp), family = "binomial"))
Generalized linear mixed model fit by maximum likelihood (Laplace Approximation) ['glmerMod']
 Family: binomial  ( logit )
Formula: donate_blood ~ age_std + gender + education_std + partner_status +      employment_status +
parental_status + type_of_community +
    haq_index_std + health_exp_per_GDP_std + (1 | country) +      (1 | year_haq) + (1 | year_health_exp)
   Data: excluding_United_Kingdom

     AIC      BIC   logLik deviance df.resid
 31737.7  31851.3 -15854.8  31709.7    24757

Scaled residuals:
    Min      1Q  Median      3Q     Max
-2.4895 -0.7897 -0.6002  1.0865  3.0788

Random effects:
 Groups          Name        Variance Std.Dev.
 country         (Intercept) 0.104130 0.32269
 year_health_exp (Intercept) 0.003308 0.05751
 year_haq        (Intercept) 0.287395 0.53609
Number of obs: 24771, groups:  country, 27; year_health_exp, 20; year_haq, 6

Fixed effects:
                        Estimate Std. Error z value Pr(>|z|)
(Intercept)            -0.608254   0.234905  -2.589  0.00962 **
age_std                -0.145487   0.060645  -2.399  0.01644 *
gender1                -0.492959   0.027451 -17.958  < 2e-16 ***
education_std           0.173060   0.014983  11.550  < 2e-16 ***
partner_status1         0.050213   0.030576   1.642  0.10054
employment_status1      0.223876   0.035075   6.383 1.74e-10 ***
parental_status1        0.080462   0.041704   1.929  0.05369 .
type_of_community1     -0.045752   0.034007  -1.345  0.17850
type_of_community2     -0.083260   0.036448  -2.284  0.02235 *
haq_index_std           0.098412   0.057731   1.705  0.08826 .
health_exp_per_GDP_std -0.009444   0.041770  -0.226  0.82113
---
Signif. codes:  0 ‘***’ 0.001 ‘**’ 0.01 ‘*’ 0.05 ‘.’ 0.1 ‘ ’ 1

Correlation of Fixed Effects:
            (Intr) ag_std gendr1 edctn_ prtn_1 empl_1 prnt_1 typ__1 typ__2 hq_nd_
age_std      0.130
gender1     -0.072  0.019
educatn_std  0.005  0.073 -0.018
prtnr_stts1 -0.052  0.132  0.112 -0.009
emplymnt_s1 -0.071  0.084  0.091 -0.043 -0.043
prntl_stts1 -0.027 -0.045 -0.068  0.006 -0.199  0.021
typ_f_cmmn1 -0.085 -0.003  0.011  0.056 -0.027  0.008 -0.018
typ_f_cmmn2 -0.075  0.016  0.012  0.108 -0.042  0.014 -0.034  0.565
haq_ndx_std -0.079 -0.053 -0.004 -0.034 -0.008 -0.013 -0.004  0.000 -0.008
hlth___GDP_ -0.008 -0.019 -0.003 -0.005  0.028 -0.003  0.039 -0.019 -0.005 -0.237
>
> # All results robust to exclusion of single countries EXCEPT HAQ index,
> # which in 6 specifications has a significant positive effect:
>
> # excluding_Italy: haq_index_std positive significant effect: b = 0.112 (0.056), p = 0.04396 *
> # excluding_Latvia: haq_index_std positive significant effect: b = 0.121 (0.059), p = 0.03975 *
> # excluding_Lithuania: haq_index_std positive significant effect: b = 0.135 (0.060), p = 0.02360 *
> # excluding_Malta: haq_index_std positive significant effect: b = 0.126 (0.058), p = 0.02813 *
> # excluding_Slovenia: haq_index_std positive significant effect: b = 0.116 (0.057), p = 0.04201 *
> # excluding_Sweden: haq_index_std positive significant effect: b = 0.126 (0.057), p = 0.02755 *
>
> ##### d) Robustness check: Mean of country-level IVs aggregated over time
>
> data_full$mean_confidence_healthcare_agg_no_time_std =
as.numeric(scale(data_full$mean_confidence_healthcare_agg_no_time))
> data_full$haq_agg_no_time_std = as.numeric(scale(data_full$haq_agg_no_time))
> data_full$health_exp_per_GDP_agg_no_time_std = as.numeric(scale(data_full$health_exp_per_GDP_agg_no_time))
>
> # a) Trust
> m6 = glmer(donate_blood ~ age_std + gender + education_std + partner_status + employment_status +
parental_status + type_of_community + mean_confidence_healthcare_agg_no_time_std + (1|country) + (1|year_EVS),
```

```
data = data_full, family = "binomial")
> summary(m6)
Generalized linear mixed model fit by maximum likelihood (Laplace Approximation) ['glmerMod']
 Family: binomial  ( logit )
Formula: donate_blood ~ age_std + gender + education_std + partner_status +      employment_status +
parental_status + type_of_community +
    mean_confidence_healthcare_agg_no_time_std + (1 | country) +      (1 | year_EVS)
   Data: data_full

     AIC      BIC   logLik deviance df.resid
 33442.9  33540.9 -16709.5  33418.9    26007

Scaled residuals:
    Min      1Q  Median      3Q     Max
-2.6116 -0.7904 -0.6071  1.0945  3.1775

Random effects:
 Groups   Name        Variance Std.Dev.
 country  (Intercept) 0.09675  0.3110
 year_EVS (Intercept) 0.03107  0.1763
Number of obs: 26019, groups:  country, 28; year_EVS, 2

Fixed effects:
                                           Estimate Std. Error z value Pr(>|z|)
(Intercept)                                -0.47628    0.14291  -3.333 0.000860 ***
age_std                                     0.09341    0.02446   3.819 0.000134 ***
gender1                                    -0.45641    0.02664 -17.135  < 2e-16 ***
education_std                               0.18018    0.01462  12.320  < 2e-16 ***
partner_status1                             0.08923    0.02912   3.064 0.002184 **
employment_status1                          0.24967    0.03076   8.117 4.79e-16 ***
parental_status1                            0.08562    0.03824   2.239 0.025144 *
type_of_community1                         -0.02547    0.03299  -0.772 0.440159
type_of_community2                         -0.05938    0.03541  -1.677 0.093572 .
mean_confidence_healthcare_agg_no_time_std  0.03556    0.05868   0.606 0.544517
---
Signif. codes:  0 ‘***’ 0.001 ‘**’ 0.01 ‘*’ 0.05 ‘.’ 0.1 ‘ ’ 1

Correlation of Fixed Effects:
            (Intr) ag_std gendr1 edctn_ prtn_1 empl_1 prnt_1 typ__1 typ__2
age_std      0.004
gender1     -0.115  0.017
educatn_std -0.001  0.109 -0.020
prtnr_stts1 -0.108  0.010  0.104 -0.023
emplymnt_s1 -0.080  0.428  0.068 -0.070 -0.107
prntl_stts1 -0.015  0.074 -0.086 -0.006 -0.228 -0.070
typ_f_cmmn1 -0.135 -0.017  0.007  0.059 -0.030  0.003 -0.020
typ_f_cmmn2 -0.123 -0.008  0.008  0.107 -0.046  0.010 -0.033  0.564
mn_cnf_____ -0.024 -0.013  0.002 -0.025  0.003  0.004 -0.003 -0.020 -0.029
> print(summary(m6),digits=2)
Generalized linear mixed model fit by maximum likelihood (Laplace Approximation) ['glmerMod']
 Family: binomial  ( logit )
Formula: donate_blood ~ age_std + gender + education_std + partner_status +      employment_status +
parental_status + type_of_community +
    mean_confidence_healthcare_agg_no_time_std + (1 | country) +      (1 | year_EVS)
   Data: data_full

     AIC      BIC   logLik deviance df.resid
 33442.9  33540.9 -16709.5  33418.9    26007

Scaled residuals:
   Min     1Q Median     3Q    Max
 -2.61  -0.79  -0.61   1.09   3.18

Random effects:
 Groups   Name        Variance Std.Dev.
 country  (Intercept) 0.097    0.31
 year_EVS (Intercept) 0.031    0.18
Number of obs: 26019, groups:  country, 28; year_EVS, 2

Fixed effects:
                                           Estimate Std. Error z value Pr(>|z|)
(Intercept)                                  -0.476      0.143    -3.3    9e-04 ***
age_std                                       0.093      0.024     3.8    1e-04 ***
gender1                                      -0.456      0.027   -17.1   <2e-16 ***
education_std                                 0.180      0.015    12.3   <2e-16 ***
partner_status1                               0.089      0.029     3.1    0.002 **
employment_status1                            0.250      0.031     8.1    5e-16 ***
```

```
parental_status1                              0.086      0.038     2.2    0.025 *
type_of_community1                           -0.025      0.033    -0.8    0.440
type_of_community2                           -0.059      0.035    -1.7    0.094 .
mean_confidence_healthcare_agg_no_time_std    0.036      0.059     0.6    0.545
---
Signif. codes:  0 ‘***’ 0.001 ‘**’ 0.01 ‘*’ 0.05 ‘.’ 0.1 ‘ ’ 1

Correlation of Fixed Effects:
            (Intr) ag_std gendr1 edctn_ prtn_1 empl_1 prnt_1 typ__1 typ__2
age_std      0.004
gender1     -0.115  0.017
educatn_std -0.001  0.109 -0.020
prtnr_stts1 -0.108  0.010  0.104 -0.023
emplymnt_s1 -0.080  0.428  0.068 -0.070 -0.107
prntl_stts1 -0.015  0.074 -0.086 -0.006 -0.228 -0.070
typ_f_cmmn1 -0.135 -0.017  0.007  0.059 -0.030  0.003 -0.020
typ_f_cmmn2 -0.123 -0.008  0.008  0.107 -0.046  0.010 -0.033  0.564
mn_cnf_____ -0.024 -0.013  0.002 -0.025  0.003  0.004 -0.003 -0.020 -0.029
> # Not significant
>
> # b) Healthcare quality
> m7 = glmer(donate_blood ~ age_std + gender + education_std + partner_status + employment_status +
parental_status + type_of_community + haq_agg_no_time_std + mean_confidence_healthcare_agg_no_time_std +
(1|country) + (1|year_haq) + (1|year_health_exp), data = data_full, family = "binomial")
> summary(m7)
Generalized linear mixed model fit by maximum likelihood (Laplace Approximation) ['glmerMod']
 Family: binomial  ( logit )
Formula: donate_blood ~ age_std + gender + education_std + partner_status +      employment_status +
parental_status + type_of_community +
    haq_agg_no_time_std + mean_confidence_healthcare_agg_no_time_std +      (1 | country) + (1 | year_haq) + (1 |
year_health_exp)
   Data: data_full

     AIC      BIC   logLik deviance df.resid
 33427.5  33541.8 -16699.8  33399.5    26005

Scaled residuals:
    Min      1Q  Median      3Q     Max
-2.4977 -0.7915 -0.6062  1.0918  3.0558

Random effects:
 Groups          Name        Variance Std.Dev.
 country         (Intercept) 0.097597 0.31241
 year_health_exp (Intercept) 0.002981 0.05459
 year_haq        (Intercept) 0.190637 0.43662
Number of obs: 26019, groups:  country, 28; year_health_exp, 20; year_haq, 6

Fixed effects:
                                           Estimate Std. Error z value Pr(>|z|)
(Intercept)                                -0.58381    0.19510  -2.992  0.00277 **
age_std                                    -0.10672    0.06027  -1.771  0.07663 .
gender1                                    -0.46357    0.02674 -17.337  < 2e-16 ***
education_std                               0.17484    0.01464  11.941  < 2e-16 ***
partner_status1                             0.05941    0.02979   1.994  0.04614 *
employment_status1                          0.21923    0.03403   6.442 1.18e-10 ***
parental_status1                            0.08248    0.04055   2.034  0.04198 *
type_of_community1                         -0.02735    0.03302  -0.828  0.40762
type_of_community2                         -0.06349    0.03546  -1.791  0.07337 .
haq_agg_no_time_std                         0.02012    0.07334   0.274  0.78378
mean_confidence_healthcare_agg_no_time_std  0.02644    0.07110   0.372  0.71001
---
Signif. codes:  0 ‘***’ 0.001 ‘**’ 0.01 ‘*’ 0.05 ‘.’ 0.1 ‘ ’ 1

Correlation of Fixed Effects:
            (Intr) ag_std gendr1 edctn_ prtn_1 empl_1 prnt_1 typ__1 typ__2 hq____
age_std      0.149
gender1     -0.084  0.022
educatn_std  0.003  0.073 -0.015
prtnr_stts1 -0.058  0.145  0.113 -0.010
emplymnt_s1 -0.081  0.100  0.091 -0.042 -0.045
prntl_stts1 -0.031 -0.045 -0.071  0.006 -0.201  0.019
typ_f_cmmn1 -0.099 -0.003  0.008  0.059 -0.027  0.008 -0.018
typ_f_cmmn2 -0.087  0.017  0.009  0.108 -0.040  0.013 -0.033  0.564
hq_gg_n_tm_  0.007 -0.003  0.008 -0.005  0.004  0.000  0.006  0.003  0.011
mn_cnf_____ -0.019 -0.004 -0.003 -0.019 -0.001  0.001 -0.006 -0.018 -0.031 -0.559
> print(summary(m7),digits=2)
Generalized linear mixed model fit by maximum likelihood (Laplace Approximation) ['glmerMod']
```

```
 Family: binomial  ( logit )
Formula: donate_blood ~ age_std + gender + education_std + partner_status +      employment_status +
parental_status + type_of_community +
    haq_agg_no_time_std + mean_confidence_healthcare_agg_no_time_std +      (1 | country) + (1 | year_haq) + (1 |
year_health_exp)
   Data: data_full

     AIC      BIC   logLik deviance df.resid
 33427.5  33541.8 -16699.8  33399.5    26005

Scaled residuals:
   Min     1Q Median     3Q    Max
 -2.50  -0.79  -0.61   1.09   3.06

Random effects:
 Groups          Name        Variance Std.Dev.
 country         (Intercept) 0.098    0.312
 year_health_exp (Intercept) 0.003    0.055
 year_haq        (Intercept) 0.191    0.437
Number of obs: 26019, groups:  country, 28; year_health_exp, 20; year_haq, 6

Fixed effects:
                                           Estimate Std. Error z value Pr(>|z|)
(Intercept)                                  -0.584      0.195    -3.0    0.003 **
age_std                                      -0.107      0.060    -1.8    0.077 .
gender1                                      -0.464      0.027   -17.3   <2e-16 ***
education_std                                 0.175      0.015    11.9   <2e-16 ***
partner_status1                               0.059      0.030     2.0    0.046 *
employment_status1                            0.219      0.034     6.4    1e-10 ***
parental_status1                              0.082      0.041     2.0    0.042 *
type_of_community1                           -0.027      0.033    -0.8    0.408
type_of_community2                           -0.063      0.035    -1.8    0.073 .
haq_agg_no_time_std                           0.020      0.073     0.3    0.784
mean_confidence_healthcare_agg_no_time_std    0.026      0.071     0.4    0.710
---
Signif. codes:  0 ‘***’ 0.001 ‘**’ 0.01 ‘*’ 0.05 ‘.’ 0.1 ‘ ’ 1

Correlation of Fixed Effects:
            (Intr) ag_std gendr1 edctn_ prtn_1 empl_1 prnt_1 typ__1 typ__2 hq____
age_std      0.149
gender1     -0.084  0.022
educatn_std  0.003  0.073 -0.015
prtnr_stts1 -0.058  0.145  0.113 -0.010
emplymnt_s1 -0.081  0.100  0.091 -0.042 -0.045
prntl_stts1 -0.031 -0.045 -0.071  0.006 -0.201  0.019
typ_f_cmmn1 -0.099 -0.003  0.008  0.059 -0.027  0.008 -0.018
typ_f_cmmn2 -0.087  0.017  0.009  0.108 -0.040  0.013 -0.033  0.564
hq_gg_n_tm_  0.007 -0.003  0.008 -0.005  0.004  0.000  0.006  0.003  0.011
mn_cnf_____ -0.019 -0.004 -0.003 -0.019 -0.001  0.001 -0.006 -0.018 -0.031 -0.559
> # Both not significant
```
