# Supplementary material for: How public trust and healthcare quality relate to blood donation behavior: Cross-cultural evidence
Source: J Health Psychol. 2023 Jun 5;29(1):3–14. doi: 10.1177/13591053231175809 (PMC10757392; doi:10.1177/13591053231175809)
Supplement: sj-pdf-6-hpq-10.1177_13591053231175809 – Supplemental material for How public trust and healthcare quality relate to blood donation behavior: Cross-cultural evidence [file sj-pdf-6-hpq-10.1177_13591053231175809.pdf]

```
######## Analysis script - P2 Trust in healthcare system, quality of healthcare, and blood donations  ########

##### Overview:

### 1) Preprocessing

### 2) Descriptives

# a) Summary statistics (individual-level and country-level)
# b) Map plots
# c) Density plots of indicators over time
# d) Time series plots of indicators over time
# e) Scatter plot of observed country-level mean blood donation rates
# f) Correlations
# g) Tests for variation (country-level; wave-level)

### 3) Mixed-effects models

# a) Intercept-only models
# b) Demographics
# c) Trust in healthcare system
# d) Healthcare quality

### 4) Predicted probabilities and plotting
# a) Trust
# b) HAQ
# c) Healthcare expenditures

### 5) Additional exploratory analyses
# a) Robustness check: Full model
# b) Robustness check: Subset of young respondents
# c) Robustness check: Analyses excluding each of the countries


###################################################################

# NOTE: We make use of the following secondary data sources,
# that need to be downloaded and saved in data/ before running the script:

# 1) Eurobarometer 82.2: European Commission, Brussels (2018). Eurobarometer 82.2 (2014). GESIS Data Archive, Cologne. ZA5931 Data file Version 3.0.0, https://doi.org/10.4232/1.12999
# Instructions: Download file ZA5931_v3-0-0.dta from https://doi.org/10.4232/1.12999 (free account with GESIS required)

# 2) Global Burden of Diseases Collaborative: Healthcare Access and Quality Index: Fullman, N., Yearwood, J., Abay, S. M., Abbafati, C., Abd-Allah, F., Abdela, J., ... & Chang, H. Y. (2018). Measuring performance on the Healthcare Access and Quality Index for 195 countries and territories and selected subnational locations: a systematic analysis from the Global Burden of Disease Study 2016. The Lancet, 391(10136), 2236-2271.
# Instructions: Download folder IHME_GBD_2015_HAQ_INDEX_1990_2015 from http://ghdx.healthdata.org/record/ihme-data/gbd-2015-healthcare-access-and-quality-index-1990-2015 (click on 'files'; free)

# 3) World Health Organization Global Health Expenditure database: World Health Organization. (2015). Global Health Expenditure Database (GHED). World Health Organization.
# Instructions: Download file total-healthcare-expenditure-as-share-of-national-gdp-by-country.csv from Our World in Data: https://ourworldindata.org/grapher/total-healthcare-expenditure-as-share-of-national-gdp-by-country (select 'Download' and then 'full data (CSV)'; free)

# 4) European Values Survey: EVS (2021): EVS Trend File 1981-2017. GESIS Data Archive, Cologne. ZA7503 Data file Version 2.0.0, https://doi.org/10.4232/1.13736.
# Instructions: Download zip folder ZA7503_v2-0-0.dta.zip from https://doi.org/10.4232/1.13736 (free account with GESIS required)

###################################################################

## This script requires rnaturalearth package version 0.1.0, please install this older version

# Install the 'remotes' package if not already installed
if (!requireNamespace("remotes", quietly = TRUE)) {
  install.packages("remotes")
}

# Load the 'remotes' package
library(remotes)

# Install an older version of rnaturalearth
install_version("rnaturalearth", version = "0.1.0")

##### Load packages
library("tidyverse")
library("lme4")
library("haven")
library("sjlabelled")
library("data.table")
library("scales")
library("RColorBrewer")
library("psych")
library("ggrepel")
library("sf")
library("rnaturalearth")
library("ggpubr")
library("corrplot")
library("Hmisc")
library("ggrepel")


##### Set WD

setwd("INSERT PATH TO YOUR WORKING DIRECTORY HERE")
```

```
##### Set seed for reproducibility
set.seed(101)

##### Load data sets

# Eurobarometer (2014 wave)
eurobarometer_data_raw = read_dta("../data/Eurobarometer/ZA5931_v3-0-0.dta")

# European Values Survey: Confidence in healthcare system
evs_raw = read_dta("../data/EVS/ZA7503_v2-0-0.dta/ZA7503_v2-0-0.dta")

# HAQ
haq_raw =
read.csv("../data/HAQ/IHME_GBD_2015_HAQ_INDEX_1990_2015/IHME_GBD_2015_HAQ_INDEX_1990_2015_HAQ_INDEX_AND_VALUES_Y2017M05D18.csv")

# Healthcare expenditures (OWID from World Bank - World Development Indicators)
health_exp_raw = read.csv("../data/OWID health expenditures/total-healthcare-expenditure-as-share-of-national-gdp-by-
country.csv")

# Country codes
iso_code_alpha2 = read.csv("../data/iso_code_alpha2.csv")

# UN_geoscheme_classification
UN_geoscheme_classification = read.csv("../data/UN_geoscheme_classification.csv")


###############################################################

####### 1) Preprocessing

### A) European Values Survey: Confidence in healthcare system

evs = dplyr::select(evs_raw, S007, S009, S020, E069_16)%>%
  rename(resp_num = S007, iso_code_alpha2 = S009, year = S020, confidence_healthcare = E069_16)%>%
  filter(confidence_healthcare > 0 & confidence_healthcare < 5 & year < 2014)%>%
  mutate(confidence_healthcare = ifelse(confidence_healthcare == 1, 4,
                                        ifelse(confidence_healthcare == 2, 3,
                                               ifelse(confidence_healthcare == 3, 2,
                                                      ifelse(confidence_healthcare == 4, 1, NA)))))%>% # reverse-code
  mutate(year = as.numeric(year),
         confidence_healthcare = as.numeric(confidence_healthcare))

# Filter by Eurobarometer countries
eurobarometer_countries = unique(eurobarometer_data_raw$isocntry)
eurobarometer_countries = ifelse(eurobarometer_countries == "DE-W", "DE",
                                 ifelse(eurobarometer_countries == "DE-E", "DE", eurobarometer_countries))

iso_code_alpha2 = iso_code_alpha2%>%
  mutate(country_name = ifelse(country_name == "United Kingdom of Great Britain and Northern Ireland", "United Kingdom",
                               ifelse(country_name == "Czechia", "Czech Republic", country_name)))

eurobarometer_countries_names = filter(iso_code_alpha2, iso_code_alpha2 %in% eurobarometer_countries | iso_code_alpha2 == "GB")

evs_filtered = filter(evs, iso_code_alpha2 %in% eurobarometer_countries)

evs_agg = evs_filtered%>%
  group_by(iso_code_alpha2, year)%>%
  summarise(mean_confidence_healthcare = mean(confidence_healthcare))%>%
  ungroup()


# Get UK estimate by weighting
# Weight UK EVS estimate by population (GB and NIR)
# Population UK (= GB + NIR): 58.68 mio. (1999); 61.81 mio. (2008)
# Population NIR: 1.679 mio. (1999); 1.77 mio. (2008)
# Population GB (calculated with UK - NIR): 57.001 mio. (1999); 60.04 mio. (2008)
mean_confidence_healthcare_UK_1999 = weighted.mean(c(2.634221, 2.832653), c(57.001, 1.679))
mean_confidence_healthcare_UK_2008 = weighted.mean(c(3.046572, 2.692929), c(60.04, 1.77))

evs_agg = evs_agg%>%
  add_row(iso_code_alpha2 = "GB", year = 1999, mean_confidence_healthcare = mean_confidence_healthcare_UK_1999)%>%
  add_row(iso_code_alpha2 = "GB", year = 2008, mean_confidence_healthcare = mean_confidence_healthcare_UK_2008)%>%
  add_row(iso_code_alpha2 = "CY", year = 1999, mean_confidence_healthcare = 2.662091)%>% # add dummy Cyprus data for year 1999
  filter(iso_code_alpha2 != "GB-NIR" & iso_code_alpha2 != "GB-GBN")%>%
  mutate(year = ifelse(year == 2009, 2008,
                       ifelse(year == 2000, 1999, year)))%>%
  rename(year_EVS = year)


### B) HAQ

haq = filter(haq_raw, indicator_name == "Healthcare Access and Quality", location_name %in%
eurobarometer_countries_names$country_name)%>%
  select(location_name, year_id, val)%>%
  rename(country_name = location_name, year_haq = year_id, haq_index = val)


### C) Healthcare expenditures

health_exp = mutate(health_exp_raw, Entity = ifelse(Entity == "Czechia", "Czech Republic", Entity))%>%
  filter(Entity %in% eurobarometer_countries_names$country_name)%>%
  select(Entity, Year, Health.expenditure..total....of.GDP.)%>%
  rename(country_name = Entity, year_health_exp = Year, health_exp_per_GDP = Health.expenditure..total....of.GDP.)


### D) Eurobarometer
```

```
# Select relevant variables
eurobarometer_data = dplyr::select(eurobarometer_data_raw, uniqid, nuts, nutslvl, isocntry, qe1_1, d15a, d40b, d25, d11, d8,
d10, d7)

# Remove labels
eurobarometer_data = remove_all_labels(eurobarometer_data)
eurobarometer_data = zap_formats(eurobarometer_data)

# Rename columns
eurobarometer_data = rename(eurobarometer_data,
                            donate_blood = qe1_1,
                            employment_status = d15a,
                            parental_status = d40b,
                            type_of_community = d25,
                            age = d11,
                            education = d8,
                            gender = d10,
                            partner_status = d7)

# Recode country code
eurobarometer_data$iso_code_alpha2 = ifelse(eurobarometer_data$isocntry == "DE-W", "DE",
                                        ifelse(eurobarometer_data$isocntry == "DE-E", "DE",
                                               ifelse(eurobarometer_data$isocntry == "GB-GBN", "GB",
                                                      ifelse(eurobarometer_data$isocntry == "GB-NIR", "GB",
eurobarometer_data$isocntry))))


# Variable transformations & converting variables to right format
data = eurobarometer_data %>%
  mutate(donate_blood = ifelse(donate_blood == 1 | donate_blood == 2, 1, 0),
         employment_status = ifelse(employment_status == 1 | employment_status == 2 | employment_status == 3 |
employment_status == 4, 0, 1),
         education = ifelse(education == 97, 0, ifelse(education == 98, age, education)),
         parental_status = ifelse(parental_status > 0, 1, 0),
         partner_status = ifelse(partner_status > 0 & partner_status < 9, 1, 0),
         type_of_community = ifelse(type_of_community == 1, 2, ifelse(type_of_community == 2, 1, ifelse(type_of_community == 3,
0, NA))),
         gender = gender - 1)%>%
  mutate(donate_blood = as.factor(donate_blood),
         type_of_community = as.factor(type_of_community),
         parental_status = as.factor(parental_status),
         gender = as.factor(gender),
         iso_code_alpha2 = as.factor(iso_code_alpha2),
         partner_status = as.factor(partner_status),
         employment_status = as.factor(employment_status))%>%
  mutate(index_time_r = ifelse(age < 66, round(2014 - (age - 18)/2, 0),
                               ifelse(age > 65, round(2014 - (65 - 18)/2 - (age - 65), 0), NA)),
         year_EVS = ifelse(index_time_r > 2003, 2008, 1999),
         year_haq = ifelse(index_time_r < 1993, 1990,
                          ifelse(index_time_r < 1998, 1995,
                                 ifelse(index_time_r < 2003, 2000,
                                        ifelse(index_time_r < 2008, 2005,
                                               ifelse(index_time_r < 2013, 2010,
                                                      ifelse(index_time_r < 2018, 2015, NA)))))),
         year_health_exp = ifelse(index_time_r < 1995, 1995, index_time_r))


### E) Merge country-level variables with individual-level data (Eurobarometer)

# Merge country-level variables with individual-level data (Eurobarometer)
data_full = left_join(data, eurobarometer_countries_names, by = "iso_code_alpha2")
data_full = left_join(data_full, evs_agg, by=c("year_EVS", "iso_code_alpha2"))
data_full = left_join(data_full, haq, by=c("year_haq", "country_name"))
data_full = left_join(data_full, health_exp, by=c("year_health_exp", "country_name"))
data_full = left_join(data_full, UN_geoscheme_classification, by="country_name")


### F) Add country-level IVs aggregated over years

# Calculate trust
evs_agg_no_time = evs_filtered%>%
  group_by(iso_code_alpha2)%>%
  summarise(mean_confidence_healthcare_agg_no_time = mean(confidence_healthcare))

mean_confidence_healthcare_UK = weighted.mean(c(2.886994, 2.785763), c(57.001, 1.679))
evs_agg_no_time = evs_agg_no_time%>% add_row(iso_code_alpha2 = "GB", mean_confidence_healthcare_agg_no_time =
mean_confidence_healthcare_UK)

# Calculate HAQ
haq_agg_no_time = haq%>%
  group_by(country_name)%>%
  summarise(haq_agg_no_time = mean(haq_index))

# Calculate expenditures
health_exp_agg_no_time = health_exp%>%
  group_by(country_name)%>%
  summarise(health_exp_per_GDP_agg_no_time = mean(health_exp_per_GDP))

# Merge with full data
data_full = left_join(data_full, evs_agg_no_time, by="iso_code_alpha2")
data_full = left_join(data_full, haq_agg_no_time, by="country_name")
data_full = left_join(data_full, health_exp_agg_no_time, by="country_name")

### Convert variables to right format
data_full = data_full%>%
  rename(country = country_name)%>%
  mutate(iso_code_alpha2 = as.factor(iso_code_alpha2),
         country = as.factor(country),
```

```
         UN_geoscheme_classification = as.factor(UN_geoscheme_classification))


### G) Exclude observations with NA in DV and respondents younger than 18

## Raw data: number of respondents
nrow(data_full) # 27868

# Remove invalid data
data_full = filter(data_full, !is.na(donate_blood))
nrow(data_full) # 27082

# Remove respondents under 18
data_full = filter(data_full, age > 17)
nrow(data_full) # 26532


################################################################

####### 2) Descriptives:

##### a) Summary statistics

# Individual-level
summary(data_full$donate_blood) # 0: 16337; 1: 10195
10195 / 26532 # 38.4%

describe(data_full$donate_blood) # mean: 0.38
describe(data_full$gender) # 0: 11653; 1: 14879
psych::describe(data_full$age) # mean 51.3; median 52; range 18 - 99
psych::describe(data_full$education) # mean 19.24; median 18; range 0 - 89; NA: 457
describe(data_full$partner_status) # 0: 9260 ; 1: 17216; NA: 56
describe(data_full$employment_status) # 0: 13638; 1: 12894
describe(data_full$parental_status) # 0: 21507 ; 1: 5020; NA: 5
describe(data_full$type_of_community) # 0: 7228; 1: 11153; 2: 8137; NA: 14

colSums(!is.na(data_full))

# Country-level
psych::describe(data_full$mean_confidence_healthcare)
psych::describe(data_full$haq_index)
psych::describe(data_full$health_exp_per_GDP)
psych::describe(evs_agg)
psych::describe(haq)
psych::describe(health_exp)


# Country-level indicator values and availability
country_level_indicator_descriptives = data_full%>%
  group_by(country)%>%
  dplyr::summarise(country_mean_confidence_healthcare = round(mean(mean_confidence_healthcare), 2),
                   sd_confidence_healthcare = round(sd(mean_confidence_healthcare), 2),
                   country_haq_index = round(mean(haq_index), 2),
                   sd_haq_index = round(sd(haq_index), 2),
                   country_health_exp_per_GDP = round(mean(health_exp_per_GDP), 2),
                   sd_health_exp_per_GDP = round(sd(health_exp_per_GDP), 2),
                   donate_blood = round(mean(as.numeric(as.character(donate_blood))), 2),
                   num_resp = n(),
                   UN_geoscheme_classification = unique(UN_geoscheme_classification))

# write.csv(country_level_indicator_descriptives, "country_level_indicator_descriptives.csv")


################################################################

##### b) Map plots

# Map plotting based on tutorial here: https://bhaskarvk.github.io/user2017.geodataviz/notebooks/02-Static-Maps.nb.html#

### Initiation of map data

# Get map data
world <- st_as_sf(rnaturalearth::countries110)
europe <- dplyr::filter(world, region_un=="Europe" & name!='Russia')

# Filter polygons that are part of continental Europe with the help of a bounding box
europe.bbox <- st_polygon(list(
  matrix(c(-25,29,45,29,45,75,-25,75,-25,29),byrow = T,ncol = 2)))

europe.clipped <- suppressWarnings(st_intersection(europe, st_sfc(europe.bbox, crs=st_crs(europe))))


## EVS
data_maps_evs = evs_agg%>%
  rename(iso_a2 = iso_code_alpha2)%>%
  mutate(iso_a2 = as.character(iso_a2),
         year_EVS = as.character(year_EVS))

mean_EVS = data_maps_evs%>% group_by(iso_a2)%>%dplyr::summarize(mean_confidence_healthcare =
mean(mean_confidence_healthcare))%>%mutate(year_EVS = "mean 1999-2008")

data_maps_evs = union(data_maps_evs, mean_EVS)

# Add to map data
map_data_evs = left_join(europe.clipped, data_maps_evs, by="iso_a2")%>%
  filter(!is.na(year_EVS))

# Two maps for mean and time_series
```

```
map_data_evs_mean = filter(map_data_evs, year_EVS == "mean 1999-2008")
map_data_evs_timeseries = filter(map_data_evs, year_EVS != "mean 1999-2008")

map_evs_mean = ggplot(data=subset(map_data_evs_mean, sovereignt != "Belarus" & sovereignt != "Ukraine" & sovereignt !=
"Moldova"), aes(fill=mean_confidence_healthcare)) +
  geom_sf(alpha=0.8,col='white') +
  coord_sf(crs="+proj=aea +lat_1=36.333333333333336 +lat_2=65.66666666666667 +lon_0=14") +
  viridis::scale_fill_viridis(name='', direction = 1, na.value = "grey92") +
  labs(x=NULL, y=NULL, title="Trust in the healthcare system")+
  theme(legend.position="bottom", legend.direction = "horizontal", legend.key.width = unit(1.3,"cm"), plot.title =
element_text(hjust = 0.5))

ggplot(data=subset(map_data_evs_timeseries, sovereignt != "Belarus" & sovereignt != "Ukraine" & sovereignt != "Moldova"),
aes(fill=mean_confidence_healthcare)) +
  geom_sf(alpha=0.8,col='white') +
  coord_sf(crs="+proj=aea +lat_1=36.333333333333336 +lat_2=65.66666666666667 +lon_0=14") +
  viridis::scale_fill_viridis(name='', direction = 1, na.value = "grey92") +
  labs(x=NULL, y=NULL, title="Trust in the healthcare system")+
  facet_grid(~ year_EVS)+
  theme(legend.position="bottom", legend.direction = "horizontal", legend.key.width = unit(1.3,"cm"), plot.title =
element_text(hjust = 0.5))

#ggsave("plots/maps/trust_timeseries.png", width = 11.9, height = 7.5, units = "in")


## HAQ
haq = left_join(haq, eurobarometer_countries_names, by="country_name")%>%
  select(-country_name)

data_maps_haq = haq%>%
  rename(iso_a2 = iso_code_alpha2)%>%
  mutate(iso_a2 = as.character(iso_a2),
         year_haq = as.character(year_haq))

mean_haq = data_maps_haq%>% group_by(iso_a2)%>%dplyr::summarize(haq_index = mean(haq_index))%>%mutate(year_haq = "mean 1990-
2015")

data_maps_haq = union(data_maps_haq, mean_haq)

# Add to map data
map_data_haq = left_join(europe.clipped, data_maps_haq, by="iso_a2")%>%
  filter(!is.na(year_haq))

# Two maps for mean and time_series
map_data_haq_mean = filter(map_data_haq, year_haq == "mean 1990-2015")
map_data_haq_timeseries = filter(map_data_haq, year_haq != "mean 1990-2015")

map_haq_mean = ggplot(data=subset(map_data_haq_mean, sovereignt != "Belarus" & sovereignt != "Ukraine" & sovereignt !=
"Moldova"), aes(fill=haq_index)) +
  geom_sf(alpha=0.8,col='white') +
  coord_sf(crs="+proj=aea +lat_1=36.333333333333336 +lat_2=65.66666666666667 +lon_0=14") +
  viridis::scale_fill_viridis(name='', direction = 1, na.value = "grey92") +
  labs(x=NULL, y=NULL, title="HAQ index")+
  theme(legend.position="bottom", legend.direction = "horizontal", legend.key.width = unit(1.3,"cm"), plot.title =
element_text(hjust = 0.5))

ggplot(data=subset(map_data_haq_timeseries, sovereignt != "Belarus" & sovereignt != "Ukraine" & sovereignt != "Moldova"),
aes(fill=haq_index)) +
  geom_sf(alpha=0.8,col='white') +
  coord_sf(crs="+proj=aea +lat_1=36.333333333333336 +lat_2=65.66666666666667 +lon_0=14") +
  viridis::scale_fill_viridis(name='', direction = 1, na.value = "grey92") +
  labs(x=NULL, y=NULL, title="HAQ index")+
  facet_wrap(~ year_haq, ncol=3)+
  theme(legend.position="bottom", legend.direction = "horizontal", legend.key.width = unit(1.3,"cm"), plot.title =
element_text(hjust = 0.5))

#ggsave("plots/maps/haq_timeseries.png", width = 8.75, height = 8, units = "in")


## health_exp
health_exp = left_join(health_exp, eurobarometer_countries_names, by="country_name")%>%
  select(-country_name)

data_maps_health_exp = health_exp%>%
  rename(iso_a2 = iso_code_alpha2)%>%
  mutate(iso_a2 = as.character(iso_a2),
         year_health_exp = as.character(year_health_exp))

mean_health_exp = data_maps_health_exp%>% group_by(iso_a2)%>%dplyr::summarize(health_exp_per_GDP =
mean(health_exp_per_GDP))%>%mutate(year_health_exp = "mean 1995-2014")

data_maps_health_exp = union(data_maps_health_exp, mean_health_exp)

# Add to map data
map_data_health_exp = left_join(europe.clipped, data_maps_health_exp, by="iso_a2")%>%
  filter(!is.na(year_health_exp))

# Two maps for mean and time_series
map_data_health_exp_mean = filter(map_data_health_exp, year_health_exp == "mean 1995-2014")
map_data_health_exp_timeseries = filter(map_data_health_exp, year_health_exp != "mean 1995-2014")

map_health_exp_mean = ggplot(data=subset(map_data_health_exp_mean, sovereignt != "Belarus" & sovereignt != "Ukraine" &
sovereignt != "Moldova"), aes(fill=health_exp_per_GDP)) +
  geom_sf(alpha=0.8,col='white') +
  coord_sf(crs="+proj=aea +lat_1=36.333333333333336 +lat_2=65.66666666666667 +lon_0=14") +
  viridis::scale_fill_viridis(name='', direction = 1, na.value = "grey92") +
  labs(x=NULL, y=NULL, title="Health expenditures per GDP")+
  theme(legend.position="bottom", legend.direction = "horizontal", legend.key.width = unit(1.3,"cm"), plot.title =
```

```
element_text(hjust = 0.5))


ggplot(data=subset(map_data_health_exp_timeseries, sovereignt != "Belarus" & sovereignt != "Ukraine" & sovereignt !=
"Moldova"), aes(fill=health_exp_per_GDP)) +
  geom_sf(alpha=0.8,col='white') +
  coord_sf(crs="+proj=aea +lat_1=36.333333333333336 +lat_2=65.66666666666667 +lon_0=14") +
  viridis::scale_fill_viridis(name='', direction = 1, na.value = "grey92") +
  labs(x=NULL, y=NULL, title="Health expenditures per GDP")+
  facet_wrap(~ year_health_exp, ncol=5)+
  theme(legend.position="bottom", legend.direction = "horizontal", legend.key.width = unit(1.3,"cm"), plot.title =
element_text(hjust = 0.5))

#ggsave("plots/maps/health_exp_timeseries.png", width = 12.1, height = 10.8, units = "in")


## Blood donation

# Calculate country-level means
blood_donation_agg = data_full%>%
  group_by(iso_code_alpha2)%>%
  dplyr::summarize(donate_blood_agg = mean(as.numeric(as.character(donate_blood)), na.rm=T))%>%
  rename(iso_a2 = iso_code_alpha2)%>%
  mutate(iso_a2 = as.character(iso_a2))

map_data_blood_donation = left_join(europe.clipped, blood_donation_agg, by="iso_a2")

ggplot(map_data_blood_donation, aes(fill=donate_blood_agg)) +
  geom_sf(alpha=0.8,col='white') +
  coord_sf(crs="+proj=aea +lat_1=36.333333333333336 +lat_2=65.66666666666667 +lon_0=14") +
  viridis::scale_fill_viridis(name='Country-level mean\nblood donation', direction = -1, labels=scales::percent,
option="plasma") +
  labs(x=NULL, y=NULL, title=NULL)

#ggsave("plots/maps/blood_donation.png", width = 7, height = 5.7, units = "in")


################################################################

##### c) Density plots of indicators over time

# A) Trust
evs_agg$year_EVS = as.factor(evs_agg$year_EVS)

density1 = ggplot(data=evs_agg, aes(mean_confidence_healthcare, color = year_EVS, fill = year_EVS)) +
  geom_density(alpha = 0.4)+
  guides(fill = "none")+
  labs(x = "Trust in the healthcare system", color="Year")

# B) HAQ
haq$year_haq = as.factor(haq$year_haq)

density2 = ggplot(data=haq, aes(haq_index, color = year_haq, fill = year_haq)) +
  geom_density(alpha = 0.2)+
  guides(fill = "none")+
  labs(x = "HAQ index", color="Year")

# C) Health expenditures
health_exp$year_health_exp = as.factor(health_exp$year_health_exp)

density3 = ggplot(data=health_exp, aes(health_exp_per_GDP, color = year_health_exp, fill = year_health_exp)) +
  geom_density(alpha = 0.08)+
  guides(fill = "none")+
  labs(x = "Health expenditures per GDP", color="Year")

# D) Histogram of index_time_r
ggplot(data=data_full, aes(index_time_r)) +
  geom_histogram(bins=20) +
  labs(x = "Assumed time point of donation", y="Number of respondents")

# ggsave("plots/density/index_time_r.png", width = 8.4, height = 7, units = "in")

# E) Make combined plot for paper
ggarrange(density1, density2, density3,
          labels = c("A", "B", "C"),
          ncol = 3, nrow = 1)

#ggsave("plots/density/combined_indicators_density.png", width = 15.3, height = 6, units = "in")


################################################################

##### d) Time series plots of indicators over time

# A) Trust
evs_agg = left_join(evs_agg, eurobarometer_countries_names, by="iso_code_alpha2")

ggplot(evs_agg, aes(year_EVS, mean_confidence_healthcare, color=iso_code_alpha2, group = iso_code_alpha2, label=country_name))
+
  geom_line(aes()) +
  geom_text_repel() +
  labs(y = "Trust in the healthcare system", x = "Survey wave") +
  guides(color = "none")+
  ylim(1, 4)

#ggsave("plots/line/trust_timeseries_fullscale.png", width = 7, height = 12, units = "in")

# B) HAQ
```

```
haq = left_join(haq, eurobarometer_countries_names, by="iso_code_alpha2")
haq$country_name_alt = ifelse(haq$year_haq == 1990 | haq$year_haq == 2015, haq$country_name, "")

ggplot(haq, aes(year_haq, haq_index, color=iso_code_alpha2, group = iso_code_alpha2, label=country_name_alt)) +
  geom_line(aes()) +
  geom_text_repel(max.overlaps =24) +
  labs(y = "HAQ index", x = "Survey wave") +
  guides(color = "none")+
  ylim(0, 100)

#ggsave("plots/line/haq_timeseries_fullscale.png", width = 11.4, height = 15, units = "in")

# C) Health expenditures
health_exp = left_join(health_exp, eurobarometer_countries_names, by="iso_code_alpha2")
health_exp$country_name_alt = ifelse(health_exp$year_health_exp == 1995 | health_exp$year_health_exp == 2014,
health_exp$country_name, "")

ggplot(health_exp, aes(year_health_exp, health_exp_per_GDP, color=iso_code_alpha2, group = iso_code_alpha2,
label=country_name_alt)) +
  geom_line(aes()) +
  geom_text_repel() +
  labs(y = "Health expenditures per GDP", x = "Survey wave") +
  guides(color = "none")

#ggsave("plots/line/health_exp_timeseries.png", width = 11.4, height = 17, units = "in")

ggplot(health_exp, aes(year_health_exp, health_exp_per_GDP, color=iso_code_alpha2, group = iso_code_alpha2,
label=country_name_alt)) +
  geom_smooth(method = "glm", alpha=0.2) +
  geom_text_repel() +
  labs(y = "Health expenditures per GDP", x = "Survey wave") +
  guides(color = "none")

#ggsave("plots/line/health_exp_timeseries_smooth.png", width = 9, height = 14, units = "in")


#################################################################

##### e) Scatter plot of observed country-level mean blood donation rates

# To distinguish different European regions, include as aesthetic ", color=UN_geoscheme_classification"

# Scatter plot: mean_confidence_healthcare
observed_country_trust = ggplot(country_level_indicator_descriptives, aes(country_mean_confidence_healthcare, donate_blood)) +
  stat_smooth(method="glm", formula=y~x, alpha=0.2, size=2) +
  geom_point() +
  geom_text(aes(label=country), color = "black", hjust=-0.08, vjust=0) +
  labs(y = "Observed country-level mean levels of blood donation", x = "Trust in the healthcare system") +
  theme(legend.position="bottom", plot.title = element_text(hjust = 0.5))

# Scatter plot: HAQ
observed_country_haq = ggplot(country_level_indicator_descriptives, aes(country_haq_index, donate_blood)) +
  stat_smooth(method="glm", formula=y~x, alpha=0.2, size=2) +
  geom_point() +
  geom_text(aes(label=country), color = "black", hjust=-0.08, vjust=0) +
  labs(y = "Observed country-level mean levels of blood donation", x = "HAQ index") +
  theme(legend.position="bottom", plot.title = element_text(hjust = 0.5))

# Scatter plot: health_exp_per_GDP
observed_country_exp = ggplot(country_level_indicator_descriptives, aes(country_health_exp_per_GDP, donate_blood)) +
  stat_smooth(method="glm", formula=y~x, alpha=0.2, size=2) +
  geom_point() +
  geom_text(aes(label=country), color = "black", hjust=-0.08, vjust=0) +
  labs(y = "Observed country-level mean levels of blood donation", x = "Health expenditures per GDP") +
  theme(legend.position="bottom", plot.title = element_text(hjust = 0.5))

# make combined descriptves plot for paper SI
ggarrange(observed_country_trust, observed_country_haq, observed_country_exp,
          labels = c("A", "B", "C"),
          ncol = 3, nrow = 1)

#ggsave("plots/scatter/combined_descr.png", width = 15.6, height = 7.49, units = "in")

#################################################################

##### f) Correlations

### Plot correlations
data_variables_of_interest = select(data_full, donate_blood, age, gender, education, partner_status, employment_status,
parental_status, type_of_community, mean_confidence_healthcare, haq_index, health_exp_per_GDP)%>%
  # delete categorical variables with more than two levels
  select(-type_of_community)%>%
  # convert all (binary) factor variables to numeric (Note that we calculate a Pearson correlation if a categorical variable
has a 0/1-coding (--> point-biserial correlation coefficient))
  mutate_if(is.factor, as.character)%>%
  mutate_if(is.character, as.numeric)%>%
  # Rename as in model
  rename(`Blood donation` = donate_blood,
         `Gender (female = 1)` = gender,
         `Age (years)` = age,
         `Education (years)` = education,
         `Partner status` = partner_status,
         `Employment status` = employment_status,
         `Parental status` = parental_status,
         `Country-level trust in the healthcare system` = mean_confidence_healthcare,
         `Country-level HAQ index` = haq_index,
         `Country-level healthcare expenditures per GDP` = health_exp_per_GDP)
```

```
correlations = cor(data_variables_of_interest, use = "complete.obs", method = "pearson")
corrplot(correlations, type="lower", col=brewer.pal(n=8, name="RdYlBu"))

### Indicator correlations

# a) Country-level
cor.test(country_level_indicator_descriptives$country_mean_confidence_healthcare,
country_level_indicator_descriptives$country_haq_index, method = "pearson")
# 0.48, p < 0.05
cor.test(country_level_indicator_descriptives$country_mean_confidence_healthcare,
country_level_indicator_descriptives$country_health_exp_per_GDP, method = "pearson")
# 0.29, p = 0.137
cor.test(country_level_indicator_descriptives$country_haq_index,
country_level_indicator_descriptives$country_health_exp_per_GDP, method = "pearson")
# 0.69, p < 0.0001

# b) Individual-level
cor.test(data_full$mean_confidence_healthcare, data_full$haq_index, method = "pearson")
# 0.30, p < 0.0001
cor.test(data_full$mean_confidence_healthcare, data_full$health_exp_per_GDP, method = "pearson")
# 0.26, p < 0.0001
cor.test(data_full$haq_index, data_full$health_exp_per_GDP, method = "pearson")
# 0.71, p < 0.0001


################################################################

##### g) Tests for variation

## 1) Test for country-level variation (using proportions test (see https://sphweb.bumc.bu.edu/otlt/MPH-
Modules/BS/R/R6_CategoricalDataAnalysis/R6_CategoricalDataAnalysis6.html))

# Calculate sums
prop_test = country_level_indicator_descriptives%>%
  mutate(country_haq_index_recoded = country_haq_index/100,
         country_health_exp_per_GDP_recoded = country_health_exp_per_GDP/100,
         country_mean_confidence_healthcare_recoded = scales::rescale(country_mean_confidence_healthcare, to = c(0, 1)))%>%
  mutate(sum_trust = round(country_mean_confidence_healthcare_recoded * num_resp, 0),
         sum_haq = round(country_haq_index_recoded * num_resp, 0),
         sum_exp = round(country_health_exp_per_GDP_recoded * num_resp, 0))

prop.test(prop_test$sum_trust, prop_test$num_resp)
# X-squared(27) = 7434, p < 0.001
prop.test(prop_test$sum_haq, prop_test$num_resp)
# X-squared(27) = 429.51, p < 0.001
prop.test(prop_test$sum_exp, prop_test$num_resp)
# X-squared(27) = 78.711, p < 0.001


## 2) Test for wave-level variation: Relationship between survey wave and macro-level indicators

# Doesn't work for trust, because only 2 time points

indicator_descriptives_across_HAQ_years = haq%>%
  mutate(year_haq = as.numeric(as.character(year_haq)))%>%
  group_by(year_haq)%>%
  dplyr::summarise(wave_haq_index = round(mean(haq_index), 2))

indicator_descriptives_across_health_exp_years = health_exp%>%
  mutate(year_health_exp = as.numeric(as.character(year_health_exp)))%>%
  group_by(year_health_exp)%>%
  dplyr::summarise(wave_health_exp_per_GDP = round(mean(health_exp_per_GDP), 2))

cor.test(indicator_descriptives_across_HAQ_years$wave_haq_index, indicator_descriptives_across_HAQ_years$year_haq, method =
"pearson")
# 0.99, p < 0.001

cor.test(indicator_descriptives_across_health_exp_years$wave_health_exp_per_GDP,
indicator_descriptives_across_health_exp_years$year_health_exp, method = "pearson")
# 0.97, p < 0.001

### Examine also trends at the country level

# Trust
evs_spread = evs_agg%>%
  select(-iso_code_alpha2)%>%
  mutate(year_EVS = ifelse(year_EVS == 1999, "y1999", "y2008"))%>%
  spread(year_EVS, mean_confidence_healthcare)%>%
  mutate(change = y2008 - y1999)

# in 11/28 countries: increase in trust over time
# in 17/28 countries: decrease in trust over time

# HAQ
haq_spread = haq%>%
  select(-iso_code_alpha2)%>%
  spread(year_haq, haq_index)

# For all countries but 3 (Lithuania, Estonia, Latvia) continuous increase in HAQ index over time
# For all countries continuous increase from 1995 onwards

# Health exp
health_exp_spread = health_exp%>%
  select(-iso_code_alpha2, -country_name_alt)%>%
  spread(year_health_exp, health_exp_per_GDP)

# Quite some fluctuation across individual years
# When examining smoothed relationship of time on health_exp, all countries show positive trend (most constant relationship in
```

```
Estonia)


################################################################

####### 3) Mixed-effects models

# Due to problems with convergence in some models, we normalized the continuous variables (age, education)
# (see https://rstudio-pubs-static.s3.amazonaws.com/33653_57fc7b8e5d484c909b615d8633c01d51.html)
data_full$age_std = as.numeric(scale(data_full$age))
data_full$education_std = as.numeric(scale(data_full$education))
data_full$haq_index_std = as.numeric(scale(data_full$haq_index))
data_full$health_exp_per_GDP_std = as.numeric(scale(data_full$health_exp_per_GDP))
data_full$mean_confidence_healthcare_std = as.numeric(scale(data_full$mean_confidence_healthcare))

# Convert year variables to factor
data_full$year_health_exp = as.factor(data_full$year_health_exp)
data_full$year_haq = as.factor(data_full$year_haq)
data_full$year_EVS = as.factor(data_full$year_EVS)

##### a) Intercept-only models (empty models)

# 0) Only including random effect of country
m0 = glmer(donate_blood ~ (1|country), data = data_full, family = "binomial")
summary(m0)
# AIC = 34848.6

# 0A) Only including random effect of time (EVS)
m0a = glmer(donate_blood ~ (1|year_EVS), data = data_full, family = "binomial")
summary(m0a)
# AIC = 35205.7

# 0B) Only including random effect of time (HAQ)
m0b = glmer(donate_blood ~ (1|year_haq), data = data_full, family = "binomial")
summary(m0b)
# AIC = 35136.0

# 0C) Only including random effect of time (health_exp)
m0c = glmer(donate_blood ~ (1|year_health_exp), data = data_full, family = "binomial")
summary(m0c)
# AIC = 35167.3

# Significance of random effects
m0_constrained = glm(donate_blood ~ 1, data = data_full, family = "binomial")
summary(m0_constrained)
# AIC = 35348

anova(m0, m0_constrained)
# LL-test significant: country random effect improves model fit
# chi^2(1) = 501.79, p < 0.001

anova(m0a, m0_constrained)
# LL-test significant: time (EVS) random effect improves model fit
# chi^2(1) = 144.6, p < 0.001

anova(m0b, m0_constrained)
# LL-test significant: time (HAQ) random effect improves model fit
# chi^2(1) = 214.33, p < 0.001

anova(m0c, m0_constrained)
# LL-test significant: time (health exp) random effect improves model fit
# chi^2(1) = 183.07, p < 0.001


##### b) Demographics

m1 = glmer(donate_blood ~ age_std + gender + education_std + partner_status + employment_status + parental_status +
type_of_community + (1|country), data = data_full, family = "binomial")
summary(m1)
print(summary(m1),digits=2)
# AIC = 33481.3 (< 34848.6 --> demographics improve model fit)
# significant effects: age (older more), gender (male more), education (higher education more), partner_status (with partner
more), employment_status1 (employed more)


##### c) Trust in healthcare system

m2 = glmer(donate_blood ~ age_std + gender + education_std + partner_status + employment_status + parental_status +
type_of_community + mean_confidence_healthcare_std + (1|country) + (1|year_EVS), data = data_full, family = "binomial")
summary(m2)
print(summary(m2),digits=2)
# significant effects: mean_confidence_healthcare (higher trust MORE)
# AIC: 33431.3


##### d) Healthcare quality

m3 = glmer(donate_blood ~ age_std + gender + education_std + partner_status + employment_status + parental_status +
type_of_community + haq_index_std + health_exp_per_GDP_std + (1|country) + (1|year_haq) + (1|year_health_exp), data =
data_full, family = "binomial")
summary(m3)
print(summary(m3),digits=2)
# MARGINALLY significant effect: haq_index (higher HAQ MORE)
# AIC: 33424.7


## a) Only HAQ
```

```
m3a = glmer(donate_blood ~ haq_index_std + age_std + gender + education_std + partner_status + employment_status +
parental_status + type_of_community + (1|country) + (1|year_haq), data = data_full, family = "binomial")
summary(m3a)
# MARGINALLY significant effect: haq_index (higher HAQ MORE)

## b) Only Health exp
m3b = glmer(donate_blood ~ health_exp_per_GDP_std + age_std + gender + education_std + partner_status + employment_status +
parental_status + type_of_community + (1|country) + (1|year_health_exp), data = data_full, family = "binomial")
summary(m3b)
# not significant


################################################################

####### 4) Predicted probabilities and plotting

# Prediction intervals are generated based on tutorial here: https://cran.r-
project.org/web/packages/merTools/vignettes/Using_predictInterval.html

library("merTools")

##### a) Trust

newdata <- expand.grid(mean_confidence_healthcare_std = sort(unique(data_full$mean_confidence_healthcare_std)),
                       age_std = mean(data_full$age_std, na.rm=TRUE),
                       gender = "1",
                       education_std = mean(data_full$education_std, na.rm=TRUE),
                       partner_status="1",
                       employment_status = "1",
                       parental_status = "0",
                       type_of_community = "1",
                       country = "new country",
                       year_EVS = "new year")

PI <- predictInterval(merMod = m2, newdata = newdata, level = 0.8, n.sims = 1000, stat = "mean", type="probability",
include.resid.var = F)

data_with_PI = bind_cols(newdata, PI)

a_with_CI = ggplot(data_with_PI, aes(x = mean_confidence_healthcare_std, y=fit, ymin=lwr, ymax=upr)) +
  geom_smooth(aes(ymin = lwr, ymax = upr), stat = "identity") +
  labs(y = "Predicted probability of blood donation", x = "Trust in the healthcare system (normalized)") +
  theme(legend.position="bottom", plot.title = element_text(hjust = 0.5))+
  expand_limits(y = 0)


##### b) HAQ

newdata <- expand.grid(haq_index_std = sort(unique(data_full$haq_index_std)),
                       age_std = mean(data_full$age_std, na.rm=TRUE),
                       gender = "1",
                       education_std = mean(data_full$education_std, na.rm=TRUE),
                       partner_status="1",
                       employment_status = "1",
                       parental_status = "0",
                       type_of_community = "1",
                       country = "new country",
                       year_haq = "new year")

PI <- predictInterval(merMod = m3a, newdata = newdata, level = 0.8, n.sims = 1000, stat = "mean", type="probability",
include.resid.var = F)

data_with_PI = bind_cols(newdata, PI)

b_with_CI = ggplot(data_with_PI, aes(x = haq_index_std, y=fit, ymin=lwr, ymax=upr)) +
  geom_smooth(aes(ymin = lwr, ymax = upr), stat = "identity") +
  labs(y = "Predicted probability of blood donation", x = "HAQ index (normalized)") +
  theme(legend.position="bottom", plot.title = element_text(hjust = 0.5))+
  expand_limits(y = 0)


##### c) Healthcare expenditures

newdata <- expand.grid(health_exp_per_GDP_std = sort(unique(data_full$health_exp_per_GDP_std)),
                       age_std = mean(data_full$age_std, na.rm=TRUE),
                       gender = "1",
                       education_std = mean(data_full$education_std, na.rm=TRUE),
                       partner_status="1",
                       employment_status = "1",
                       parental_status = "0",
                       type_of_community = "1",
                       country = "new country",
                       year_health_exp = "new year")

PI <- predictInterval(merMod = m3b, newdata = newdata, level = 0.8, n.sims = 1000, stat = "mean", type="probability",
include.resid.var = F)

data_with_PI = bind_cols(newdata, PI)

c_with_CI = ggplot(data_with_PI, aes(x = health_exp_per_GDP_std, y=fit, ymin=lwr, ymax=upr)) +
  geom_smooth(aes(ymin = lwr, ymax = upr), stat = "identity") +
  labs(y = "Predicted probability of blood donation", x = "Healthcare expenditures (normalized)") +
  theme(legend.position="bottom", plot.title = element_text(hjust = 0.5))+
  expand_limits(y = 0)


# make combined plot for paper
ggarrange(a_with_CI, b_with_CI, c_with_CI,
```

```
         labels = c("A", "B", "C"),
         ncol = 3, nrow = 1)

#ggsave("plots/scatter/combined_pred_horizontal.png", width = 13, height = 6.4, units = "in")
#ggsave(file="plots/scatter/combined_pred_horizontal.pdf")


##############################################################################

####### 5) Additional exploratory analyses

##### a) Robustness check: Full model

# 1) Full model with survey wave random effects
m_full = glmer(donate_blood ~ age_std + gender + education_std + partner_status + employment_status + parental_status +
type_of_community + mean_confidence_healthcare_std + haq_index_std + health_exp_per_GDP_std + (1|country) + (1|year_EVS) +
(1|year_haq) + (1|year_health_exp), data = data_full, family = "binomial")
summary(m_full)
print(summary(m_full),digits=2)
# significant effects: mean_confidence_healthcare (higher trust MORE)
# HAQ and exp not significant

# 2) Full model without survey wave random effects
m_full_no_RE_wave = glmer(donate_blood ~ age_std + gender + education_std + partner_status + employment_status +
parental_status + type_of_community + mean_confidence_healthcare_std + haq_index_std + health_exp_per_GDP_std + (1|country),
data = data_full, family = "binomial")
summary(m_full_no_RE_wave)
print(summary(m_full_no_RE_wave),digits=2)


##### b) Robustness check: Subset of young respondents

data_young = filter(data_full, age < 44)
# With age < 58, all group n > 250
# With age < 44, all group n > 250 (EXCEPT small samples: Malta, Cyprus, Luxembourg)

data_young_resp_per_country = data_young%>%
  group_by(country)%>%
  dplyr::summarise(num_resp = n())

# Descriptives
describe(data_young$donate_blood) # mean: 0.34
describe(data_young$gender) # mean: 0.56
psych::describe(data_young$age) # mean 31.82; median 32; range 18 - 43
psych::describe(data_young$education) # mean 20.26; median 19; range 0 - 43; NA: 188
describe(data_young$partner_status) # mean: 0.65
describe(data_young$employment_status) # mean: 0.68
describe(data_young$parental_status) # mean: 0.42
describe(data_young$type_of_community) # 0: 2899 (0.30); 1: 4030 (0.42); 2: 2706 (0.28)

# Summary statistics (country-level)
psych::describe(data_young$mean_confidence_healthcare)
psych::describe(data_young$haq_index)
psych::describe(data_young$health_exp_per_GDP)


## 1) Mixed-effects models

# a) Trust
m4 = glmer(donate_blood ~ age_std + gender + education_std + partner_status + employment_status + parental_status +
type_of_community + mean_confidence_healthcare_std + (1|country) + (1|year_EVS), data = data_young, family = "binomial")
summary(m4)
print(summary(m4),digits=1)
# Not significant

# b) Healthcare quality
m5 = glmer(donate_blood ~ age_std + gender + education_std + partner_status + employment_status + parental_status +
type_of_community + haq_index_std + health_exp_per_GDP_std + (1|country) + (1|year_haq) + (1|year_health_exp), data =
data_young, family = "binomial")
summary(m5)
print(summary(m5),digits=1)
# Both not significant

# m_full_young does not converge
# m_full_young = glmer(donate_blood ~ age_std + gender + education_std + partner_status + employment_status + parental_status +
type_of_community + mean_confidence_healthcare_std + haq_index_std + health_exp_per_GDP_std + (1|country) + (1|year_EVS) +
(1|year_haq)  + (1|year_health_exp), data = data_young, family = "binomial")


# 2) Map plots
mean_indicators_young = data_young%>%
  group_by(iso_code_alpha2)%>%
  dplyr::summarize(mean_confidence_healthcare = mean(mean_confidence_healthcare),
                   haq_index = mean(haq_index),
                   health_exp_per_GDP = mean(health_exp_per_GDP))%>%
  rename(iso_a2 = iso_code_alpha2)

# Add to map data
map_data_young = left_join(europe.clipped, mean_indicators_young, by="iso_a2")

trust_young = ggplot(data=subset(map_data_young, sovereignt != "Belarus" & sovereignt != "Ukraine" & sovereignt != "Moldova"),
aes(fill=mean_confidence_healthcare)) +
  geom_sf(alpha=0.8,col='white') +
  coord_sf(crs="+proj=aea +lat_1=36.333333333333336 +lat_2=65.66666666666667 +lon_0=14") +
  viridis::scale_fill_viridis(name='', direction = 1, na.value = "grey92") +
  labs(x=NULL, y=NULL, title="Trust in the healthcare system")+
  theme(legend.position="bottom", legend.direction = "horizontal", legend.key.width = unit(1.3,"cm"), plot.title =
element_text(hjust = 0.5))
```

```
haq_young = ggplot(data=subset(map_data_young, sovereignt != "Belarus" & sovereignt != "Ukraine" & sovereignt != "Moldova"),
aes(fill=haq_index)) +
  geom_sf(alpha=0.8,col='white') +
  coord_sf(crs="+proj=aea +lat_1=36.333333333333336 +lat_2=65.66666666666667 +lon_0=14") +
  viridis::scale_fill_viridis(name='', direction = 1, na.value = "grey92") +
  labs(x=NULL, y=NULL, title="HAQ index")+
  theme(legend.position="bottom", legend.direction = "horizontal", legend.key.width = unit(1.3,"cm"), plot.title =
element_text(hjust = 0.5))

health_exp_young = ggplot(data=subset(map_data_young, sovereignt != "Belarus" & sovereignt != "Ukraine" & sovereignt !=
"Moldova"), aes(fill=health_exp_per_GDP)) +
  geom_sf(alpha=0.8,col='white') +
  coord_sf(crs="+proj=aea +lat_1=36.333333333333336 +lat_2=65.66666666666667 +lon_0=14") +
  viridis::scale_fill_viridis(name='', direction = 1, na.value = "grey92") +
  labs(x=NULL, y=NULL, title="Health expenditures per GDP")+
  theme(legend.position="bottom", legend.direction = "horizontal", legend.key.width = unit(1.3,"cm"), plot.title =
element_text(hjust = 0.5))

ggarrange(trust_young, haq_young, health_exp_young,
          labels = c("A", "B", "C"),
          ncol = 3, nrow = 1)

#ggsave("plots/maps/combined_indicators_young.png", width = 14, height = 6.97, units = "in")


##### c) Robustness check: Analyses excluding each of the countries

excluding_Austria = filter(data_full, country != "Austria")
excluding_Belgium = filter(data_full, country != "Belgium")
excluding_Bulgaria = filter(data_full, country != "Bulgaria")
excluding_Croatia = filter(data_full, country != "Croatia")
excluding_Cyprus = filter(data_full, country != "Cyprus")
excluding_Czech_Republic = filter(data_full, country != "Czech Republic")
excluding_Denmark = filter(data_full, country != "Denmark")
excluding_Estonia = filter(data_full, country != "Estonia")
excluding_Finland = filter(data_full, country != "Finland")
excluding_France = filter(data_full, country != "France")
excluding_Germany = filter(data_full, country != "Germany")
excluding_Greece = filter(data_full, country != "Greece")
excluding_Hungary = filter(data_full, country != "Hungary")
excluding_Ireland = filter(data_full, country != "Ireland")
excluding_Italy = filter(data_full, country != "Italy")
excluding_Latvia = filter(data_full, country != "Latvia")
excluding_Lithuania = filter(data_full, country != "Lithuania")
excluding_Luxembourg = filter(data_full, country != "Luxembourg")
excluding_Malta = filter(data_full, country != "Malta")
excluding_Netherlands = filter(data_full, country != "Netherlands")
excluding_Poland = filter(data_full, country != "Poland")
excluding_Portugal = filter(data_full, country != "Portugal")
excluding_Romania = filter(data_full, country != "Romania")
excluding_Slovakia = filter(data_full, country != "Slovakia")
excluding_Slovenia = filter(data_full, country != "Slovenia")
excluding_Spain = filter(data_full, country != "Spain")
excluding_Sweden = filter(data_full, country != "Sweden")
excluding_United_Kingdom = filter(data_full, country != "United Kingdom")

# Trust in the healthcare system
summary(glmer(data = excluding_United_Kingdom, donate_blood ~ age_std + gender + education_std + partner_status +
employment_status + parental_status + type_of_community + mean_confidence_healthcare_std + (1|country) + (1|year_EVS), family =
"binomial"))

# Healthcare quality
summary(glmer(data = excluding_United_Kingdom, donate_blood ~ age_std + gender + education_std + partner_status +
employment_status + parental_status + type_of_community + haq_index_std + health_exp_per_GDP_std + (1|country) + (1|year_haq) +
(1|year_health_exp), family = "binomial"))

# All results robust to exclusion of single countries EXCEPT HAQ index,
# which in 6 specifications has a significant positive effect:

# excluding_Italy: haq_index_std positive significant effect: b = 0.112 (0.056), p = 0.04396 *
# excluding_Latvia: haq_index_std positive significant effect: b = 0.121 (0.059), p = 0.03975 *
# excluding_Lithuania: haq_index_std positive significant effect: b = 0.135 (0.060), p = 0.02360 *
# excluding_Malta: haq_index_std positive significant effect: b = 0.126 (0.058), p = 0.02813 *
# excluding_Slovenia: haq_index_std positive significant effect: b = 0.116 (0.057), p = 0.04201 *
# excluding_Sweden: haq_index_std positive significant effect: b = 0.126 (0.057), p = 0.02755 *

##### d) Robustness check: Mean of country-level IVs aggregated over time

data_full$mean_confidence_healthcare_agg_no_time_std = as.numeric(scale(data_full$mean_confidence_healthcare_agg_no_time))
data_full$haq_agg_no_time_std = as.numeric(scale(data_full$haq_agg_no_time))
data_full$health_exp_per_GDP_agg_no_time_std = as.numeric(scale(data_full$health_exp_per_GDP_agg_no_time))

# a) Trust
m6 = glmer(donate_blood ~ age_std + gender + education_std + partner_status + employment_status + parental_status +
type_of_community + mean_confidence_healthcare_agg_no_time_std + (1|country) + (1|year_EVS), data = data_full, family =
"binomial")
summary(m6)
print(summary(m6),digits=2)
# Not significant

# b) Healthcare quality
m7 = glmer(donate_blood ~ age_std + gender + education_std + partner_status + employment_status + parental_status +
type_of_community + haq_agg_no_time_std + mean_confidence_healthcare_agg_no_time_std + (1|country) + (1|year_haq) +
(1|year_health_exp), data = data_full, family = "binomial")
summary(m7)
print(summary(m7),digits=2)
```

```
# Both not significant
```
